# Synthesis, biological evaluation, molecular modeling, and structural analysis of new pyrazole and pyrazolone derivatives as N‐formyl peptide receptors agonists

**DOI:** 10.1111/cbdd.13913

**Published:** 2021-07-01

**Authors:** Claudia Vergelli, Andrei I. Khlebnikov, Letizia Crocetti, Gabriella Guerrini, Niccolò Cantini, Liliya N. Kirpotina, Igor A. Schepetkin, Agostino Cilibrizzi, Mark T. Quinn, Patrizia Rossi, Paola Paoli, Maria Paola Giovannoni

**Affiliations:** ^1^ Neurofarba Pharmaceutical and Nutraceutical Section University of Florence Sesto Fiorentino Italy; ^2^ National Research Tomsk Polytechnic University Tomsk Russia; ^3^ Department of Microbiology and Immunology Montana State University Bozeman MT USA; ^4^ Institute of Pharmaceutical Science King’s College London London UK; ^5^ Department of Industrial Engineering University of Florence Florence Italy

**Keywords:** Agonist, cancer, formyl peptide receptor, inflammation, neutrophil, pyrazole, pyrazolone

## Abstract

N‐formyl peptide receptors (FPR1, FPR2, and FPR3) play key roles in the regulation of inflammatory processes, and recently, it was demonstrated that FPR1 and FPR2 have a dual role in the progression/suppression of some cancers. Therefore, FPRs represent an important therapeutic target for the treatment of both cancer and inflammatory diseases. Previously, we identified selective or mixed FPR agonists with pyridazinone or pyridinone scaffolds showing a common 4‐(bromophenyl)acetamide fragment, which was essential for activity. We report here new pyrazole and pyrazolone derivatives as restricted analogues of the above 6‐membered compounds, all exhibiting the same 4‐bromophenylacetamide side chain. Most new products had low or absent FPR agonist activity, suggesting that the pyrazole nucleus was not appropriate for FPR agonists. This hypothesis was confirmed by molecular modeling studies, which highlighted that the five‐membered scaffold was responsible for a worse arrangement of the molecules in the receptor binding site.

## Graphical abstract

N‐formyl peptide receptors (FPRs) play key roles in inflammation and cancer. The present paper reports a new series of pyrazole and pyrazolone derivatives as potential FPRs agonists, containing the 4‐(bromophenyl)acetamide fragment that was essential for activity in all our previous synthesized compounds. Molecular modeling studies, performed using the first FPR2 co‐crystallized protein, highlighted that the five‐member nucleous is responsible for a worse arrangement of the key fragment (4‐bromophenyl‐2‐acetamide chain) in the receptor binding site.

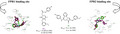

## 1 INTRODUCTION

The formyl peptide receptors (FPRs) are a family of G protein‐coupled receptors that play an important role in host defense and inflammation (Migeotte et al., 2006; Ye et al., 2009). Three different isoforms are known in humans: FPR1, FPR2, and FPR3. FPR1, the first receptor of the family to be identified, was discovered for its ability to transduce the chemotactic effect of a formylated bacterial product, formyl‐methionine‐leucyl‐phenylalanine (fMLF; Boulay et al., 1990). FPR1 is mainly expressed in cells of the immune system, such as neutrophils and monocytes/macrophages, but is also found in the lungs, brain, and gastrointestinal tract. FPR2 is distributed similarly to FPR1, but it is also present in hepatocytes, pancreas, glial cells, and astrocytes (Compernolle et al., 2003; Lacy et al., 1995; Uhlen et al., 2015). Finally, information about FPR3 is limited, and its role is not clear. It is present in monocytes, macrophages, and dendritic cells but not in neutrophils (Migeotte et al., 2006, 2005).

The primary role of FPR1 is the activation of chemotaxis in response to agonists, and recent studies have shown that it also contributes to direct phagocytosis of bacteria by neutrophils (Wen et al., 2019). Moreover, FPR1 and FPR2 have been shown to play a dual role in the progression/suppression of some types of cancer. For example, FPR1 is implicated in tumorigenesis and cell proliferation in glioblastoma and neuroblastoma (Cussel et al., 2019; Huang et al., 2009; Maris, 2010; Snapkov et al., 2016), and FPR2 may promote the malignancy of colon cancer (Xiang et al., 2016). Conversely, FPR1 and FPR2 have been shown to have tumor suppressor properties in gastric cancer (Prevete et al., 2005) and melanoma development (Liu et al., 2014), respectively. Interestingly, depending on the nature of the ligand, FPR2 can have an opposing effect on the inflammatory response, as some ligands can induce inflammatory processes to solve infection, while other ligands activate pro‐resolving and anti‐inflammatory pathways. Recently, it has been shown that the switch between FPR2‐mediated pro‐ and anti‐inflammatory cell responses is due to a conformational change of the receptor following ligand binding: In fact, the binding of anti‐inflammatory mediators such as annexin A1 (ANXA1) or lipoxin A4 (LXA4) results in FPR2 receptor homodimerization and in the release of inflammation‐resolving cytokines, neutrophil apoptosis, and macrophage efferocytosis (Sodin‐Semrl et al., 2004). Conversely, inflammatory ligands such as serum amyloid A do not cause receptor homodimerization, and their binding with FPR2 induces an increase in expression of pro‐inflammatory cytokines and chemokines (Cooray et al., 2013; Krepel & Wang, 2019).

**FIGURE 1:**
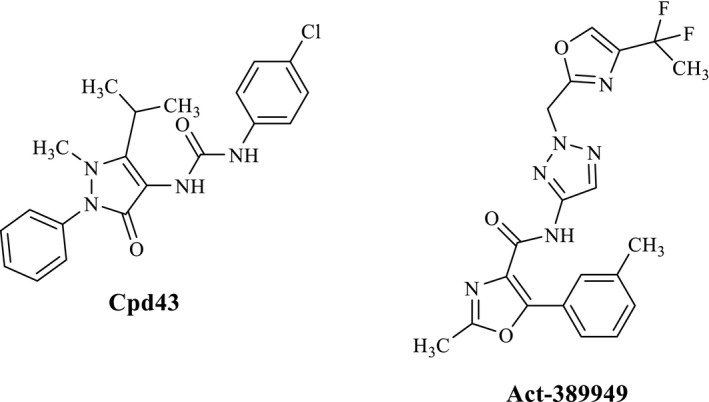
Structures of FPRs agonists

Currently, the FPR family represents an interesting pharmacological target for the treatment of some pathologies, such as inflammatory lung diseases, ischemia–reperfusion injury, neuroinflammation, and cancer (Bozinovski et al., 2013; Burli et al., 2006; Cussel et al., 2019; Huang et al., 2009; Liu et al., 2014; Maris, 2010; Perretti et al., 2015; Prevete et al., 2005, 2015; Snapkov et al., 2016; Xiang et al., 2016). Despite the great numbers of FPR ligands identified over the recent years, the pharmacological profile has been characterized in animal model studies for only a few compounds, and even less have reached clinical trials. In Figure 1 are reported two interesting compounds: **Cpd43**, synthesized by Amgen and exhibiting an excellent profile in animal models of rheumatoid arthritis (Burli et al., 2006; Odobasic et al., 2018), and compound **Act‐389949** which, recently tested in a phase I clinical trial, has been found to be safe and well tolerated in healthy human subject (Lind et al., 2019; Stalder et al., 2017).

**FIGURE 2:**
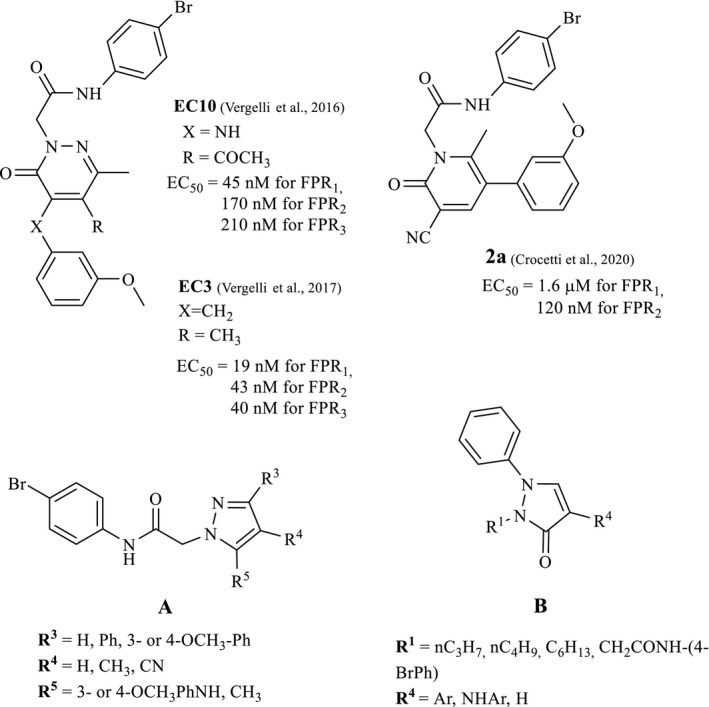
FPR agonists **EC3**, **EC10,** and **2a** and general structures of new products **A** and **B**

Our research in the field of FPR ligands led us to identify a large number of FPR agonists, including mixed FPR1/FPR2/FPR3 agonists, as well as FPR1‐ or FPR2‐selective agonists (Cilibrizzi et al., 2009, 2012, 2013; Crocetti et al., 2013; Giovannoni et al., 2013). They are small molecules with different scaffolds, all displaying a common N‐(4‐bromophenyl) acetamide fragment. The most potent compounds, with EC_50_ values in nanomolar or submicromolar range, are the pyridazin‐3(2H)‐one derivatives **EC10** (Vergelli et al., 2016), **EC3** (Vergelli et al., 2017), and the pyridinone **2a** (Crocetti et al., 2020; Figure 2). These three compounds were also evaluated in vivo in a rat model of rheumatoid arthritis and were found to exhibit anti‐inflammatory activity (Crocetti et al., 2020).

In the present study, we investigated further modification of these nitrogen heterocyclic scaffolds, in particular five‐membered pyrazoles (**A**) and pyrazolones (**B**) as restricted analogues of the previously synthesized pyridazinones (Vergelli et al., 2016, 2017) and pyridinones (Crocetti et al., 2020) bearing the same 4‐bromophenylacetamide side group. We also evaluated the effects of replacing the 4‐bromophenylacetamide side group with alkyl chains in new compounds of type **B** (Figure 2).

## 2 EXPERIMENTAL SECTION

### 2.1 Materials and methods

Reagents and starting materials were obtained from commercial sources. Extracts were dried over Na_2_SO_4_, and the solvents were removed under reduced pressure. All reactions were monitored by thin‐layer chromatography (TLC) using commercial plates precoated with Merck silica gel 60 F‐254. Visualization was performed by UV fluorescence (λ_max_ = 254 nm). Chromatographic separations were performed on a silica gel column by gravity chromatography (Kieselgel 40, 0.063–0.200 mm; Merck) or flash chromatography (Kieselgel 40, 0.040–0.063 mm; Merck). Yields refer to chromatographically and spectroscopically pure compounds, unless otherwise stated. Compounds were named following IUPAC rules, as applied by Beilstein‐Institut AutoNom 2000 (4.01.305) or CA Index Name. All melting points were determined on a microscope hot stage Büchi apparatus and are uncorrected. The identity and purity of intermediates and final compounds were determined through ^1^H NMR and TLC chromatography. ^1^H NMR and ^13^C NMR, HSQC, and HMBC spectra were recorded on an Avance 400 instruments (Bruker Biospin Version 002 with SGU, Bruker Inc.). Chemical shifts (*δ*) are reported in ppm to the nearest 0.01 ppm using solvent as the internal standard. Coupling constants (*J* values) are given in Hz and were calculated using ‘TopSpin 1.3’ software (Nicolet Instrument Corp., Madison, WI) and rounded to the nearest 0.1 Hz. Mass spectra (*m*/*z*) were recorded on a ESI‐MS triple quadrupole (Varian 1200L) system, in positive ion mode, by infusing a 10 mg/L solution of each analyte dissolved in a mixture of mQ H_2_O:acetonitrile 1:1 v/v. All new compounds had a purity ≥95%; microanalyses indicated by the symbols of the elements were performed with a Perkin‐Elmer 260 elemental analyzer for C, H, and N, and they were within ±0.4% of the theoretical values.

### 2.2 General procedure for the synthesis of new compounds

#### 2.2.1 General procedure for 3a‐c

A mixture of the commercially available appropriate intermediate of type **1** (**1a‐c**; 2.77 mmol), K_2_CO_3_ (5.44 mmol), and N‐(4‐bromophenyl)‐2‐chloroacetamide (**2**; Baraldi et al., 2007; 1.38–2.77 mmol) in CH_3_CN (15–20 ml) was refluxed under stirring for 3–7 hr. The suspension was then concentrated in vacuo, ice‐cold water was added, and the precipitate was recovered by suction. Final compounds were purified by column flash chromatography using as eluents hexane/ethyl acetate 1:2 for **3a**, CH_2_Cl_2_/ethyl acetate 7:3 for **3b**, and cyclohexane/ethyl acetate 1:3 for **3c**.

##### 2‐(5‐Amino‐4‐cyanopyrazol‐1‐yl)‐*N*‐(4‐bromophenyl)acetamide, **3a**

Yield = 18%; mp = 269–271°C (EtOH); ^1^H‐NMR (400 MHz, DMSO‐d_6_) δ 4.76 (s, 2H, NCH_2_), 6.63 (exch br s, 2H, NH_2_), 7.49 (m, 5H: 4H, Ar; 1H, pyrazole), 10.34 (exch br s, 1H, NH). ESI‐MS calcd. for C_12_H_10_BrN_5_O, 320.14; found: *m*/*z* 320.91 [*M* + H]^+^. Anal. Calcd. for C_12_H_10_BrN_5_O: C 45.02, H 3.15, N 21.88. Found: C 45.13, H 3.15, N 21.80.

##### 2‐(5‐Amino‐4‐methyl‐3‐phenylpyrazol‐1‐yl)‐*N*‐(4‐bromophenyl)acetamide, **3b**

Yield = 11%; mp = 171–174°C (EtOH); ^1^H‐NMR (400 MHz, DMSO‐d_6_) δ 1.99 (s, 3H, CH_3_), 4.78 (s, 2H, NCH_2_), 4.98 (exch br s, 2H, NH_2_), 7.25 (m, 1H, Ar), 7.35 (m, 2H, Ar), 7.48 (d, *J* = 8.8 Hz, 2H, Ar), 7.54 (m, 4H, Ar), 10.32 (exch br s, 1H, NH). ESI‐MS calcd. for C_18_H_17_BrN_4_O, 385.26; found: *m*/*z* 386.01 [*M* + H]^+^. Anal. Calcd. for. C_18_H_17_BrN_4_O: C 56.12, H 4.45, N 14.54. Found: C 56.31, H 4.46, N 14.48.

##### 2‐(5‐Aminopyrazol‐1‐yl)‐*N*‐(4‐bromophenyl)acetamide, **3c**

Yield = 17%; mp = 190–192°C (EtOH); ^1^H‐NMR (400 MHz, DMSO‐d_6_) δ 4.70 (s, 2H, NCH_2_), 5.16 (exch br s, 2H, NH_2_), 5.26 (d, *J* = 1.3 Hz, 1H, pyrazole), 7.04 (d, *J* = 1.3 Hz, 1H, pyrazole), 7.47 (d, *J* = 8.8 Hz, 2H, Ar), 7.53 (d, *J* = 8.8 Hz, 2H, Ar), 10.28 (exch br s, 1H, NH). ESI‐MS calcd. for C_11_H_11_BrN_4_O, 295.14; found: *m*/*z* 295.93 [*M* + H]^+^. Anal. Calcd. for. C_11_H_11_BrN_4_O: C 44.77, H 3.76, N 18.98. Found: C 44.89, H 3.75, N 18.91.

#### 2.2.2 General procedure for 4a‐e

A mixture of the appropriate intermediate of type **3** (**3a–c**; 0.31 mmol), 3‐ or 4‐methoxyphenylboronic acid (0.61–0.93 mmol), anhydrous cupric acetate (0.47 mmol), triethylamine (0.62 mmol), and activated molecular sieves (700 mg, 4 Å) in dry dichloromethane (10 ml) was stirred at room temperature for 2–26 hr. The suspension was filtered; the organic solution was washed with 15% aqueous ammonia (3 × 10 ml) and water (10 ml) and then dried over Na_2_SO_4_. After removal of the solvent under reduced pressure, the final compounds were purified by column flash chromatography using as eluents cyclohexane/ethyl acetate 1:3 for **4a**, dichloromethane/ethyl acetate 7:3 for **4b** and **4e**, dichloromethane/methanol 9.8:0.2 for **4c**, and dichloromethane/ethyl acetate 1:2 for **4d**.

##### *N*‐(4‐Bromophenyl)‐2‐[4‐cyano‐5‐(3‐methoxyphenylamino)pyrazol‐1‐yl]acetamide, **4a**

Yield = 23%; oil; ^1^H‐NMR (400 MHz, CDCl_3_) δ 3.77 (s, 3H, OCH_3_), 4.82 (s, 2H, NCH_2_), 6.46 (s, 1H, Ar), 6.51 (d, *J* = 7.6 Hz, 1H, Ar), 6.59 (d, *J* = 8.4 Hz, 1H, Ar), 6.99 (exch br s, 1H, NH), 7.20 (t, *J* = 8.0 Hz, 1H, Ar), 7.37 (d, *J* = 8.8 Hz, 2H, Ar), 7.44 (d, *J* = 8.4 Hz, 2H, Ar), 7.75 (s, 1H, pyrazole), 8.24 (exch br s, 1H, NH). ^13^C‐NMR (100 MHz, DMSO‐d_6_) δ 52.97, 55.48, 83.60, 104.01, 108.90, 110.52, 112.89, 118.51, 122.07, 130.79, 132.57, 135.59, 141.45, 142.65, 147.51, 160.81, 164.50. ESI‐MS calcd. for C_19_H_16_BrN_5_O_2_, 426.27; found: *m*/*z* 427.03 [*M* + H]^+^. Anal. Calcd. for. C_19_H_16_BrN_5_O_2_: C 53.54, H 3.78, N 16.43. Found: C 53.38, H 3.79, N 16.49.

##### *N*‐(4‐Bromophenyl)‐2‐[4‐cyano‐5‐(4‐methoxyphenylamino)pyrazol‐1‐yl]acetamide, **4b**

Yield = 24%; mp = 136–139°C (EtOH); ^1^H‐NMR (400 MHz, CDCl_3_) δ 3.78 (s, 3H, OCH_3_), 4.82 (s, 2H, NCH_2_), 6.87 (d, *J* = 8.4 Hz, 2H, Ar), 7.00 (d, *J* = 8.8 Hz, 2H, Ar), 7.12 (exch br s, 1H, NH), 7.40 (m, 2H, Ar), 7.45 (d, *J* = 8.4 Hz, 2H, Ar), 7.69 (s, 1H, pyrazole), 8.33 (exch br s, 1H, NH). ^13^C‐NMR (100 MHz, CDCl_3_) δ 52.95, 55.44, 83.58, 112.89, 115.03, 118.51, 122.07, 127.27, 132.57, 135.59, 141.45, 142.65, 147.51, 160.81, 164.50. ESI‐MS calcd. for C_19_H_16_BrN_5_O_2_, 426.27; found: *m*/*z* 427.06[*M* + H]^+^. Anal. Calcd. for. C_19_H_16_BrN_5_O_2_: C 53.54, H 3.78, N 16.43. Found: C 53.36, H 3.79, N 16.50.

##### *N*‐(4‐Bromophenyl)‐2‐[5‐(3‐methoxyphenylamino)‐4‐methyl‐3‐phenylpyrazol‐1‐yl]acetamide, **4c**

Yield = 26%; mp = >300°C dec. (EtOH); ^1^H‐NMR (400 MHz, DMSO‐d_6_) δ 1.97 (s, 3H, CH_3_), 3.63 (s, 3H, OCH_3_), 4.82 (s, 2H, NCH_2_), 6.20 (m, 2H, Ar), 6.26 (d, *J* = 8.4 Hz, 1H, Ar), 6.99 (m, 1H, Ar), 7.31 (m, 1H, Ar), 7.41 (m, 2H, Ar), 7.47 (m, 4H, Ar), 7.67 (m, 2H, Ar), 7.72 (exch br s, 1H, NH), 10.32 (exch br s, 1H, NH). ^13^C‐NMR (100 MHz, CDCl_3_) δ 9.51, 51.32, 55.19, 100.46, 105.27, 106.94, 109.88, 117.42, 121.61, 127.27, 128.16, 128.72, 130.46, 131.92, 133.17, 136.19, 139.77, 145.48, 150.93, 161.00, 165.40. ESI‐MS calcd. for C_25_H_23_BrN_4_O_2_, 491.38; found: *m*/*z* 492.09 [*M* + H]^+^. Anal. Calcd. for C_25_H_23_BrN_4_O_2_: C 61.11, H 4.72, N 11.40. Found: C 61.29, H 4.71, N 11.38.

##### *N*‐(4‐Bromophenyl)‐2‐[5‐(4‐methoxyphenylamino)‐4‐methyl‐3‐phenylpyrazol‐1‐yl]acetamide, **4d**

Yield = 40%; mp = 212–214°C (EtOH); ^1^H‐NMR (400 MHz, DMSO‐d_6_) δ 1.93 (s, 3H, CH_3_), 3.62 (s, 3H, OCH_3_), 4.82 (s, 2H, NCH_2_), 6.58 (d, *J* = 8.8 Hz, 2H, Ar), 6.73 (d, *J* = 8.8 Hz, 2H, Ar), 7.29 (m, 1H, Ar), 7.40 (m, 3H, Ar), 7.47 (m, 4H: 3H, Ar; 1H, exch br, NH), 7.67 (m, 2H, Ar), 10.32 (exch br s, 1H, NH). ^13^C‐NMR (100 MHz, DMSO‐d_6_) δ 9.49, 51.26, 55.37, 109.88, 115.03, 115.87, 117.42, 121.54, 127.27, 128.21, 128.73, 131.95, 133.17, 136.19, 139.77, 145.48, 150.92, 161.03, 165.40. ESI‐MS calcd. for C_25_H_23_BrN_4_O_2_, 491.38; found: *m*/*z* 492.05 [*M* + H]^+^. Anal. Calcd for C_25_H_23_BrN_4_O_2_: C 61.11, H 4.72, N 11.40. Found: C 61.30, H 4.71, N 11.40.

##### *N*‐(4‐Bromophenyl)‐2‐[5‐(3‐methoxyphenylamino)‐pyrazol‐1‐yl]acetamide, **4e**

Yield = 19%; mp = 140–143°C (EtOH); ^1^H‐NMR (400 MHz, DMSO‐d_6_) δ 3.65 (s, 3H, OCH_3_), 4.84 (s, 2H, NCH_2_), 6.01 (d, *J* = 1.2 Hz, 1H, pyrazole), 6.32 (d, *J* = 8.4 Hz, 1H, Ar), 6.41 (s, 1H, Ar), 6.44 (d, *J* = 7.6 Hz, 1H, Ar), 7.05 (t, *J* = 8.0 Hz, 1H, Ar), 7.39 (d, *J* = 1.2 Hz, 1H, pyrazole), 7.47 (d, *J* = 8.8 Hz, 2H, Ar), 7.52 (d, *J* = 8.8 Hz, 2H, Ar), 7.89 (exch br s, 1H, NH), 10.35 (exch br s, 1H, NH). ^13^C‐NMR (100 MHz, DMSO‐d_6_) δ 51.03, 56.01, 91.90, 100.81, 104.21, 107.62, 117.82, 122.71, 130.27, 132.11, 137.11, 139.32, 147.70, 151.12, 162.80, 168.20. ESI‐MS calcd. for C_18_H_17_BrN_4_O_2_, 401.26; found: *m*/*z* 401.97 [*M* + H]^+^. Anal. Calcd for C_18_H_17_BrN_4_O_2_: C 53.88, H 4.27, N 13.96. Found: C 53.70, H 4.26, N 13.91.

#### 2.2.3 General procedure for 6a,b

To a solution of **5a** or **5b** (Dal Piaz et al., 2004; 0.39 mmol) in EtOH (2 ml), 5 ml of 2.5 N NaOH was added, and the mixture was refluxed for 10 hr. After cooling, the solvent was evaporated under vacuum, ice‐cold water was added (10 ml), and the suspension was extracted with CH_2_Cl_2_ (3 × 10 ml). The organic layer was dried over Na_2_SO_4_ and evaporated under vacuum. The desired products **6a** and **6b** were obtained pure after recrystallization from EtOH.

##### 3‐(3‐Methoxyphenyl)‐5‐methyl‐1H‐pyrazole‐4‐carbonitrile, **6a**

Yield = 49%; mp = 139–141°C (EtOH); ^1^H‐NMR (400 MHz, CDCl_3_) δ 2.53 (s, 3H, CH_3_); 3.88 (s, 3H, OCH_3_); 7.03 (d, *J* = 8.4 Hz, 1H, Ar); 7.39 (m, 1H, Ar); 7.42 (s, 1H, Ar); 7.47 (m, 1H, Ar). ESI‐MS calcd. for C_12_H_11_N_3_O, 213.24; found: *m*/*z* 214.03 [*M* + H]^+^. Anal. Calcd for C_12_H_11_N_3_O: C 67.59, H 5.20, N 19.71. Found: C 67.36, H 5.22, N 19.67.

##### 3‐(4‐Methoxyphenyl)‐5‐methyl‐1H‐pyrazole‐4‐carbonitrile, **6b**

Yield = 31%; mp = 137–140°C (EtOH); ^1^H‐NMR (400 MHz, CDCl_3_) δ 2.48 (s, 3H, CH_3_); 3.86 (s, 3H, OCH_3_); 6.99 (d, *J* = 8.8 Hz, 2H, Ar); 7.79 (d, *J* = 8.8 Hz, 2H, Ar). ESI‐MS calcd. for C_12_H_11_N_3_O, 213.24; found: *m*/*z* 214.06 [*M* + H]^+^. Anal. Calcd for C_12_H_11_N_3_O: C 67.59, H 5.20, N 19.71. Found: C 67.40, H 5.21, N 19.73.

#### 2.2.4 General procedure for 7a,b

Compounds **7a,b** were obtained starting from **6a** and **6b** following the same procedure described for **3a‐c**. Final compounds were purified by crystallization from EtOH.

##### *N*‐(4‐Bromophenyl)‐2‐[4‐cyano‐3‐(3‐methoxyphenyl)‐5‐methylpyrazol‐1‐yl]acetamide, **7a**

Yield = 54%; mp = 170–173°C (EtOH); ^1^H‐NMR (400 MHz, DMSO‐d_6_) δ 2.44 (s, 3H, CH_3_); 3.78 (s, 3H, OCH_3_); 5.14 (s, 2H, CH_2_); 7.01 (m, 1H, Ar); 7.34 (exch br s, 1H, NH); 7.41 (m, 2H, Ar); 7.51 (m, 5H, Ar). ^13^C‐NMR (100 MHz, DMSO‐d_6_) δ 10.92; 53.36; 55.61; 88.97; 111.62; 115.41; 115.90; 118.67; 121.71; 130.68; 132.19; 132.32; 138.29; 150.01; 150.82; 160.01; 164.99. ESI‐MS calcd. for C_20_H_17_BrN_4_O_2_, 425.28; found: *m*/*z* 426.05 [*M* + H]^+^. Anal. Calcd for C_20_H_17_BrN_4_O_2_: C 56.48, H 4.03, N 13.17. Found: C 56.69, H 4.02, N 13.21.

##### N‐(4‐Bromophenyl)‐2‐[4‐cyano‐3‐(4‐methoxyphenyl)‐5‐methylpyrazol‐1‐yl]acetamide, **7b**

Yield = 32%; mp = 171–174°C (EtOH); ^1^H‐NMR (400 MHz, DMSO‐d_6_) δ 2.42 (s, 3H, CH_3_); 3.78 (s, 3H, OCH_3_); 5.11 (s, 2H, CH_2_); 7.04 (d, *J* = 8.8 Hz, 2H, Ar); 7.51 (m, 4H, Ar); 7.76 (d, *J* = 8.8 Hz, 2H, Ar); 10.57 (exch br s, 1H, NH); ^13^C‐NMR (DMSO‐d_6_) δ 10.89; 53.26; 55.74; 114.91; 115.63; 115.94; 121.72; 123.59; 127.83; 132.21; 138.18; 149.74; 151.00; 160.47; 165.05. ESI‐MS calcd. for C_20_H_17_BrN_4_O_2_, 425.28; found: *m*/*z* 426.02 [*M* + H]^+^. Anal. Calcd for C_20_H_17_BrN_4_O_2_: C 56.48, H 4.03, N 13.17. Found: C 56.67, H 4.03, N 13.20.

#### 2.2.5 General procedures for 9a,b

Compounds **9a,b** were obtained starting from the appropriate substrate of type **8** (**8a** commercially available, **8b** previously reported (O’Brain & Gates, 1966) following the same procedure described for **3a‐c**. Final compounds were purified by column flash chromatography using as eluents cyclohexane/ethyl acetate 3:2 for **9a** and cyclohexane/ethyl acetate 1:3 for **9b**.

##### *N*‐(4‐Bromophenyl)‐2‐(5‐oxo‐2‐phenyl‐2,5‐dihydropyrazol‐1‐yl)acetamide, **9a**

Yield = 31%; mp = 136–139°C (EtOH); ^1^H‐NMR (400 MHz, DMSO‐d_6_) δ 4.82 (s, 2H, NCH_2_); 6.07 (d, *J* = 2.8 Hz, 1H, pyrazolone); 7.19 (t, *J* = 7.4 Hz, 1H, Ar); 7.41 (t, *J* = 7.8 Hz, 2H, Ar); 7.48 (d, *J* = 8.8 Hz, 2H, Ar); 7.59 (d, *J* = 8.8 Hz, 2H, Ar); 7.68 (d, *J* = 8.8 Hz, 2H, Ar); 8.34 (d, *J* = 2.8 Hz, 1H, pyrazolone,); 10.26 (exch br s, 1H, NH). ^13^C‐NMR (100 MHz, DMSO‐d_6_) δ 52.71; 101.10; 112.27; 118.88; 119.01; 123.11; 128.88; 132.11; 139.81; 142.18; 164.40; 169.01. ESI‐MS calcd. for C_17_H_14_BrN_3_O_2_, 372.22; found: *m*/*z* 372.94 [*M* + H]^+^. Anal. Calcd for C_17_H_14_BrN_3_O_2_: C 54.86, H 3.79, N 11.29. Found: C 55.05, H 3.78, N 11.33.

##### 2‐(4‐Amino‐5‐oxo‐2‐phenyl‐2,5‐dihydropyrazol‐1‐yl)‐*N*‐(4‐bromophenyl)acetamide, **9b**

Yield = 23%; mp = 160–163°C (EtOH); ^1^H‐NMR (400 MHz, DMSO‐d_6_) δ 4.03 (exch br s, 2H, NH_2_); 4.80 (s, 2H, NCH_2_); 7.04 (t, *J* = 7.2 Hz, 1H, Ar); 7.33 (t, *J* = 7.6 Hz, 2H, Ar); 7.51 (m, 4H, Ar); 7.62 (m, 3H: 2H, Ar; 1H, pyrazole); 10.11 (exch br s, 1H, NH). ESI‐MS calcd. for C_17_H_15_BrN_4_O_2_, 387.23; found: *m*/*z* 387.99 [*M* + H]^+^. Anal. Calcd for C_17_H_15_BrN_4_O_2_: C 52.73, H 3.90, N 14.47. Found: C 52.91, H 3.90, N 14.52.

##### *N*‐(4‐Bromophenyl)‐2‐[4‐(3‐methoxyphenylamino)‐5‐oxo‐2‐phenyl‐2,5‐dihydropyrazol‐1‐yl]acetamide, **10**

Compound **10** was obtained starting from **9b** following the same procedure described for **4a‐e**. The desired final compound was purified by column flash chromatography using toluene/methanol 9:1 as eluent.

Yield = 21%; mp = 163–166°C (EtOH); ^1^H‐NMR (400 MHz, DMSO‐d_6_) δ 3.66 (s, 3H, OCH_3_); 4.88 (s, 2H, NCH_2_); 6.22 (m, 1H, Ar); 6.34 (s, 1H, Ar); 6.44 (d, *J* = 8.0 Hz, 1H, Ar); 7.02 (t, *J* = 8.2 Hz, 1H, Ar); 7.13 (t, *J* = 7.2 Hz, 1H, Ar); 7.39 (m, 3H: 2H, Ar; exch br, 1H, NH); 7.49 (d, *J* = 8.8 Hz, 2H, Ar); 7.58 (d, *J* = 8.8 Hz, 2H, Ar); 7.68 (d, *J* = 8.4 Hz, 2H, Ar); 8.32 (s, 1H, pyrazolone); 10.23 (exch br s, 1H, NH). ^13^C‐NMR (100 MHz, DMSO‐d_6_) δ 55.18; 67.75; 99.48; 103.35; 106.64; 112.60; 115.67; 116.78; 121.86; 122.34; 124.99; 129.80; 130.20; 132.11; 138.34; 140.11; 148.00; 157.35; 160.77; 166.96. ESI‐MS calcd. for C_24_H_21_BrN_4_O_3_, 493.35; found: *m*/*z* 494.11 [*M* + H]^+^. Anal. Calcd for C_24_H_21_BrN_4_O_3_: C 58.43, H 4.29, N 11.36. Found: C 58.64, H 4.30, N 11.33.

#### 2.2.6 General procedure for 12a‐c

To a suspension of **11** (O’Brain & Gates, 1966; 0.42 mmol) and K_2_CO_3_ (0.84 mmol) in 1.5 ml of anhydrous DMF, 0.63 mmol of the appropriate alkyl bromide was added, and the mixture was refluxed for 1 hr. After cooling, ice‐cold water was added (15 ml), and the suspension was extracted with CH_2_Cl_2_ (3 × 10 ml). The organic layer was dried over Na_2_SO_4_ and evaporated under vacuum. Compounds **12a‐c** were purified by column flash chromatography using cyclohexane/ethyl acetate 2:1 as eluent.

##### 4‐Bromo‐1‐phenyl‐2‐n.propyl‐1,2‐dihydropyrazol‐3‐one, **12a**

Yield = 26%; oil; ^1^H‐NMR (400 MHz, CDCl_3_) δ 1.04 (t, *J* = 7.4 Hz, 3H, *N*(CH_2_)_2_ *CH_3_*); 1.83–1.88 (m, 2H, NCH_2_ *CH_2_* CH_3_); 4.27 (t, *J* = 6.8 Hz, 2H, N*CH_2_* CH_2_CH_3_); 7.43 (m, 2H, Ar); 7.51 (m, 3H, Ar); 7.74 (s, 1H, pyrazolone). ESI‐MS calcd. for C_12_H_13_BrN_2_O, 281.15; found: *m*/*z* 281.92 [*M* + H]^+^. Anal. Calcd for C_12_H_13_BrN_2_O: C 51.26, H 4.66, N 9.96. Found: C 51.40, H 4.65, N 9.92.

##### 4‐Bromo‐2‐n.butyl‐1‐phenyl‐1,2‐dihydropyrazol‐3‐one, **12b**

Yield = 64%; oil; ^1^H‐NMR (400 MHz, CDCl_3_) δ 0.98 (m, 3H, *N*(CH_2_)_3_ *CH_3_*); 1.50 (m, 2H, *N*(CH_2_)_2_ *CH_2_* CH_3_); 1.80 (m, 2H, NCH_2_ *CH_2_* CH_2_CH_3_); 4.30 (m, 2H, N*CH_2_* (CH_2_)_2_CH_3_); 7.42 (m, 2H, Ar); 7.50 (m, 3H, Ar); 7.72 (s, 1H, pyrazolone). ESI‐MS calcd. for C_13_H_15_BrN_2_O, 295.17; found: *m*/*z* 295.92 [*M* + H]^+^. Anal. Calcd for C_13_H_15_BrN_2_O: C 52.90, H 5.12, N 9.49. Found: C 52.72, H 5.10, N 9.51.

##### 4‐Bromo‐2‐n.hexyl‐1‐phenyl‐1,2‐dihydropyrazol‐3‐one, 12c

Yield = 55%; oil; ^1^H‐NMR (400 MHz, CDCl_3_) δ 0.90 (t, *J* = 6.8 Hz, 3H, *N*(CH_2_)_5_ *CH_3_*); 1.34 (m, 4H, *N*(CH_2_)_3_ *CH_2_CH_2_* CH_3_); 1.45 (m, 2H, *N*(CH_2_)_2_ *CH_2_* (CH_2_)_2_CH_3_); 1.78–1.84 (m, 2H, NCH_2_ *CH_2_* (CH_2_)_3_CH_3_); 4.29 (t, *J* = 6.8 Hz, 2H, N*CH_2_* (CH_2_)_4_CH_3_); 7.43 (d, *J* = 8.8 Hz, 2H, Ar); 7.51 (m, 3H, Ar); 7.74 (s, 1H, pyrazolone). ESI‐MS calcd. for C_15_H_19_BrN_2_O, 323.23; found: *m*/*z* 323.95 [*M* + H]^+^. Anal. Calcd for C_15_H_19_BrN_2_O: C 55.74, H 5.92, N 8.67. Found: C 55.92, H 5.90, N 8.65.

#### 2.2.7 General procedure for 13a‐f

To a suspension of suitable substrate of type **12** (**12a–c**; 0.43 mmol) and 0.042 mmol of tetrakis(triphenylphosphine)palladium(0) in anhydrous toluene (5 ml), a solution of the appropriate arylboronic acid (0.86–3.44 mmol) in ethanol (1–3 ml) and 3.3 ml of 2 M Na_2_CO_3_ were added. The mixture was stirred at room temperature for 15–18 hr for compounds **13a** and **13d**, whereas the suspension was refluxed for 6–12 hr for compounds **13b,c,e,f**. After evaporation of the solvent under vacuum, ice‐cold water was added (10–15 ml) and the mixture was extracted with CH_2_Cl_2_ (3 × 10 ml). For compound **13c**, the suspension was first neutralized with 6N HCl and then extracted with ethyl acetate (3 × 10 ml). Finally, the organic layer was dried over Na_2_SO_4_ and evaporated in *vacuo*. Final compounds were purified by column flash chromatography using as eluents hexane/ethyl acetate 7:0.2 for **13a**, cyclohexane/ethyl acetate 4:1 for **13b** and **13e**, CH_2_Cl_2_/CH_3_OH 9:1 for **13c**, hexane/ethyl acetate 6:0.2 for **13d**, and toluene/ethyl acetate 7:0.2 for **13f**.

##### 4‐(3‐Methoxyphenyl)‐1‐phenyl‐2‐n.propyl‐1,2‐dihydropyrazol‐3‐one, **13a**

Yield = 16%; oil; ^1^H‐NMR (400 MHz, CDCl_3_) δ 1.05 (t, *J* = 7.4 Hz, 3H, *N*(CH_2_)_2_ *CH_3_*); 1.84–1.90 (m, 2H, NCH_2_ *CH_2_* CH_3_); 3.87 (s, 3H, OCH_3_); 4.30 (t, *J* = 6.6 Hz, 2H, N*CH_2_* CH_2_CH_3_ ); 6.90 (d, *J* = 8.2 Hz, 1H, Ar); 7.11 (s, 1H, Ar); 7.17 (d, *J* = 7.6 Hz, 1H, Ar); 7.36 (t, *J* = 8.0 Hz, 1H, Ar); 7.52 (s, 1H, Ar); 7.62 (s, 4H, Ar); 7.80 (s, 1H, pyrazolone). ^13^C‐NMR (100 MHz, CDCl_3_) δ 10.39; 22.43; 55.34; 71.31; 112.68; 117.81; 118.99; 119.41; 127.52; 128.10; 129.89; 132.34; 138.40; 135.10; 141.64; 160.03; 160.97. ESI‐MS calcd. for C_19_H_20_N_2_O_2_, 308.37; found: *m*/*z* 309.14 [*M* + H]^+^. Anal. Calcd for C_19_H_20_N_2_O_2_: C 74.00, H 6.54, N 9.08. Found: C 74.25, H 6.53, N 6.51.

##### 4‐(3,4‐Dimethoxyphenyl)‐1‐phenyl‐2‐n.propyl‐1,2‐dihydropyrazol‐3‐one, **13b**

Yield = 12%; oil; ^1^H‐NMR (400 MHz, CDCl_3_) δ 1.05 (t, *J* = 7.4 Hz, 3H, *N*(CH_2_)_2_ *CH_3_*); 1.84–1.90 (m, 2H, NCH_2_ *CH_2_* CH_3_); 3.92 (s, 3H, OCH_3_); 3.96 (s, 3H, OCH_3_); 4.30 (t, *J* = 6.8 Hz, 2H, N*CH_2_* CH_2_CH_3_); 6.95 (d, *J* = 8.4 Hz, 1H, Ar); 7.09 (s, 1H, Ar); 7.14 (dd, *J* = 8.4 Hz, *J* = 2 Hz, 1H, Ar); 7.52–7.60 (m, 5H, Ar); 7.80 (s, 1H, pyrazolone). ^13^C‐NMR (100 MHz, CDCl_3_) δ 14.14; 31.05; 56.01; 71.32; 110.22; 111.58; 117.91; 119.26; 127.73; 132.38; 137.73; 138.47; 149.05; 149.58; 160.57. ESI‐MS calcd. for C_20_H_22_N_2_O_3_, 338.40; found: *m*/*z* 339.15 [*M* + H]^+^. Anal. Calcd for C_20_H_22_N_2_O_3_: C 70.99, H 6.55, N 8.28. Found: C 70.78, H 6.54, N 8.29.

##### 4‐(4‐Hydroxyphenyl)‐1‐phenyl‐2‐n.propyl‐1,2‐dihydropyrazol‐3‐one, **13c**

Yield = 14%; oil; ^1^H‐NMR (400 MHz, CDCl_3_) δ 0.93 (t, *J* = 7.4 Hz, 3H, *N*(CH_2_)_2_ *CH_3_*); 1.65–1.71 (m, 2H, NCH_2_ *CH_2_* CH_3_); 4.11 (t, *J* = 6.8 Hz, 2H, N*CH_2_* CH_2_CH_3_); 7.08 (d, *J* = 8.4 Hz, 2H, Ar); 7.41 (d, *J* = 8.8 Hz, 2H, Ar); 7.49 (m, 4H, Ar); 7.64 (s, 1H, Ar); 7.94 (s, 1H, pyrazolone). ^13^C‐NMR (100 MHz, CDCl_3_) δ 11.20; 18.62; 46.23; 115.81; 122.84; 123.90; 125.13; 129.22; 130.53; 134.51; 137.55; 141.12; 157.74; 164.12. ESI‐MS calcd. for C_18_H_18_N_2_O_2_, 294.35; found: *m*/*z* 295.07 [*M* + H]^+^. Anal. Calcd for C_18_H_18_N_2_O_2_: C 73.45, H 6.16, N 9.52. Found: C 73.64, H 6.17, N 9.49.

##### Butyl‐4‐(3‐methoxyphenyl)‐1‐phenyl‐1,2‐dihydropyrazol‐3‐one, **13d**

Yield = 12%; oil; ^1^H‐NMR (400 MHz, CDCl_3_) δ 0.99 (t, *J* = 7.4 Hz, 3H, NCH_2_CH_2_CH_2_ *CH_3_*); 1.50 (m, 2H, NCH_2_CH_2_ *CH_2_* CH_3_); 1.83 (m, 2H, NCH_2_ *CH_2_* CH_2_CH_3_); 3.87 (s, 3H, OCH_3_); 4.34 (t, *J* = 6.6 Hz, 2H, N*CH_2_* CH_2_CH_2_CH_3_); 6.90 (d, *J* = 8.2 Hz, 1H, Ar); 7.11 (s, 1H, Ar); 7.17 (d, *J* = 7.6 Hz, 1H, Ar); 7.36 (t, *J* = 7.8 Hz, 1H, Ar); 7.52 (s, 1H, Ar); 7.62 (s, 4H, Ar); 7.80 (s, 1H, pyrazolone). ^13^C‐NMR (100 MHz, CDCl_3_) δ 13.89; 19.14; 31.16; 55.35; 69.60; 112.78; 117.81; 119.00; 127.52; 128.11; 129.89; 138.41; 139.10; 141.64; 160.02; 160.96. ESI‐MS calcd. for C_20_H_22_N_2_O_2_, 322.40; found: *m*/*z* 323.16 [*M* + H]^+^. Anal. Calcd for C_20_H_22_N_2_O_2_: C 74.51, H 6.88, N 8.69. Found: C 74.73, H 6.87, N 8.71.

##### Butyl‐4‐(3,4‐dimethoxyphenyl)‐1‐phenyl‐1,2‐dihydropyrazol‐3‐one, **13e**

Yield = 10%; oil; ^1^H‐NMR (400 MHz, CDCl_3_) δ 0.99 (t, *J* = 7.4 Hz, 3H, NCH_2_CH_2_CH_2_ *CH_3_*); 1.48–1.54 (m, 2H, NCH_2_CH_2_ *CH_2_* CH_3_); 1.79–1.86 (m, 2H, NCH_2_ *CH_2_* CH_2_CH_3_); 3.92 (s, 3H, OCH_3_); 3.95 ( s, 3H, OCH_3_); 4.34 ( t, *J* = 6.6 Hz, 2H, N*CH_2_* CH_2_CH_2_CH_3_); 6.94 (d, *J* = 8.4 Hz, 1H, Ar,); 7.09 (s, 1H, Ar); 7.14 (d, *J* = 8.4 Hz, 1H, Ar); 7.52 (s, 1H, Ar); 7.59 (s, 4H, Ar); 7.79 (s, 1H, pyrazolone). ^13^C‐NMR (100 MHz,CDCl_3_) δ 14.14; 19.08; 31.05; 56.07; 69.48; 110.75; 112.69; 117.77; 119.15; 127.73; 132.38; 137.77; 138.47; 149.05; 149.58; 160.57. ESI‐MS calcd. for C_21_H_24_N_2_O_3_, 352.43; found: *m*/*z* 353.19 [*M* + H]^+^. Anal. Calcd for C_21_H_24_N_2_O_3_: C 71.57, H 6.86, N 7.95. Found: C 71.39, H 6.85, N 7.93.

##### Hexyl‐4‐(3‐methoxyphenyl)‐1‐phenyl‐1,2‐dihydropyrazol‐3‐one, **13f**

Yield = 13%; oil; ^1^H‐NMR (400 MHz, CDCl_3_) δ 0.91 (t, *J* = 7 Hz, 3H, *N*(CH_2_)_5_ *CH_3_*); 1.36 (m, 4H, *N*(CH_2_)_3_ *CH_2_CH_2_* CH_3_); 1.47 (m, 2H, *N*(CH_2_)_2_ *CH_2_* (CH_2_)_2_CH_3_); 1.81–1.88 (m, 2H, NCH_2_ *CH_2_* (CH_2_)_3_CH_3_); 3.87 (s, 3H, OCH_3_); 4.33 (t, *J* = 6.6 Hz, 2H, N*CH_2_* (CH_2_)_4_CH_3_); 6.90 (d, *J* = 8.4 Hz, 1H, Ar); 7.11 (m, 1H, Ar); 7.17 (d, *J* = 8 Hz, 1H, Ar); 7.36 (t, *J* = 7.8 Hz, 1H, Ar); 7.62 (s, 5H, Ar); 7.80 (s, 1H, pyrazolone). ^13^C‐NMR (100 MHz, CDCl_3_) δ 14.04; 22.60; 25.56; 29.03; 31.56; 55.35; 69.92; 112.69; 117.81; 119.41; 127.51; 128.10; 131.91; 138.41; 139.11; 141.65; 160.04; 160.99. ESI‐MS calcd. for C_22_H_26_N_2_O_2_, 350.45; found: *m*/*z* 351.26 [*M* + H]^+^. Anal. Calcd for C_22_H_26_N_2_O_2_: C 75.40, H 7.48, N 7.99. Found: C 75.65, H 7.47, N 7.96.

#### 2.2.8 General procedure for 15 and 16

Compounds **15** and **16** were obtained as a mixture starting from commercially available **14**, following the same procedure described for **3a‐c**. The mixture was separated by column flash chromatography using as eluent cyclohexane/ethyl acetate 1:3, and compounds **15** and **16** recovered pure.

##### N‐(4‐bromophenyl)‐2‐[(5‐methyl‐1H‐pyrazol‐3‐yl)oxy]acetamide, **15**

Yield = 12%; 183–185°C (EtOH); ^1^H‐NMR (400 MHz, DMSO‐d_6_) δ 2.11 (s, 3H, CH_3_); 4.62 (s, 2H, OCH_2_); 5.46 (s, 1H, pyrazolo); 7.45 (d, *J* = 8.8 Hz, 2H, Ar); 7.58 (d, *J* = 8.8 Hz, 2H, Ar); 10.09 (exch br s, 1H, NH); 11.56 (exch br s, 1H, NH). ^13^C‐NMR (100 MHz DMSO‐d_6_) δ 11.43, 67.57, 89.31, 115.58, 121.91, 131.96, 138.36, 140.50, 162.50, 167.67. ESI‐MS calcd. for C_12_H_12_BrN_3_O_2_, 310.15; found: *m*/*z* 310.94 [*M* + H]^+^. Anal. Calcd for C_12_H_12_BrN_3_O_2_: C 46.67, H 3.90, N 13.55. Found: C 46.53, H 3.89, N 13.51.

##### N‐(4‐bromophenyl)‐2‐{3‐[2‐(4‐bromophenyl)amino]‐2‐oxoethoxy}‐5‐methyl‐1H‐pyrazol‐1‐yl)acetamide, **16**

Yield = 20%; 123–124°C (EtOH); ^1^H‐NMR (400 MHz, DMSO‐d_6_) δ 2.16 (s, 3H, CH_3_); 4.62 (s, 2H, OCH_2_); 4.72 (s, 2H, NCH_2_); 5.57 (s, 1H, pyrazolo); 7.49 (m, 8H, Ar); 10.09 (exch br s, 1H, NH); 10.29 (exch br s, 1H, NH). ^13^C‐NMR (100 MHz DMSO‐d_6_) δ 11.44, 52.06, 67.62, 90.97, 115.59, 121.59, 121.94, 131.91, 132.06, 138.45, 142.41, 161.24, 166.32, 167.29. ESI‐MS calcd. for C_20_H_18_Br_2_N_4_O_3_, 522.20; found: *m*/*z* 522.92 [*M* + H]^+^. Anal. Calcd for C_20_H_18_Br_2_N_4_O_3_: C, 56.48; H, 4,03; N, 13,17. Found: C, 56.60, H, 4.02, N, 13.15.

### 2.3 Biological assays

#### 2.3.1 Cell culture

Human promyelocytic leukemia HL60 cells stably transfected with FPR1 (FPR1‐HL60 cells) or FPR2 (FPR2‐HL60 cells; kind gift from Dr. Marie‐Josephe Rabiet, Université Joseph Fourier, Grenoble, France) were cultured in RPMI 1640 medium supplemented with 10% heat‐inactivated fetal calf serum, 10 mM HEPES, 100 μg/ml streptomycin, 100 U/ml penicillin, and G418 (1 mg/ml). Although stable cell lines are cultured under G418 selection pressure, G418 may affect some assays, so it was removed in the last round of culture before assays were performed.

#### 2.3.2 Isolation of human neutrophils

Blood was collected from healthy donors in accordance with a protocol approved by the Institutional Review Board at Montana State University. Neutrophils were purified from the blood using dextran sedimentation, followed by Histopaque 1077 gradient separation and hypotonic lysis of red blood cells. Isolated neutrophils were washed twice and resuspended in HBSS^‐^. Neutrophil preparations were routinely >95% pure, as determined by light microscopy, and >98% viable, as determined by trypan blue exclusion.

#### 2.3.3 Ca^2+^ Mobilization assay

Changes in intracellular Ca^2+^ were measured with a FlexStation II scanning fluorometer (Molecular Devices). The cells, suspended in Hank's balanced salt solution without Ca^2+^ and Mg^2+^ but with 10 mM HEPES (HBSS^‐^), were loaded with 1.25 μg/ml Fluo‐4 a.m. dye and incubated for 30 min in the dark at 37°C. After dye loading, the cells were washed with HBSS^‐^ containing 10 mM HEPES, resuspended in HBSS^+^ containing Ca^2+^, Mg^2+^, and 10 mM HEPES (HBSS^+^), and aliquoted into the wells of flat‐bottom, half‐area‐well black microtiter plates (2 × 10^5^ cells/well). For evaluation of direct agonist activity, compounds of interest were added from a source plate containing dilutions of test compounds in HBSS^+^, and changes in fluorescence were monitored (λ_ex_ = 485 nm, λ_em_ = 538 nm) every 5 s for 240 s at room temperature after automated addition of compounds. Maximum change in fluorescence during the first 3 min, expressed in arbitrary units over baseline, was used to determine a response. Responses for FPR1 agonists were normalized to the response induced by 5 nM fMLF for FPR1‐HL60 cells and neutrophils, or 5 nM WKYMVm for FPR2‐HL60 cells, which were assigned a value of 100%. Curve fitting (5–6 points) and calculation of median effective inhibitory concentrations (IC_50_) were performed by nonlinear regression analysis of the dose–response curves generated using Prism 7 (GraphPad Software, Inc.).

### 2.4 Single‐crystal X‐ray diffraction (SCXRD)

Intensity data for compound **15** were collected at 100 K using a Bruker Apex‐II CCD diffractometer. Data were collected with the Bruker APEX2 program (Bruker, 2012a) and integrated and reduced with Bruker SAINT software (Bruker, 2012b); absorption correction was performed with SADABS‐2016/2 (Krause et al., 2015). The radiation used was Cu−Kα (λ = 1.54184 Å). The crystal structure was solved using the SHELXS‐97 program (Sheldrick, 2008) and refined by full‐matrix least squares against *F* ^2^ using all data (SHELXL‐2018/3 (Sheldrick, 2015). All non‐hydrogen atoms were refined with anisotropic displacement parameters, while the hydrogen atoms were found in the Fourier density map. Their co‐ordinates were freely refined while their thermal parameters were set in accordance with one of the atoms to which they are bonded. Geometrical calculations were performed by PARST97 (Nardelli, 1995) and molecular plots were produced by the program Mercury (v4.3.1; Macrae et al., 2008) and ORTEP‐3 (Farrugia, 1997). Crystallographic data and refinement parameters are reported in Table S2 (see Supporting Information).

### 2.5 Molecular modeling procedures

Structures of compounds **EC3**, **EC10**, **2a**, **4e**, **7a**, **10**, and **15** were built using ChemOffice 2016 software, pre‐optimized with the MM2 force field, and saved in Tripos MOL2 format. A homology model of FPR1 with docked fMLF (Zhuang et al., 2020) and a cryo‐EM structure of FPR2‐G_i_ complex with the peptide agonist Trp‐Lys‐Tyr‐Met‐Val‐D‐Met‐NH_2_ (WKYMVm; Zhuang et al., 2020; PDB entry 6OMM) was taken as sources of the receptor geometries for the docking study. Each of the receptor structures was then imported into the Molegro Virtual Docker 6.0 program (MVD) together with the built models of ligands **EC3**, **EC10**, **2a**, **4e**, **7a**, **10**, and **15**. A search space for docking was defined as a sphere 12 Å in radius located at the geometric center of gravity of the bound peptide molecule (fMLF for FPR1 or WKYMVm for FPR2). MolDock score functions were applied with a 0.3 Å grid resolution. Flexibility of ligands was accounted for with respect to torsions auto‐detected in MVD. The receptor structures were considered rigid. The “Internal HBond” and “sp^2^‐sp^2^ torsions” options were activated in the “Ligand evaluation” panel of the MVD Docking Wizard. Three hundred docking runs were performed for each investigated compound with each receptor. The option “Return multiple poses for each run” was enabled, and the post‐processing options “Energy minimization” and “Optimize H‐bonds” were applied after docking. Similar poses were clustered at a RMSD threshold of 1 Å.

## 3 RESULTS AND DISCUSSION

### 3.1 Chemistry

**SCHEME 1:**
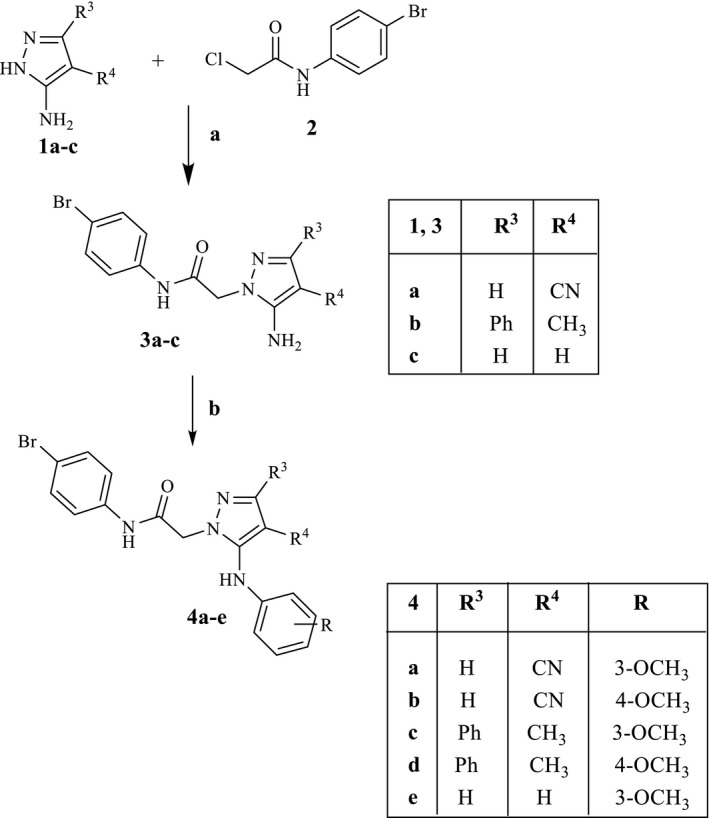
Reagents and conditions: (a) K_2_CO_3_, anhydrous CH_3_CN, reflux, 3–7 hr. (b) 3‐ or 4‐Methoxybenzeneboronic acid, (CH_3_COO)_2_Cu, Et3N, activated molecular sieves, anhydrous CH_2_Cl_2_, rt, 2–26 hr

**SCHEME 2:**
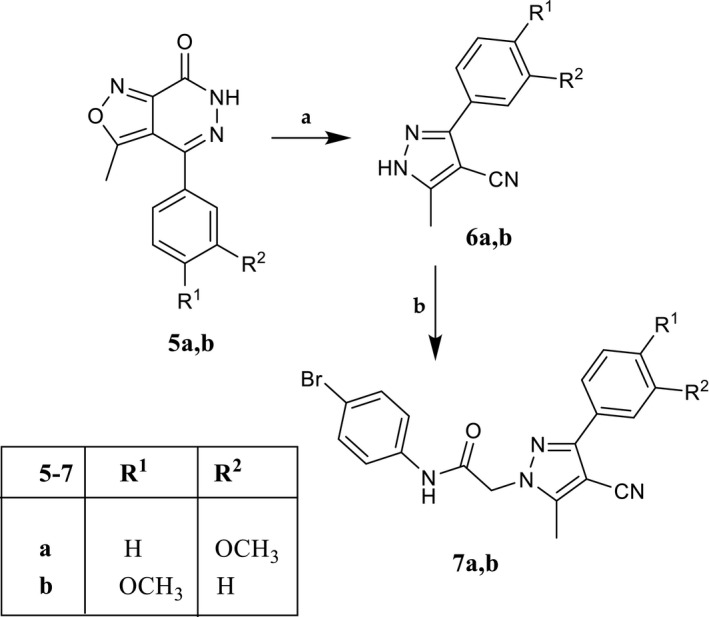
Reagents and conditions: (a) 2.5 N NaOH, EtOH, reflux, 10 hr. (b) N‐(4‐bromophenyl)‐2‐chloroacetamide (**2**), K_2_CO_3_, anhydrous CH_3_CN, reflux, 3 hr

**SCHEME 3:**
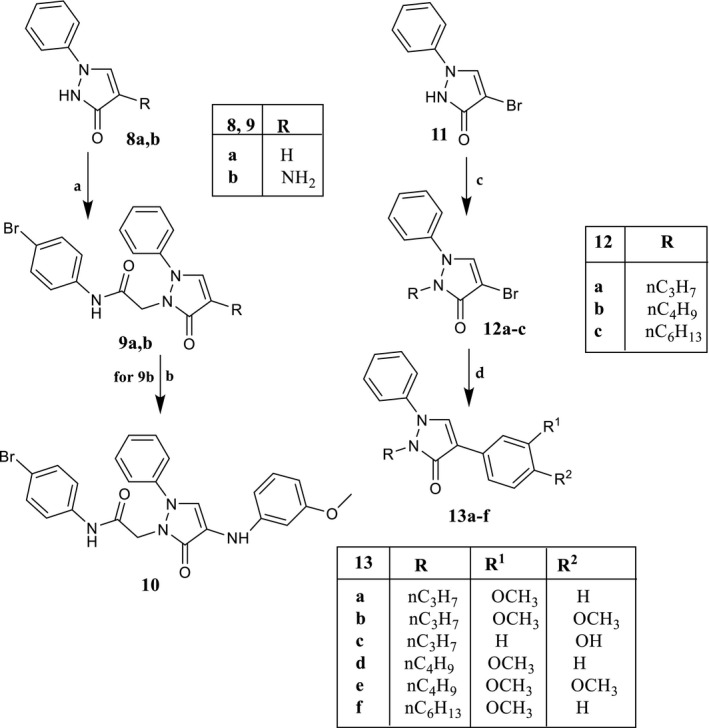
Reagents and conditions: (a) N‐(4‐bromophenyl)‐2‐chloroacetamide, K_2_CO_3_, anhydrous CH_3_CN, reflux, 2–7 hr. (b) For **9b:** 3‐methoxybenzeneboronic acid, (CH_3_COO)_2_Cu, Et3N, activated molecular sieves, anhydrous CH2Cl2, rt, 3 hr. (c) Alkyl bromide, K_2_CO_3_, anhydrous DMF, reflux, 1 hr. (d) Tetrakis(triphenylphosphine)palladium(0), arylboronic acid, Na_2_CO_3_, anhydrous toluene, rt, 15–18 hr; for compounds **13b,c** and **13e,f**, reflux, 6–12 hr

**SCHEME 4:**
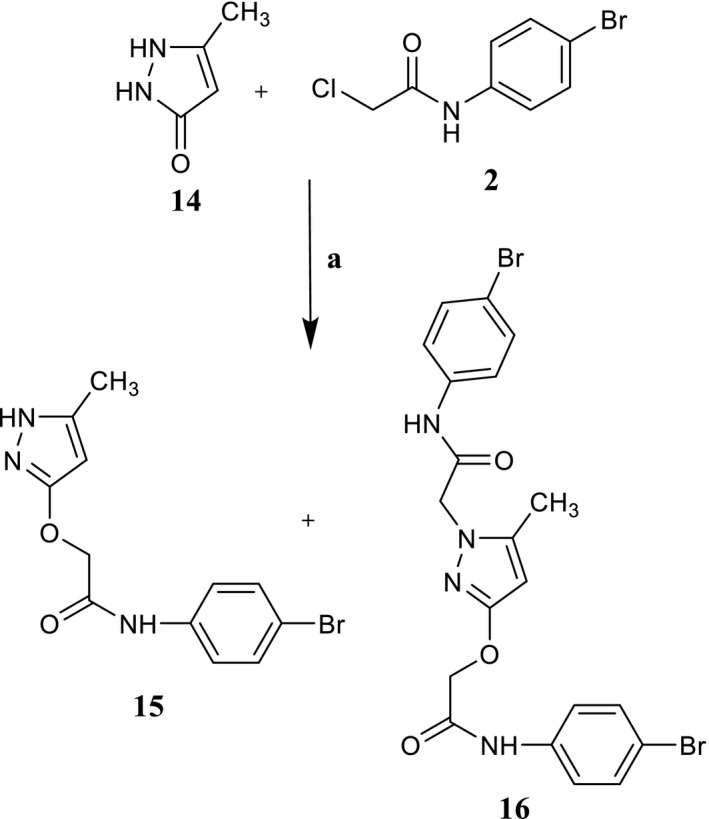
Reagents and conditions: (a) K_2_CO_3_, anhydrous CH_3_CN, reflux, 2–7 hr

All new compounds were synthesized as reported in Schemes 1, 2, 3, 4, and the structures were confirmed by analytical and spectral data. The synthetic pathways leading to the final pyrazole derivatives of type **4** and **7**, modified at positions 3, 4, and 5, are shown in Schemes 1 and 2, respectively. Compounds **4a–e** were obtained starting from the appropriate commercially available precursors **1a–c**, which were reacted with N‐(4‐bromophenyl)‐2‐chloroacetamide (**2**; Baraldi et al., 2007), in anhydrous CH_3_CN to obtain the intermediate 5‐aminopyrazoles **3a‐c**. Finally, a coupling reaction between **3a‐c** and the suitable 3‐ or 4‐methoxybenzeneboronic acid, with copper(II)acetate and Et_3_N as catalysts, resulted in the final desired compounds of type **4**.

For the synthesis of **7a,b** (Scheme 2), the previously described isoxazolo‐pyridazinones **5a,b** (Dal Piaz et al., 2004) were transformed into the corresponding 5‐methylpyrazole derivatives **6a,b** by treatment with a solution of 2.5N NaOH in EtOH and then alkylated with the fragment **2** under the same conditions as previously described.

In Scheme 3 is described the synthetic procedure for the novel pyrazolones **9a, b**, **10**, and for **13a‐f**, which lack the 4‐Br‐phenylacetamide chain at the pyrazolone N‐1 position. Alkylation of the appropriate pyrazolones **8a** and **8b** (O’Brain & Gates, 1966) with fragment **2** resulted in compound **9a** and the intermediate **9b**, which was transformed into the final compound **10** through a coupling reaction with 3‐methoxybenzeneboronic acid. For synthesis of derivatives of type **13**, which present an alkyl chain at N‐1 of pyrazolone, the starting compound **11** (O’Brain & Gates, 1966) was reacted with the suitable alkyl bromide under standard conditions, resulting in intermediates **12a‐c**, which in turn were transformed into compounds **13a‐f** through a cross‐coupling reaction with appropriate arylboronic acid, palladium(0)‐tetrakis triphenylphosphine (Tetrakis), and Na_2_CO_3_ in anhydrous toluene.

**FIGURE 3:**
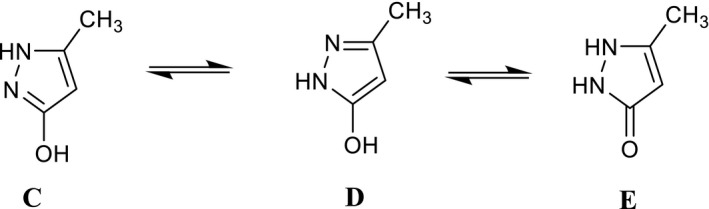
The most representative tautomer forms for pyrazol‐3‐one scaffold

Finally, when the commercially available pyrazolone **14** was reacted with **2** under the same conditions, a mixture of the *O*‐alkylated pyrazolone **15** and di‐alkylated derivative **16** was obtained (Scheme 4). Formation of the *O*‐alkylated derivative agrees with the numerous possible tautomeric forms reported by Katritzky and co‐workers for this nucleus (Katritzky & Maine, 1964), which can exist as eight different tautomers. Among them, the most representative is form **C,** followed by **D** and **E** (Arakawa et al., 1974; Figure 3).

In order to assign the correct structure to compounds **15** and **16**, we performed an extensive nuclear magnetic resonance (NMR) study using monodimensional (^1^H‐ and ^13^C‐NMR) and bidimensional (^1^H‐^13^C HSQC and ^1^H‐^13^C HMBC) NMR techniques (see Supporting Information for the spectra, Figures S1–S8). The first observation was that the chemical shift (^1^H‐NMR) of the *CH_2_* of chain **2** in compound **15** (δ = 4.62 ppm) differed from the chemical shift value of the same group in the N‐alkylated compounds of type **3**, **4**, **7**, **9**, and **10**, all exhibiting a δ value around 4.80 ppm. These data allowed us to hypothesize that in compound **15** the alkylation reaction with **2** probably results in the *O*‐substituted derivative. This hypothesis is consistent with results from the double alkylation product **16** whose ^1^H‐NMR spectrum shows two signals at δ = 4.62 ppm and δ = 4.72, both compatible with the chemical shifts of O‐*CH_2_* and N‐*CH_2_*, respectively.

Moving to analysis of the ^13^C‐NMR spectra, we found that the chemical shift value of *CH_2_* from the 4‐Br‐phenylacetamide chain in the *N*‐alkylated compounds of type **4**, **7**, and **9** was always around 52–53 ppm. The ^13^C‐NMR analysis indicated that a peak at δ = 67.57 ppm was present in compound **15**, corresponding to the *CH_2_* group, while the spectrum of compound **16** exhibited two signals at δ = 67.62 ppm and at δ = 52.06 ppm. Moreover, for compound **15**, additional 2D NMR techniques such as heteronuclear single quantum coherence (HSQC) and heteronuclear multiple bond correlation (HMBC) were used (see Supporting Information). Through HSQC, the correlation between the protons and the corresponding carbons was assigned (Cα). Using HMBC, which correlates protons with carbons in the long range, it was possible to confirm that the 4‐bromophenylacetamide chain was not bound to the N‐1, since the correlation between *CH_2_* of the chain and *C5* and C5‐*CH_3_* of the pyrazolo nucleus was missing. On the contrary, it was not possible to determine whether the 4‐bromophenylacetamide chain was at the N‐2 or at the oxygen of the heterocycle. In fact, the alkylation to nitrogen at position 2 or to oxygen at position 3 would give a similar coupling. In both spectra, it would be possible to observe a correlation between *CH_2_* and *CO* of the chain and *CH_2_* and C3 of heterocycle. Thus, this result did not allow us to distinguish the structure. These data strongly suggest a single *O*‐alkylation for compound **15** and a twofold alkylation for compound **16** (N‐1 and oxygen), both according to the most representative tautomer **C** shown in Figure 3 and to a minor steric hindrance in comparison with a double alkylation at N‐2 and O. By using single‐crystal X‐ray diffraction, we confirmed our hypothesis, as described below.

### 3.2 Solid‐state structure from single‐crystal X‐ray diffraction (SCXRD)

**FIGURE 4:**
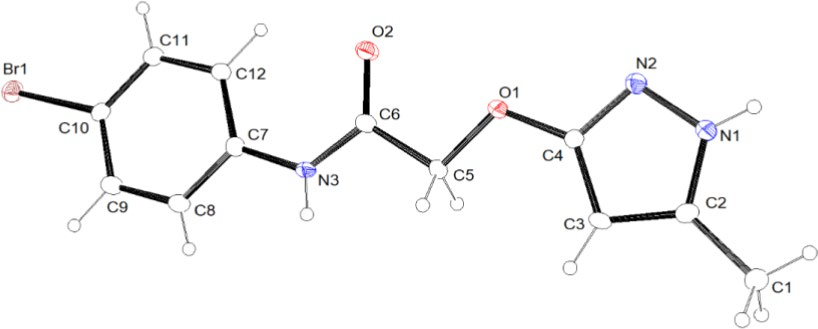
ORTEP view of the molecular structure of **15**. Ellipsoid probability = 20%

In order to univocally assign the correct structure to compound **15**, crystallographic analysis was performed, and the molecular structure of **15** is shown in Figure 4.

In the asymmetric unit, just one molecule of **15** is present. The choice of the correct tautomer of the pyrazole scaffold between the two most representative **C** and **D** forms (see Figure 3) was made on the basis of the position of the hydrogen atom bonded to the nitrogen atom N1, as observed in the difference Fourier map. A search of the Cambridge Structural Database (CSD, v. 5.41 update 3 August 2020; Groom et al., 2016) for molecules containing fragments **C** and **D** of Figure 3 identified five molecules containing tautomer **C** and two containing tautomer **D** (only organic compounds were considered). Table 1 reports the bond distances involving the atoms of the five‐membered ring in **15** and those retrieved in the CSD. The trend of the bond distances in **15**, as compared with the values found in the CSD, supports our observations reported above using SCXRD, that is, the tautomer of the pyrazole scaffold present in **15** is the **C** tautomer.

**TABLE 1:** Selected bond distances in **15** and in the 5‐membered rings of the **C** and **D** tautomers found in the CSD (see Figure 3)

Bond	Distance (Å)		
	15	Tautomer C min–max distance (mean)	Tautomer D min–max distance (mean)
**N1‐C2**	1.339(5)	1.337–1.361 (1.345)	1.312–1.321 (1.316)
**C2‐C3**	1.378(6)	1.366–1.386 (1.374)	1.333–1.392 (1.362)
**C3‐C4**	1.395(5)	1.381–1.399 (1.393)	1.350–1.377 (1.363)
**N2‐C4**	1.320(5)	1.323–1.335 (1.328)	1.330–1.343 (1.336)
**N2‐N1**	1.365(5)	1.354–1.374 (1.366)	1.341–1.355 (1.348)

**FIGURE 5:**
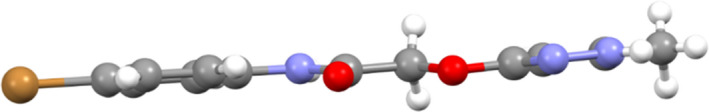
Side view of **15**

The overall shape of the molecule is definitely planar (see Figure 5), with the two rings forming an angle of 3.5 (1)°. Considering the mean plane defined by all of the non‐hydrogen atoms of the molecule, the maximum deviation from planarity is due to C12 (0.119(4) Å).

Finally, each molecule is involved in four strong intermolecular H‐bonds: 2 as donor (with N1 and N3) and 2 as acceptor (with O2 and N2). Details are given in the Supporting Information (Tables S1 and S2 and Figure S10).

### 3.3 Biological results

All new compounds were evaluated for their ability to induce intracellular Ca^2+^ flux in human neutrophils (hPMN) and in human HL‐60 cells transfected with FPR1 and FPR2, and the results are reported as EC_50_ values in Tables 2 and 3 using as reference compounds fMLF (FPR1 agonist), WKYMVm (FPR2 agonist), and the previously described agonists **EC3** and **EC10** (Vergelli et al., 2016, 2017). All compounds were also evaluated in wild‐type non‐transfected HL‐60 cells and were found to be inactive.

**TABLE 2:** Effect of compounds **4a‐e**, **7a,b**, and **15** on Ca^2+^ mobilization in FPR‐transfected HL60 cells and human neutrophils (hPMN)

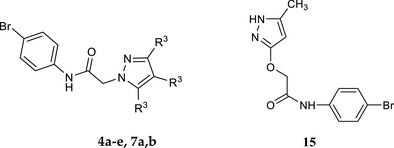						
Compd.	R^3^	R^4^	R^5^	EC_50_ (µM) and efficacy (%)^a^		
				HL60‐FPR1	HL60‐FPR2	hPMN
**4a**	H	CN	3‐OCH_3_PhNH	N.A.^b^	N.A.^b^	N.A.^b^
**4b**	H	CN	4‐OCH_3_PhNH	N.A.^b^	N.A.^b^	N.A.^b^
**4c**	Ph	CH_3_	3‐OCH_3_PhNH	N.A.^b^	N.A.^b^	N.A.^b^
**4d**	Ph	CH_3_	4‐OCH_3_PhNH	N.A.^b^	N.A.^b^	N.A.^b^
**4e**	H	H	3‐OCH_3_PhNH	13.2 ± 2.6 (85)	23.4 ± 5.3 (60)	8.2 ± 2.6 (75)
**7a**	3‐OCH_3_Ph	CN	CH_3_	18.4 ± 4.3 (75)	6.1 ± 2.2 (75)	6.5 ± 1.6 (80)
**7b**	4‐OCH_3_Ph	CN	CH_3_	N.A.^b^	N.A.^b^	N.A.^b^
**15**	_	_	_	9.8 ± 2.7 (60)	25.9 ± 6.4 (60)	18.6 ± 4.7 (55)
***f*MLF**				0.01		
**WKYMVm**					0.001	
**EC3**				0.019 ± 0.005 (85)	0.043 ± 0.0016 (80)	0.006 ± 0.002 (150)
**EC10**				0.045 ± 0.016 (185)	0.170 ± 0.038 (60)	0.036 ± 0.007 (150)

^a^ EC_50_ values represent the mean of three independent experiments and were determined by nonlinear regression analysis of the concentration–response curves (5–6 points) generated using GraphPad Prism 5 with 95% confidential interval (*p* < .05). Efficacy is expressed as % of the response induced by 5 nM fMLF (FPR1) and 5 nM WKYMVm (FPR2).

^b^ N.A., no activity (no response was observed during first 2 min after addition of compounds under investigation) considering the limits of efficacy <20% and EC_50_ < 50 µM.

**TABLE 3:** Effect of compounds **9b**, **10,** and **13a‐f** on Ca^2+^ mobilization in FPR‐transfected HL60 cells and human neutrophils

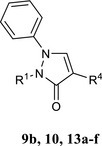					
Compd.	R^1^	R^4^	EC_50_ (µM) and efficacy (%)^a^		
			HL60‐FPR1	HL60‐FPR2	hPMN
**9b**	p‐Br‐Ph‐NHCOCH_2_	H	N.A.^b^	23.1 ± 6.3 (50)	N.A.^b^
**10**	p‐Br‐Ph‐NHCOCH_2_	3‐OCH_3_PhNH	N.A.^b^	N.A.^b^	N.A.^b^
**13a**	C_3_H_7_	3‐OCH_3_Ph	N.A.^b^	N.A.^b^	N.A.^b^
**13b**	C_3_H_7_	3,4‐(OCH_3_)_2_Ph	N.A.^b^	N.A.^b^	N.A.^b^
**13c**	C_3_H_7_	4‐OHPh	N.A.^b^	N.A.^b^	N.A ^b^
**13d**	C_4_H_9_	3‐OCH_3_Ph	N.A.^b^	N.A.^b^	N.A.^b^
**13e**	C_4_H_9_	3,4‐(OCH_3_)_2_Ph	N.A.^b^	N.A.^b^	N.A.^b^
**13f**	C_6_H_13_	3‐OCH_3_Ph	20.2 ± 5.4 (35)	10.5 ± 3.5 (90)	13.8 ± 4.2 (55)
***f*MLF**			0.01		
**WKYMVm**				0.001	
**EC3**			0.019 ± 0.005 (85)	0.043 ± 0.0016 (80)	0.036 ± 0.007 (150)
**EC10**			0.04 5 ± 0.016 (185)	0.170 ± 0.038 (60)	0.036 ± 0.007 (150)

^a^ EC_50_ values represent the mean of three independent experiments and were determined by nonlinear regression analysis of the concentration–response curves (5–6 points) generated using GraphPad Prism 5 with 95% confidential interval (*p* < .05). Efficacy is expressed as % of the response induced by 5 nM fMLF (FPR1) and 5 nM WKYMVm (FPR2).

^b^ N.A., no activity (no response was observed during first 2 min after addition of compounds under investigation) considering the limits of efficacy <20% and EC_50_ < 50 µM.

The new pyrazole and pyrazolone derivatives were tested both on human HL‐60 cells transfected with FPR1 or FPR2 and on PMNs expressing FPR1 and FPR2 in order to understand whether the effect on calcium flux is due exclusively to the interaction with the FPR system or whether a different non‐specific mechanism can coexist.

Looking at the biological results, we can observe that only a few compounds show some activity, which is in any case very modest.

In particular, among the pyrazoles of types **4** and **7**, all containing the 4‐bromophenylacetamide chain at N‐1, and the *O*‐alkylated derivative **15** (Table 2), only **4e**, **7a**, and **15** showed moderate FPR agonist activity in the micromolar range (**4e**: EC_50_ = 13.2 µM for FPR1, 23.4 µM for FPR2; **7a**: EC_50_ = 18.4 µM for FPR1, and 6.1 µM for FPR2; **15**: EC_50_ = 9.8 µM for FPR1 and 25.9 µM for FPR2). Compound **7a** had a threefold preference for FPR2, while **15** displays a similar selectivity for FPR1.

In the pyrazolone series, only compound **9b**, bearing the 4‐bromophenylacetamide chain at N‐2, showed moderate, but selective activity for FPR2 (EC_50_ = 23.1 µM; Table 3), while among the pyrazolones lacking the 4‐bromophenylacetamide chain, only compound **13f**, the N‐2 hexyl derivative, had slight non‐selective activity (EC_50_ = 20.2 µM for FPR1 and EC_50_ = 10.5 µM for FPR2), Table 3.

As regard the evaluation on hPMN, with the exception of compound **9b** which is inactive, all the other compounds exhibit a moderate activity in the micromolar range, comparable with the values reported for the tests on HL‐60 cells transfected with FPR1 or FPR2, thus indicating that the increase in intracellular calcium is due to activation of the FPR system.

The low or absent activity of these new compounds suggests that the pyrazole nucleus was not appropriate for the synthesis of FPR agonists. We can further speculate that this five‐member scaffold could be responsible for a worse arrangement in the receptor binding site, as the same substituents (the 4‐bromophenyl acetamide chain or methoxyphenyl group) previously inserted into the six‐member pyridazinone and pyridinone scaffolds resulted in very potent FPR agonists. To address this issue, molecular docking studies were performed.

### 3.4 Molecular docking

In order to evaluate potential differences in interaction with FPRs of highly active pyridazinones **EC10** (Vergelli et al., 2016) and **EC3** (Vergelli et al., 2017) and pyridinone **2a** (Crocetti et al., 2020) on the one hand, and moderately active or inactive newly synthesized compounds containing the pyrazole or pyrazolone scaffolds on the other hand, we evaluated docking of **EC3**, **EC10**, **2a**, **4e**, **7a**, **10**, and **15** into the FPR1 and FPR2 binding sites. The FPR1 and FPR2 geometries reported by Zhuang et al., 2020 were used as sources of the receptor structures for docking.

**FIGURE 6:**
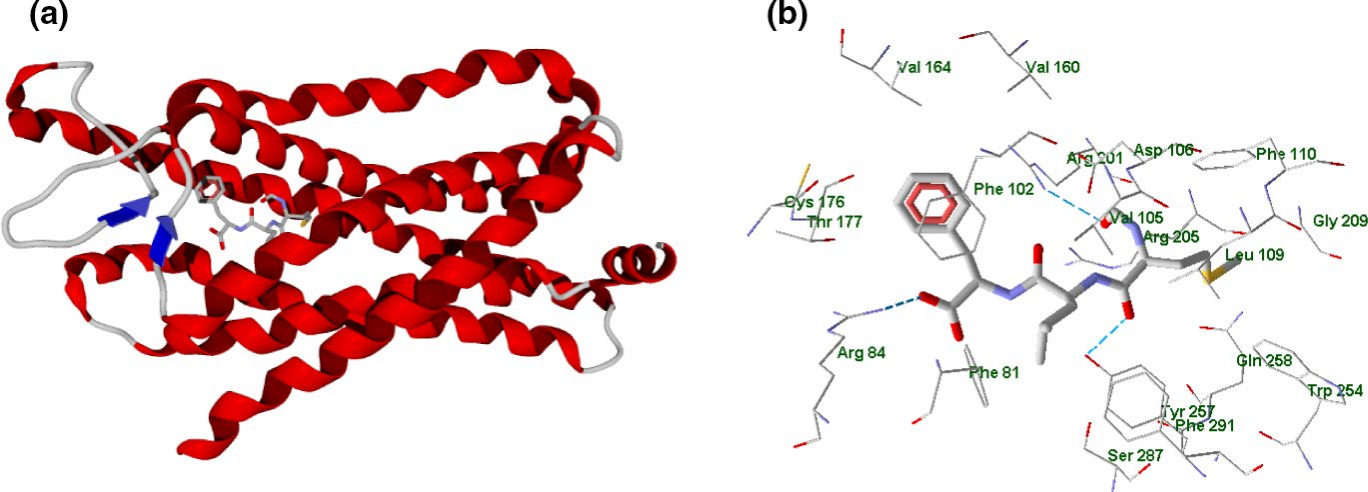
fMLF peptide docked in FPR1 homology model (Zhuang et al., 2020; panel a). Image of the fMLF peptide (stick representation) docked in FPR1 homology model. Residues within 3 Å from the peptide are shown (panel b)

The general view of the FPR1 homology model with docked fMLF (Zhuang et al., 2020) is shown in Figure 6 (secondary structure panel a). Furthermore, Figure 6 panel b presents the fMLF molecule surrounded by residues within 3 Å, which can be regarded as the residues of the FPR1 binding site. The image of fMLF (stick representation) docked into the FPR1 homology model was built with the MVD program from a PDB file of the FPR1‐fMLF complex obtained from the authors of paper (Zhuang et al., 2020).

According to the analysis made with MVD software, the fMLF peptide forms H‐bonds with Arg84, Arg201, and Tyr257 residues. Additionally, we have analyzed partial docking scores (PDS) for each residue using MolDock scoring functions. The top residues sorted by attractive interactions according to PDS are given in Table S3 reported in Supporting Information.

The non‐HB interactions are mostly van der Waals in nature. The strongest interaction with the participation of Phe102 residue can be due to π,π‐stacking between the aromatic rings. The other strong interaction of fMLF with Arg84 is caused by an attraction between charged guanidine and carboxylic moieties in the peptide and ligand, respectively (Figure 6, panel b).

**FIGURE 7:**
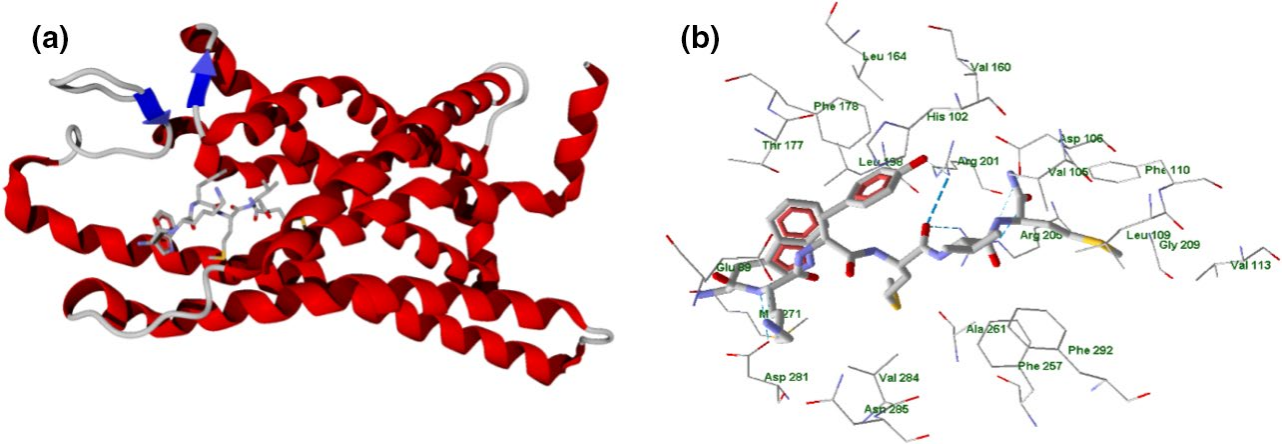
WKYMVm peptide complexed with Chain R of FPR2 receptor (secondary structure; Zhuang et al., 2020; panel a). Residues within 3 Å from the peptide are shown (panel b)

The experimentally obtained structure of FPR2 complexed with WKYMVm (PDB 6OMM; Zhuang et al., 2020) contains five protein chains (Figure 7). As shown in Figure 7, WKYMVm (stick representation) is complexed with Chain R of FPR2 (secondary structure view, Figure 7 panel a; PDB 6OMM; Zhuang et al., 2020).

Residues within 3 Å of the peptide are shown in Figure 7 panel b, where the peptide forms H‐bonds with Arg201, Arg205, Asp281, Glu89 (weak), and Asp106 (weak). The strongest interaction with the ligand, according to the PDS analysis (Table S5 in Supporting Information) corresponds to Arg205 of FPR2, as this residue forms three H‐bonds with different atoms of WKYMVm (Figure 7, panel b). In addition, the charged carboxyl groups of Glu89 and Asp281 electrostatically interact with the protonated amine group and N‐H bond of the WKYMVm lysine residue, which leads to high PDS values for Glu89 and Asp281 (Table S5).

**FIGURE 8:**
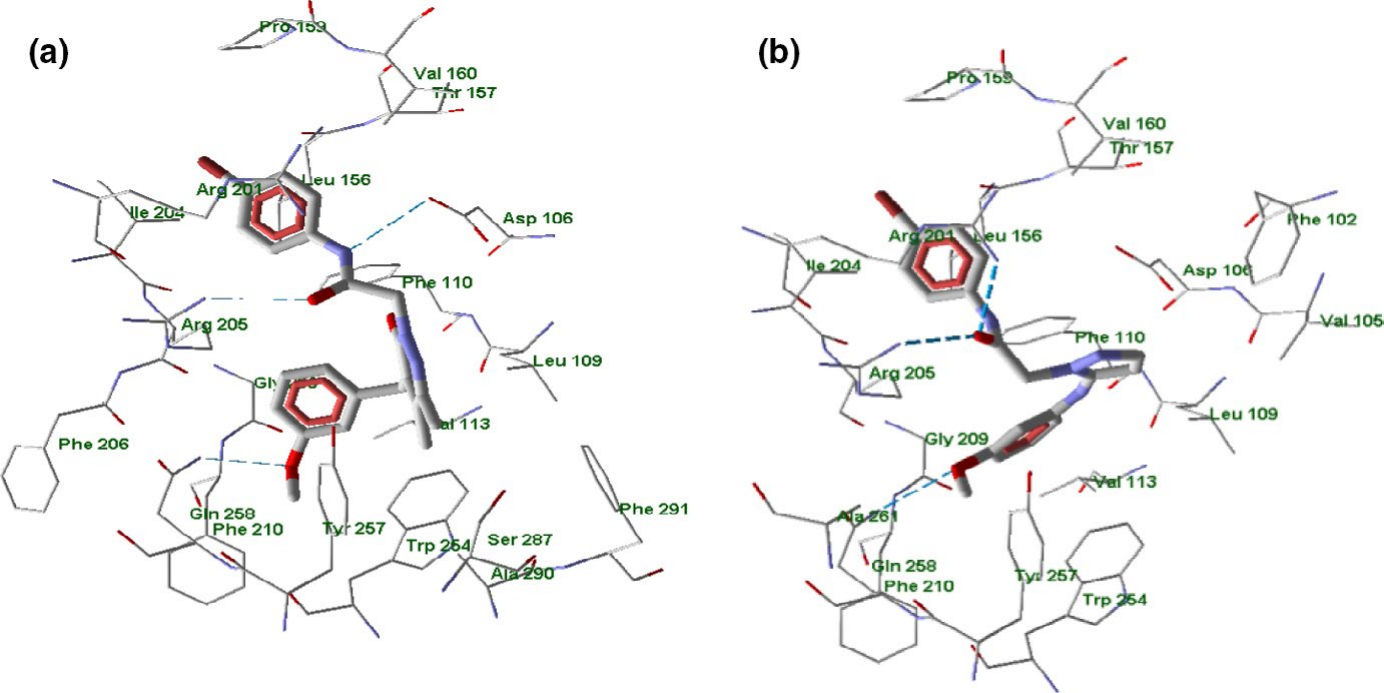
Docking poses of compounds **EC3** (panel a) and **4e** (panel b) in FPR1 binding site. Residues within 4 Å from each pose are shown

According to our docking results, the compounds form H‐bonds with Asp106 (**EC3**), Arg201 (**EC10**, **2a**, **4e**, **7a**, **10**, **15**), Arg205 (**EC3**, **4e**, **7a**, **10**), Gln258 (**EC3**, **2a**, **4e**, **10**), or Ser287 (**2a**) upon their binding with FPR1 (see examples in Figure 8 and Figures S11–S16 in Supporting Information).

In particular, **EC3** (Vergelli et al., 2017) has H‐bond interactions via the oxygen of the 3‐methoxyphenyl group and the acetamido carbonyl oxygen with Gln258 and Arg205, respectively. The NH of the same acetamido chain interacts with Asp106, Figure 8 panel a. Compound **4e**, taken as a representative example of the new derivatives, shows a three‐centered H‐bond interaction with Arg201 and Arg205 via the oxygen carbonyl of the acetamido chain; the oxygen atom of the 3‐methoxyphenyl moiety interacts with Gln258, Figure 8 panel b.

**FIGURE 9:**
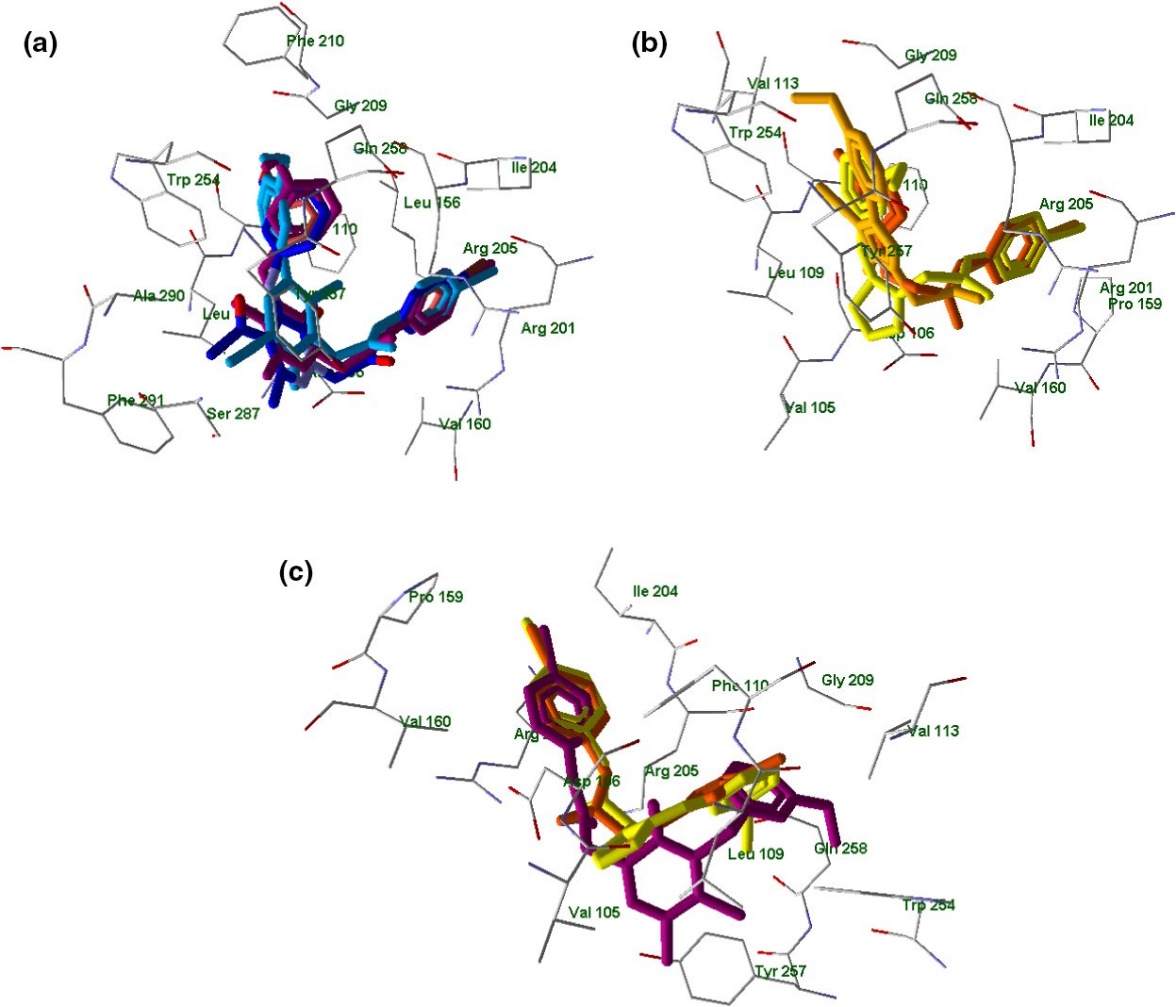
Superimposed docking poses of selected compounds in FPR1. Panel a: superimposed docking poses of **EC3** (violet), **EC10** (dark‐blue), and **2a** (light‐blue). Residues within 3 Å of the **EC3** pose are visible. Panel b: superimposed docking poses of **4e** (light‐yellow), **7a** (dark‐yellow), and **15** (orange). Residues within 3 Å of the **4e** pose are visible. Panel c: superimposed docking poses of **EC3** (violet), **4e** (light‐yellow), and **15** (orange). Residues within 3 Å of the **4e** pose are visible

In this regard, there were no significant differences in hydrogen bonding patterns between highly potent and moderately active FPR agonists. Nevertheless, positioning of FPR1 agonists containing six‐membered and five‐membered heterocycles visibly differed (Figure 9, panels a–c).

The 4‐bromophenyl substituents of all investigated molecules (**EC3**, **EC10**, **2a**, Figure 9, panel a; **4e**, **7a, 15**, Figure 9, panel b) in their docking poses are located in the same area of space in the vicinity of Leu156, Arg201, and Ile204. In addition, the 3‐methoxyphenyl groups of **EC3**, **EC10**, **2a** (panel a) and **4e**, **7a**, **15** (panel b) occupy a pocket between Gly209, Trp254, and Gln258. However, six‐membered pyridazinone and pyridinone moieties of potent FPR1 agonists **EC3**, **EC10**, and **2a** occupy a quite different area of space (close to Tyr257, Ser287, and Phe291) than pyrazole heterocycles of moderately active compounds **4e**, **7a**, and **15**, which could explain their reduced activity. In Figure 9 panel c is reported the superimposition of **EC3** with new compounds **4e** and **15** where it is possible to observe this situation.

**FIGURE 10:**
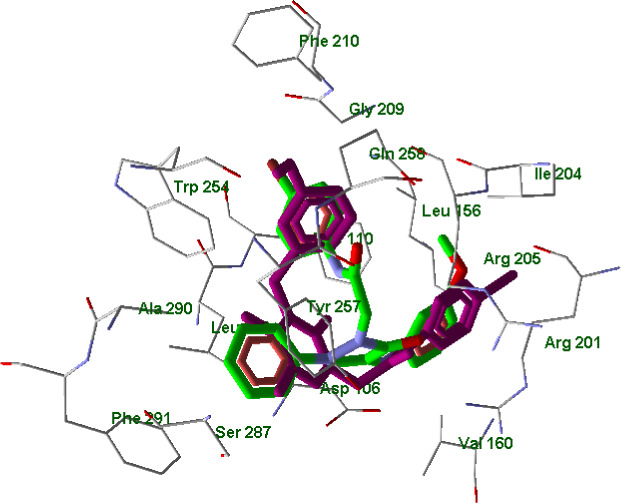
Superimposed docking poses of compounds **EC3** (violet) and **10** (green skeleton) in FPR1 binding site. Residues within 3 Å from **EC3** pose are visible

The inactive compound **10** in its best docking pose had a reverse orientation in the binding site as compared to the FPR1 agonists. Thus, the 3‐methoxyphenyl group of **10** (green) is overlaid on the 4‐bromophenyl moieties of the agonists (violet) and *vice versa* (Figure 10). Moreover, the hydrophobic phenyl substituent on the pyrazole ring of pyrazolone **10** protrudes into the pocket occupied by the polar acetyl and cyano substituents of active agonists **EC10** and **2a**, respectively (see also Figure 9, panel a).

**FIGURE 11:**
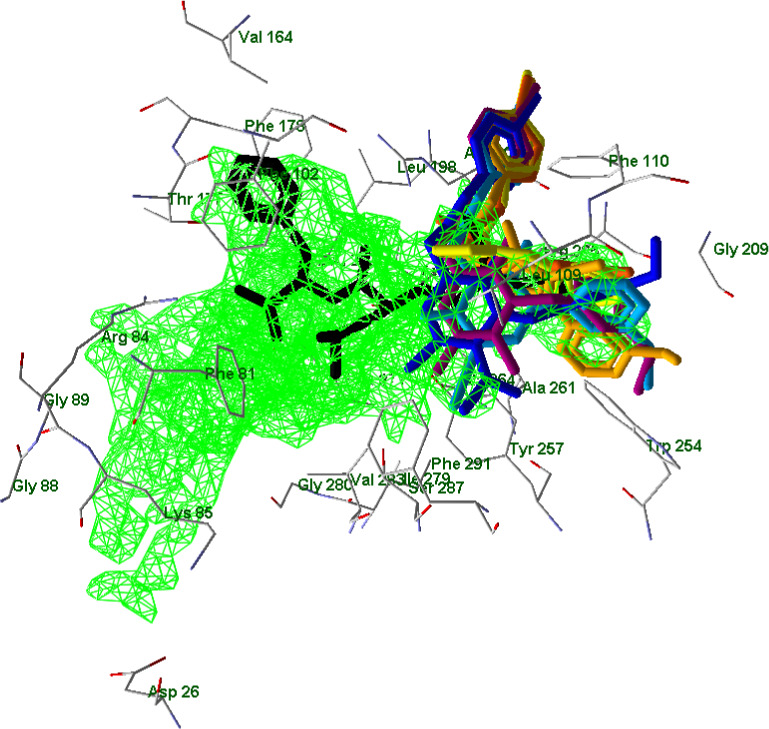
Superimposed docking poses of compounds **EC3**, **EC10**, **2a**, **4e**, **7a**, and **15** (colors as indicated before) together with a pose of fMLF peptide (black; Zhuang et al., 2020). A cavity of 663 Å^3^ in volume is shown in green grid (built by MVD software with probe size of 1.2 Å). Residues within 2.7 Å from the cavity are visible

An in‐depth molecular docking analysis taking into account the potent and selective FPR1 agonist fMLF was also performed in order to better understand the specific orientations of ligands with respect to FPR1. In Figure 11, FPR1 is shown with a 663 Å^3^ cavity that was built by the MVD program using a default probe size of 1.2 Å.

**FIGURE 12:**
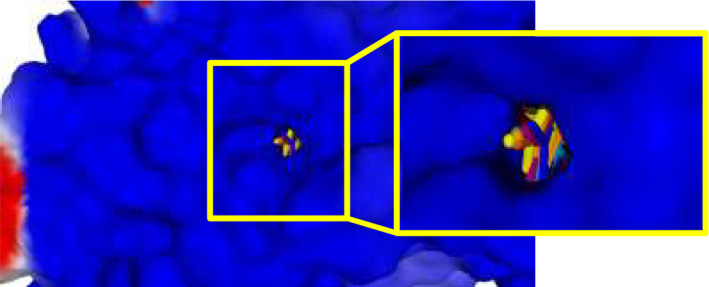
A surface of FPR1 is shown together with the poses. Ordinary and magnified views of the same surface from the front and rear (opposite) sides of the receptor are shown where the hole is clearly visible (rear side)

The heterocyclic moieties of potent agonists **EC3**, **EC10**, and **2a** occupy a sub‐pocket of the FPR1 binding site located between Tyr257 and Phe291, which is not occupied by low‐active molecules **4e**, **7a**, and **15**. Correspondingly, compounds **EC3**, **EC10**, and **2a** have relatively high PDS values with respect to Tyr257 and Phe291 (see Table S4 in Supporting Information). Inactive compound **10** also interacts with Tyr257 and Phe291; however, it has a reverse orientation in the binding site. It should be noted that *p*‐bromo substituted benzene rings of potent and moderately active FPR1 agonists occupy a branch of the cavity protruding in the vicinity of Arg201 and Arg205. This branch corresponds to a “hole” in the receptor, which is quite narrow and was not included into the cavity with the default probe size. A surface of FPR1 is shown together with these poses (Figure 12).

**FIGURE 13:**
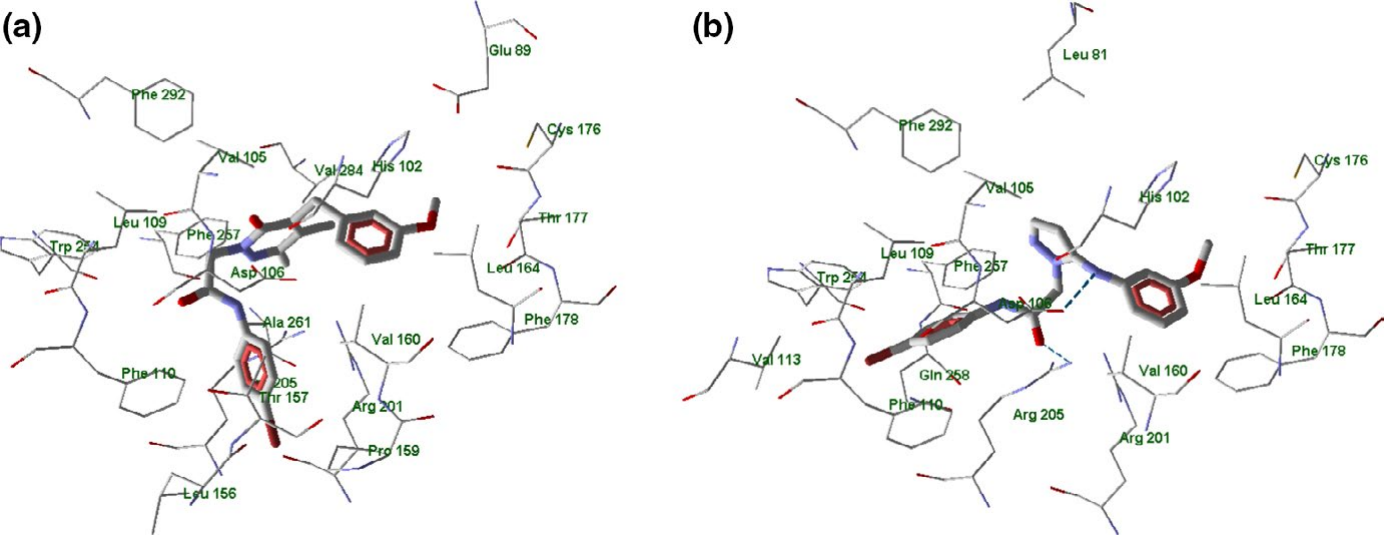
Docking poses of compounds **EC3** (panel a) and **4e** (panel b) in FPR2 binding site. Residues within 4 Å from each pose are shown

Docking of the compounds into the FPR2 binding site led to docking poses in which the molecules were H‐bonded to Asp106 (**2a**—weak H‐bond, **4e**), Arg201 (**EC10**—weak H‐bond, **7a**), Arg205 (**EC10**, **7a**, **15**—weak H‐bonds, **4e**), and Ser288 (**10**; see examples in Figure 13 and Figure S17–S22 in Supporting Information).

When compound **4e** has docked into the FPR2 binding site (PDB 6OMM; Zhuang et al., 2020), the H‐bonds with Asp106 and Arg205 were evident and the 4‐bromophenylacetamido substituent was oriented toward Leu109 and Phe110, Figure 13 panel b. It should be noted that agonist **EC3** does not form H‐bonds with FPR2. However, according to the docking scores, compound **EC3** has significant attractive van der Waals interactions with Arg201, Arg205, His106, and Asp106 of FPR2, which are likely to be responsible for FPR agonist activity Figure 13 panels a‐b.

**FIGURE 14:**
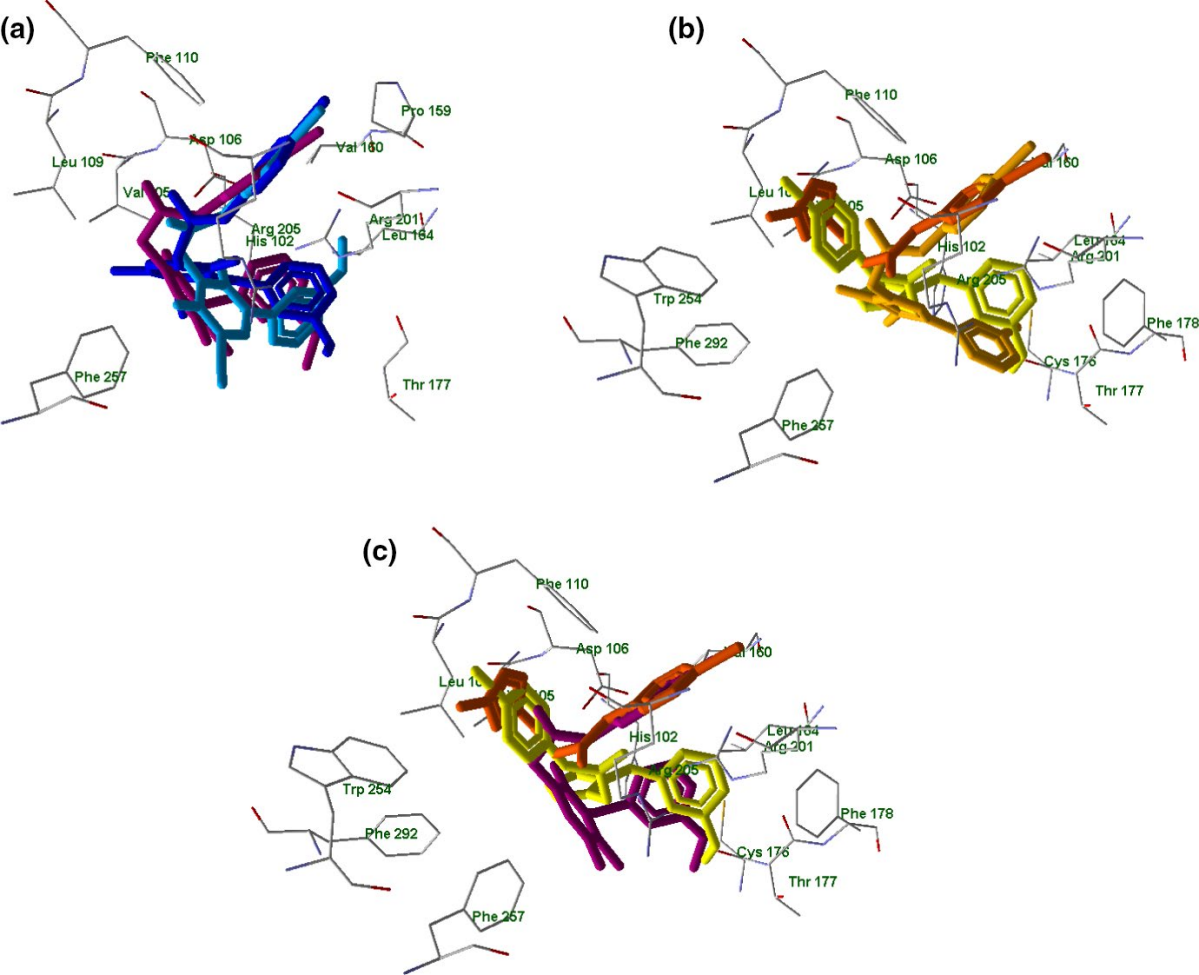
Superimposed docking poses of selected compounds in FPR2. Panel a: superimposed docking poses of compounds **EC3** (violet), **EC10** (dark‐blue), **2a** (light‐blue). Residues within 3 Å of the **EC3** pose are visible. Panel b: superimposed docking poses of compounds **4e** (light‐yellow), **7a** (dark‐yellow), and **15** (orange). Residues within 3 Å of the **4e** pose are visible. Panel c: superimposed docking poses of **EC3** (violet), **4e** (light‐yellow), and **15** (orange) in FPR2. Residues within 3 Å of the **4e** pose are visible

The superimposed docking poses of potent and moderately active agonists onto the FPR2 binding site are shown in Figure 14 panel a–c. The highly active agonists **EC3**, **EC10**, and **2a** containing pyridazinone and pyridinone moieties are positioned similarly to each other within the binding site: their 4‐bromophenyl and 3‐methoxyphenyl fragments are correspondingly overlaid (Figure 14, panel a). For the pyrazole‐containing compound **4e**, the 4‐bromophenyl substituent is oriented differently and directed toward Leu109 and Phe110. The docking pose of compound **7a** is very similar to the poses found for agonists **EC3**, **EC10**, and **2a** (Figure 14, panel b). This observation agrees with the relatively high FPR2 agonist activity of **7a** compared with other the pyrazole derivatives evaluated. While the substituted pyrazole **15** in its best docking pose had its 4‐bromophenyl moiety nearly overlaid with that of potent agonists, the methylpyrazole group of **15** protruded toward Leu109 and Phe110 (Figure 14, panels b and c).

**FIGURE 15:**
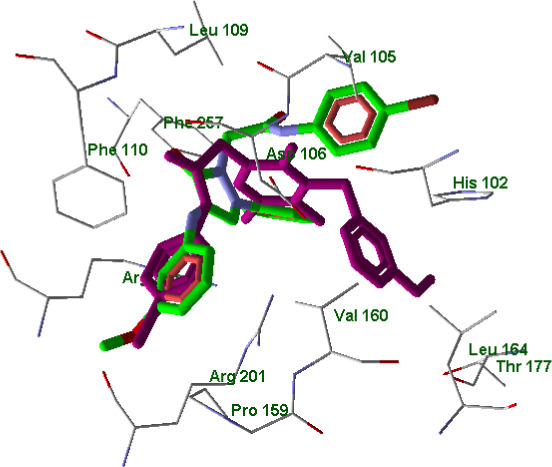
Superimposed docking poses of compounds **EC3** (violet) and **10** (green skeleton) in FPR2 binding site (PDB code 6OMM). Residues within 3 Å from **EC3** pose are visible

As for inactive pyrazolone **10**, its docking pose also has an altered orientation within the FPR2 binding site (Figure 15) with respect to the poses of the potent agonists.

**FIGURE 16:**
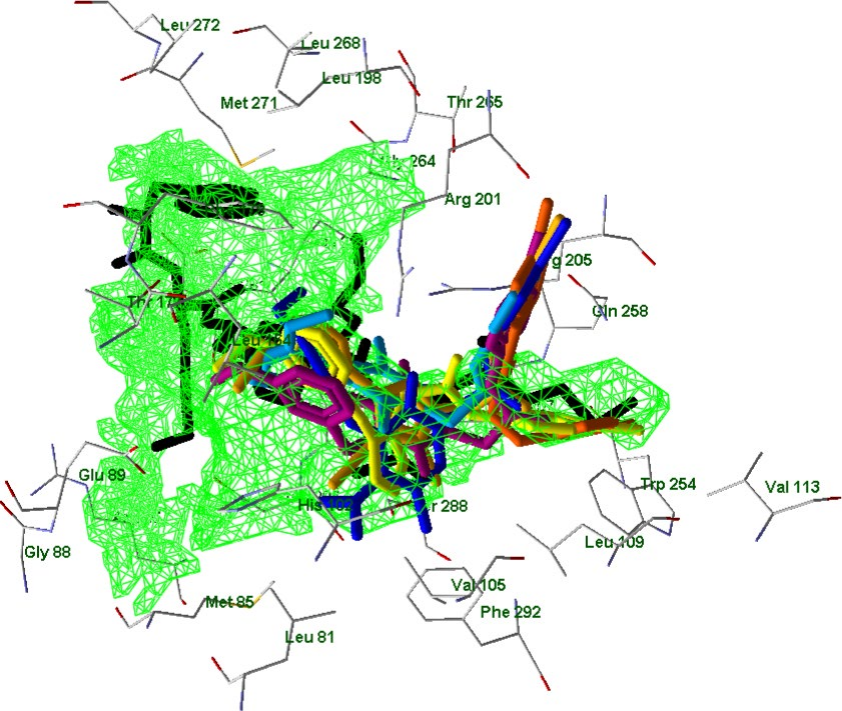
Superimposed docking poses of compounds **EC3**, **EC10**, **2a**, **4e**, **7a**, and **15** (colors as indicated before) together with an experimentally obtained conformation of WKYMVm (black; PDB 6OMM). A cavity of 628 Å^3^ in volume is shown in green grid (built by MVD software with probe size of 1.2 Å)

Regarding FPR2, its binding site is represented by a cavity of 628 Å^3^ in volume, and the related figures are presented similarly to the corresponding FPR1 model. Chain R of the protein is shown (notation according to structure 6OMM from the PDB), while the other protein chains are hidden. WKYMVm is shown in black (Figure 16).

**FIGURE 17:**
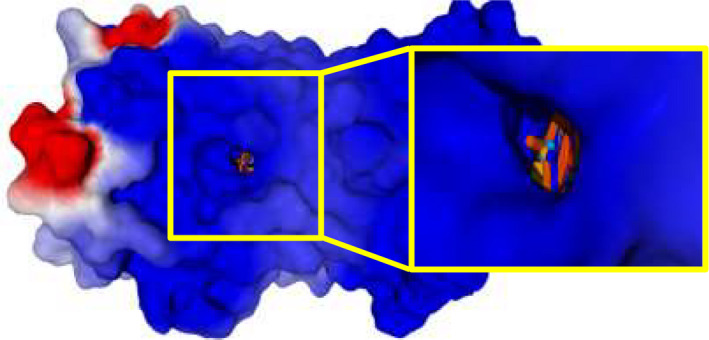
A surface of FPR2 receptor is shown together with the poses. Ordinary and magnified view of the same surface from the front and rear (opposite) sides of the receptor are shown where the hole is clearly visible (rear side). WKYMVm peptide is shown in black

Again, *p*‐bromo substituted benzene rings of potent and moderately active FPR2 agonists are pulled into the lateral hole of the binding site near Arg201 and Arg205 residues (except compound **4e** – shown in light‐yellow) as reported in Figure 17.

## 4 CONCLUSION

In conclusion, we synthesized a new series of pyrazole and pyrazolone derivatives as potential FPRs agonists. However, most of the new compounds had a low or absent FPR agonist activity, suggesting that the pyrazole scaffold was not appropriate for the synthesis of new FPRs ligands. Based on these results, we can say that our assumptions of probable different arrangements in the FPR1 and FPR2 binding site of compounds with a six‐membered core like the pyridazinones/pyridinones versus a five‐membered‐core like the pyrazoles/pyrazolones have been confirmed. In particular, comparing binding of compounds **EC3**, **EC10**, and **2a** (six‐membered core, high activity) with the new compounds **4e**, **7a**, and **15** (moderate/low activity) into the FPR1 binding site, it is possible to see that the two fragments essential for activity (4‐bromophenyl acetamide chain and 3‐methoxyphenyl group), occupy an area delimited by the same amino acids (Leu156, Arg201 and Ile204, and Gly209, Trp254, and Gln258). Moreover, the *p*‐bromo substituted benzene rings of the potent or moderately active FPR1 agonists occupy a branch of the cavity similar to a “hole” in the receptor protruding into the vicinity of Arg201 and Arg205. The only difference between the two types of scaffolds is a worse arrangement in the binding site of the five‐membered ring compared with the active six‐membered scaffolds, which could satisfactorily explain the lower FPR1 agonist activity of the pyrazoles and pyrazolones studied here. In contrast, inactive compound **10** orients the 3‐methoxyphenyl group and the 4‐bromophenylacetamide chain oppositely in the binding site with respect to the pyridazinone **EC3,** explaining/justifying its complete inactivity. We can conclude that the presence of H‐bonds is not fundamental for ligand activity in FPR2 and that probably other types of interactions could take place. Even in this case, the worse arrangement of the five‐membered core in the binding site compared with the previous series could be the main reason for the low activity or inactivity of these new pyrazole derivatives.

## 5 SUPPORTING INFORMATION

The following are available online, NMR spectra of compounds **4a**, **4c**, **7a**, **7b**, **10**, **13a**, **13d**, **13f**, **15** (Figures S1–S9); selected view of the crystal packing showing molecules of **15** interacting *via* hydrogen bonds (Figure S10); selected H‐bonds in **15** (Table S1) and crystallographic data and refinement parameters for compound **15** (Table S2); docking poses of **EC10**, **2a**, **7a**, **10,** and **15** in FPR1 and FPR2 binding site and superimposed docking poses (Figures S11–S22); analysis of partial docking scores (PDS) using MolDock scoring functions for FPR1 (Tables S3 and S4) and FPR2 (Tables S5 and S6).

## CONFLICT OF INTEREST

The authors declare no conflict of interest.

## AUTHOR CONTRIBUTIONS

L. Crocetti, M.P. Giovannoni, and C. Vergelli designed the compounds and wrote the manuscript; N. Cantini, G. Guerrini, and A. Cilibrizzi synthesized the compounds and checked the final version of the manuscript. In vitro studies and pharmacological section were performed by L.N. Kirpotina, I.A. Schepetkin, and M.T. Quinn. Molecular docking was performed by A.I. Khlebnikov. Crystallographic analysis and related section were performed by P. Rossi and P. Paoli. All authors participated in revisions and have given approval of the final manuscript version.

## Supporting information

Supinfo S1
