# Supplementary material for: Synthesis, biological evaluation, molecular modeling, and structural analysis of new pyrazole and pyrazolone derivatives as N‐formyl peptide receptors agonists
Source: Chem Biol Drug Des. 2021 Jul 1;98(4):582–603. doi: 10.1111/cbdd.13913 (PMC8446315; doi:10.1111/cbdd.13913)
Supplement: Supplementary file 1 — Supinfo S1 [file CBDD-98-582-s001.docx]

**Supporting Information**

**for**

**Synthesis, biological evaluation, molecular modeling, and structural analysis of new pyrazole and pyrazolone derivatives as N-formyl peptide receptors (FPRs) agonists**

Claudia Vergelli^1^, Andrei I. Khlebnikov^2^, Letizia Crocetti^1*^, Gabriella Guerrini^1^, Niccolò Cantini^1^, Liliya N. Kirpotina^3^, Igor A. Schepetkin^3^, Agostino Cilibrizzi^4^, Mark T. Quinn^3^, Patrizia Rossi^5^, Paola Paoli^5^ and Maria Paola Giovannoni^1^

^1^Neurofarba, Pharmaceutical and Nutraceutical Section, University of Florence, 50019 Sesto Fiorentino, Italy.

^2^National Research Tomsk Polytechnic University, Tomsk 634050, Russia.

^3^Department of Microbiology and Immunology, Montana State University, Bozeman, Montana.

^4^Institute of Pharmaceutical Science, King’s College London, Stamford Street, London SE1 9NH, UK.

^5^Department of Industrial Engineering, University of Florence, Florence, Italy.

*To whom correspondence should be addressed

Letizia Crocetti

Dept. Neurofarba, Pharmaceutical and Nutraceutical Section

Via Ugo Schiff 6

Sesto Fiorentino 50019 Firenze

Tel +39-055-4573683

E-mail: [letizia.crocetti@unifi.it](mailto:letizia.crocetti@unifi.it)

**Table of contents:**

1. NMR spectra of compounds **4a**, **4c**, **7a**, **7b**, **10**, **13a**, **13d**, **13f**, **15** (**Figures S1-S9**).
2. Selected view of the crystal packing showing molecules of **15** interacting *via* hydrogen bonds (**Figure S10**).
3. Selected H-bonds in **15** (**Table S1**) and crystallographic data and refinement parameters for compound **15** (**Table S2**).
4. Docking poses of **EC10**, **2a**, **7a**, **10** and **15** in FPR1 and FPR2 binding site and superimposed docking poses (**Figures S11-S22**).
5. Analysis of partial docking scores (PDS) using MolDock scoring functions for FPR1 (**Table S3**,**S4**) and FPR2 (**Table S5**,**S6**).

**Figure S1:** ^1^H-NMR and ^13^C-NMR of compound **4a**.


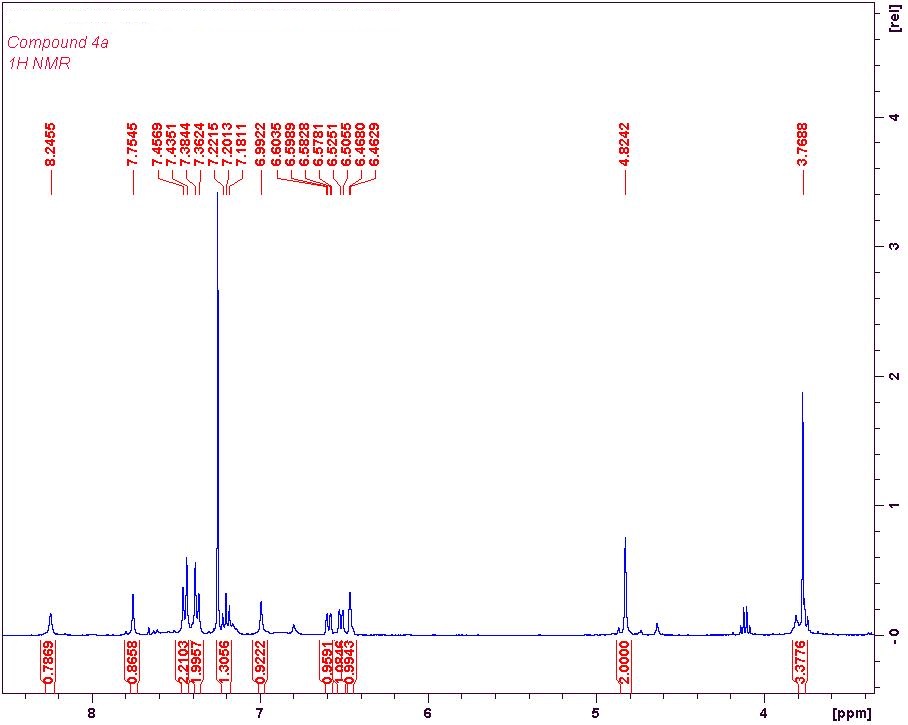


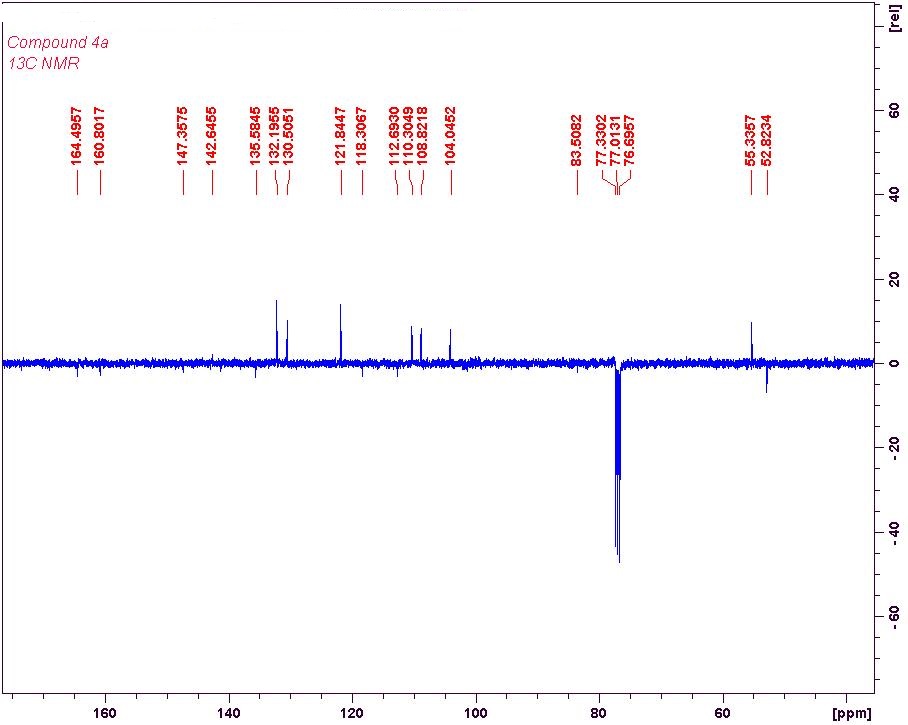


**Figure S2:** ^1^H-NMR and ^13^C-NMR of compound **4c**.


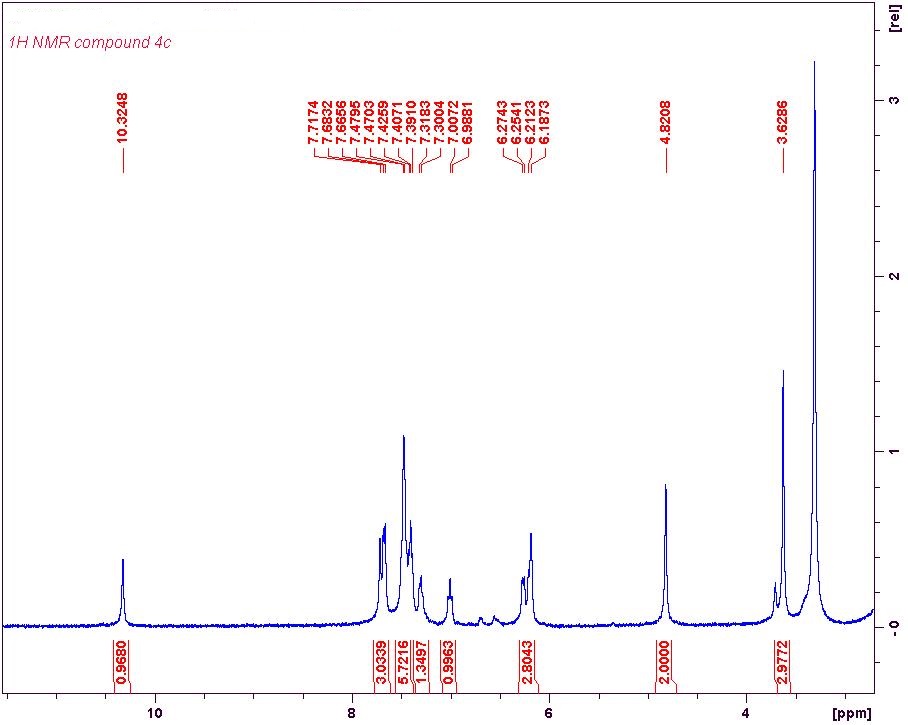


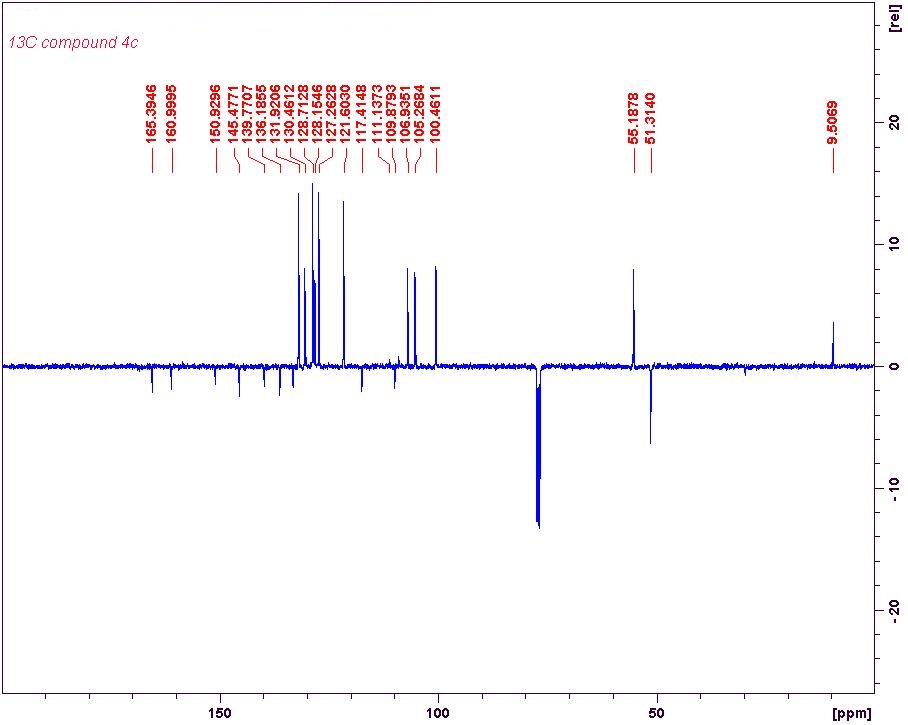


**Figure S3:** ^1^H-NMR and ^13^C-NMR of compound **7a**.


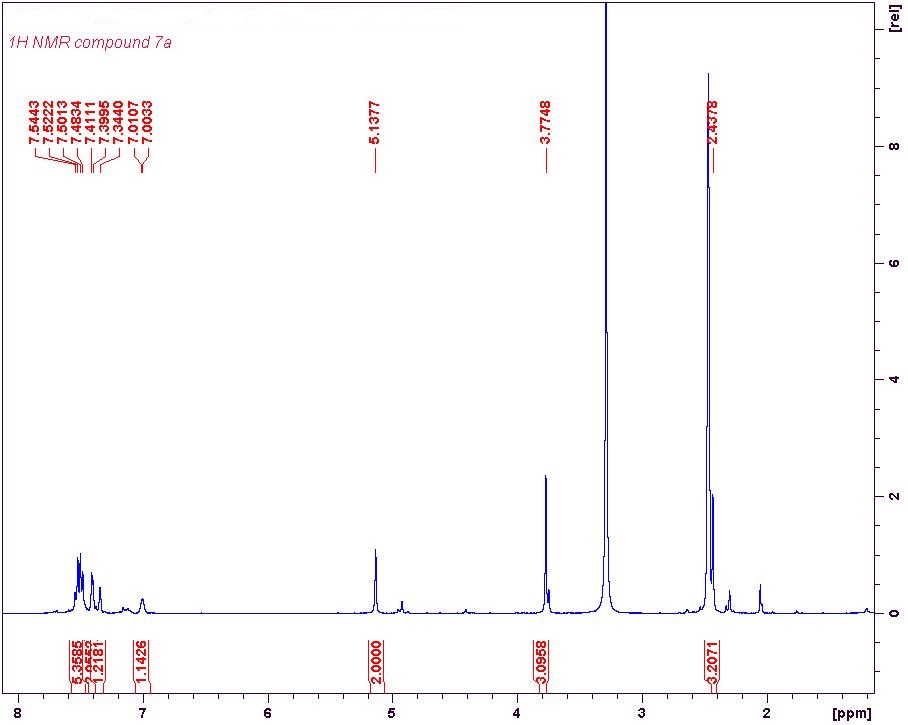


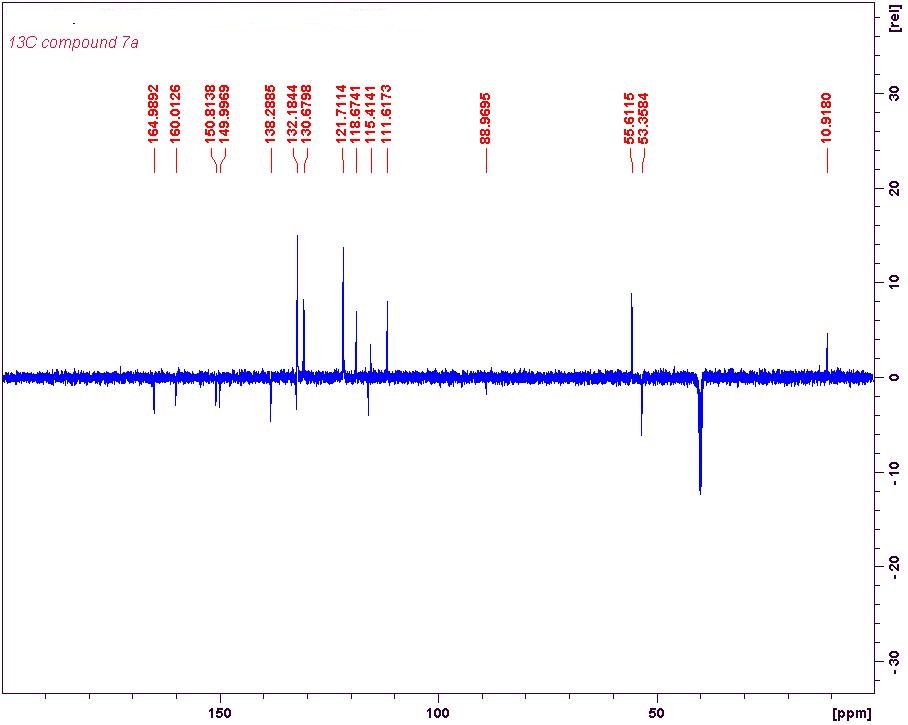


**Figure S4:** ^1^H-NMR and ^13^C-NMR of compound **7b**.


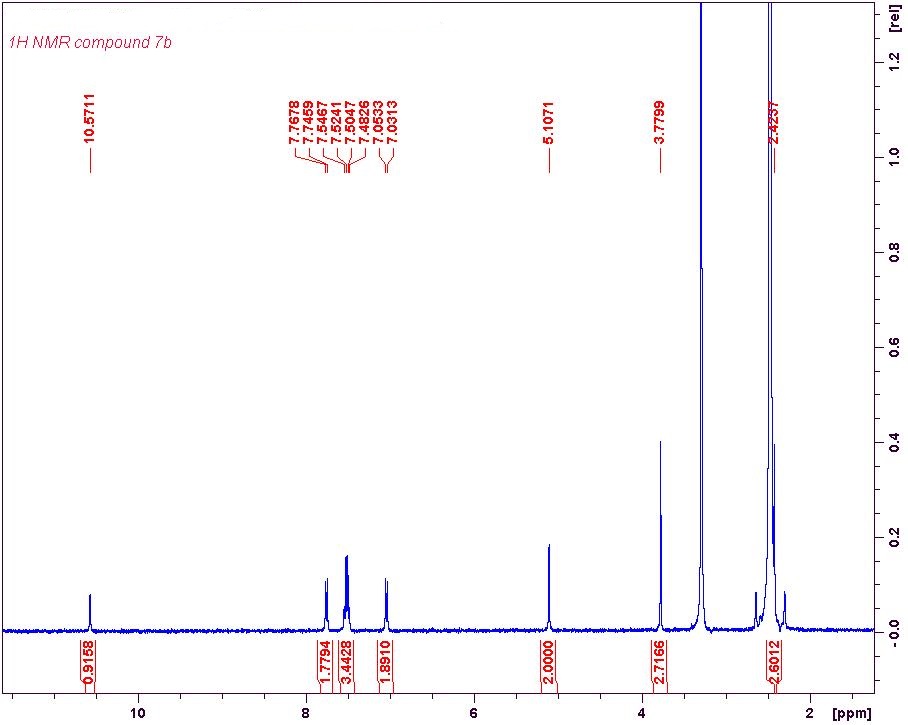


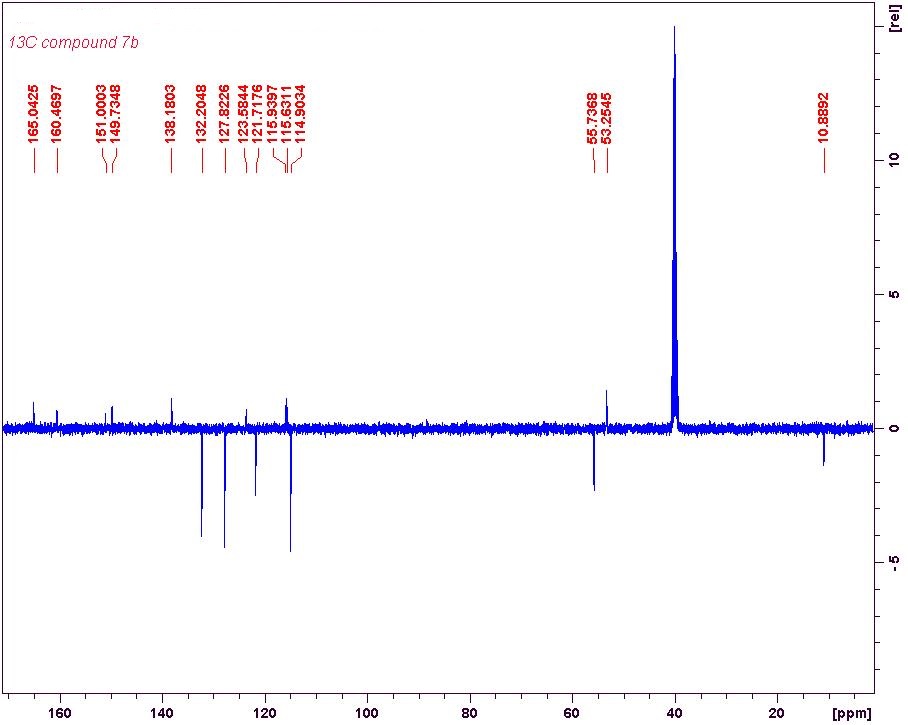


**Figure S5:** ^1^H-NMR and ^13^C-NMR of compound **10**.


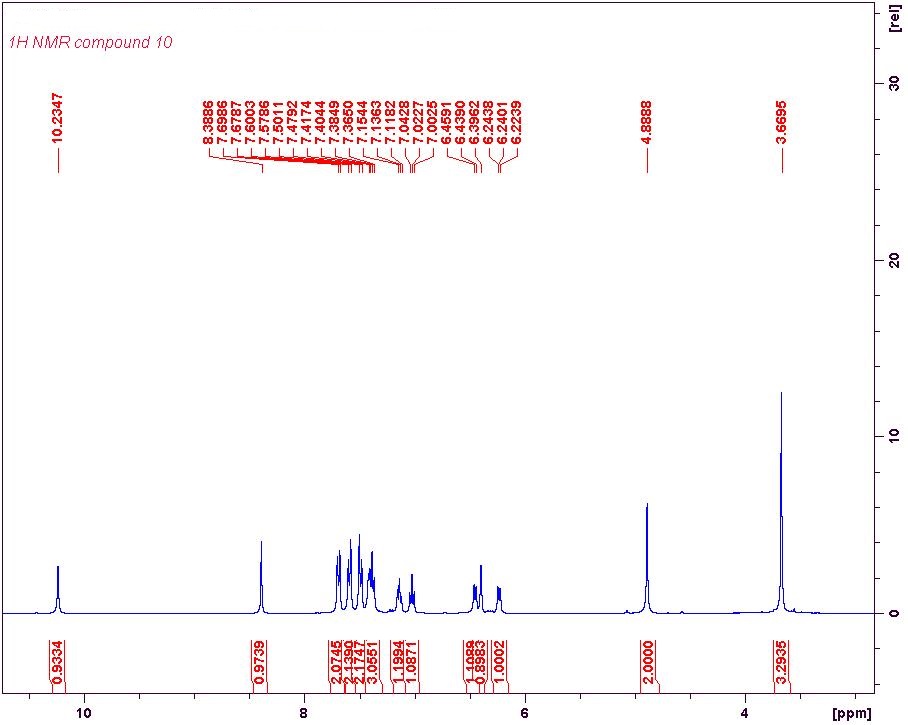


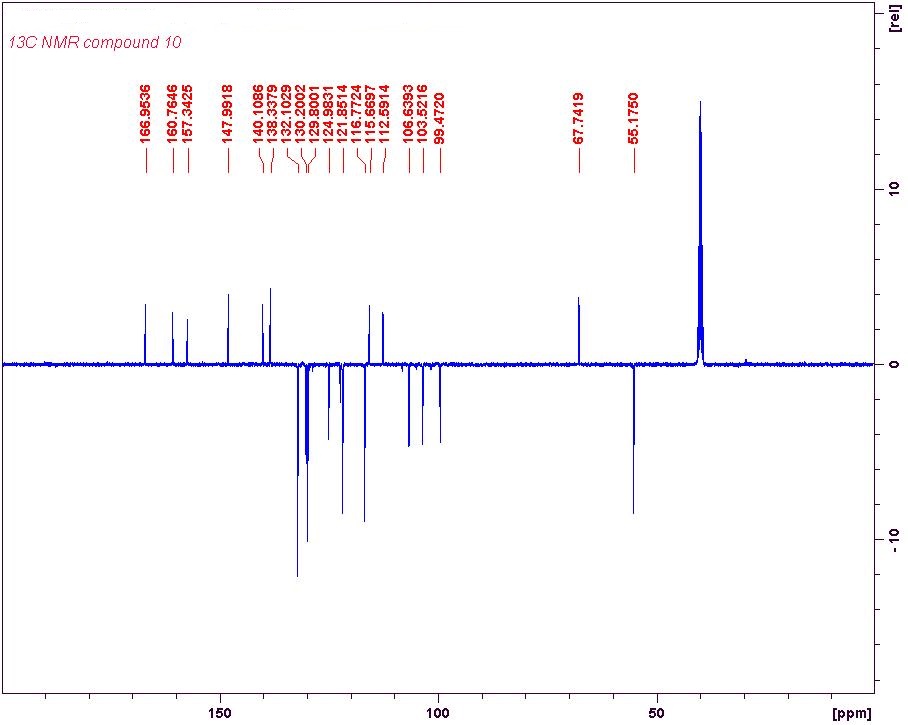


**Figure S6:** ^1^H-NMR and ^13^C-NMR of compound **13a**.


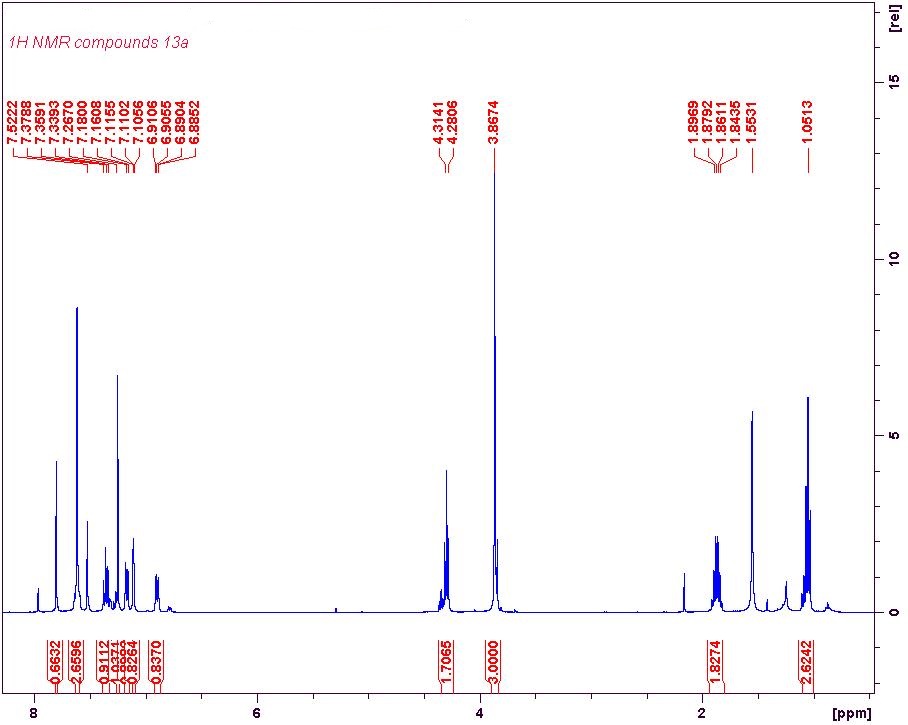


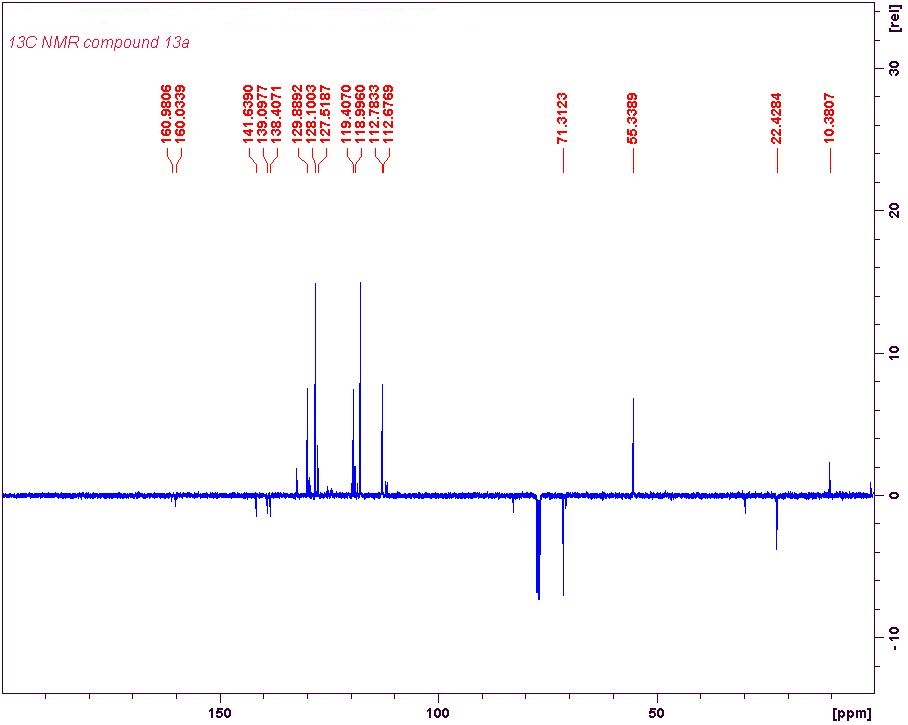


**Figure S7:** ^1^H-NMR and ^13^C-NMR of compound **13d**.


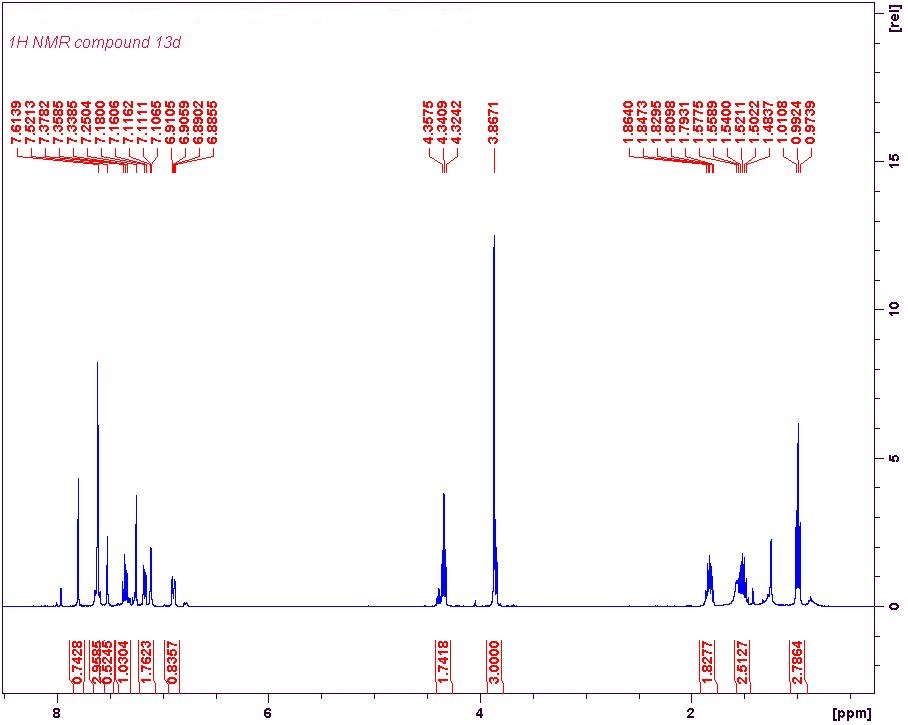


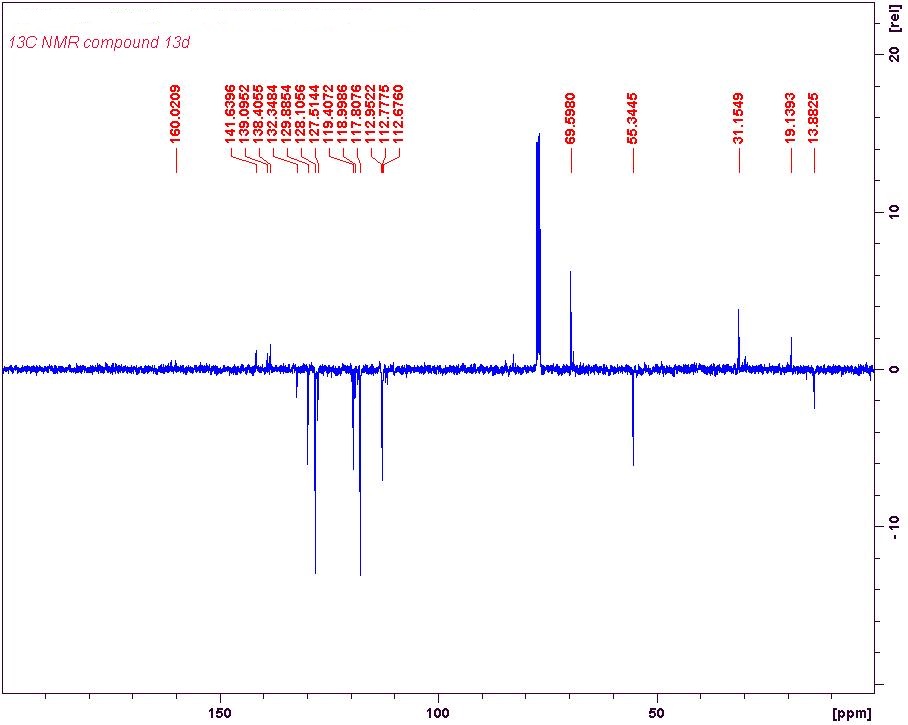


**Figure S8:** ^1^H-NMR and ^13^C-NMR of compound **13f**.


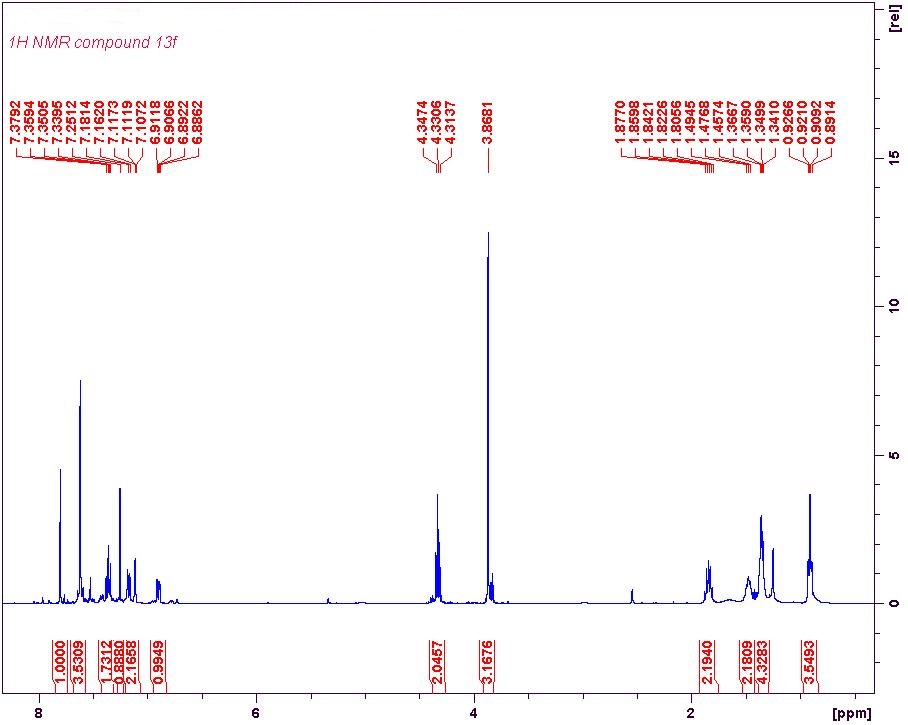


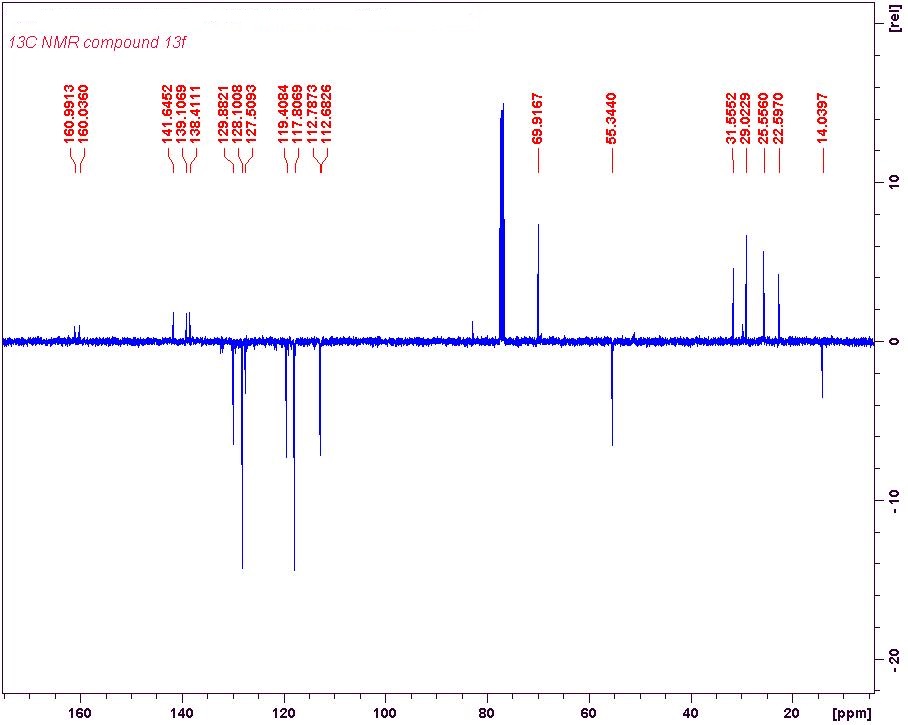


**Figure S9:** ^1^H-NMR, ^13^C-NMR, ^1^H-^13^C HMBC and ^1^H-^13^C HSQC of compound **15**.


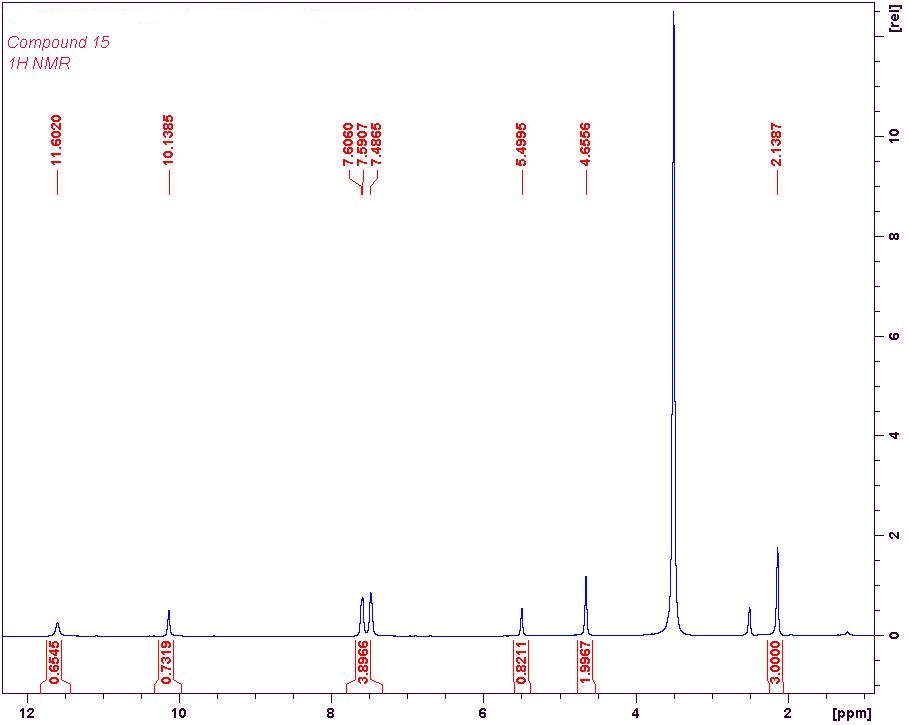


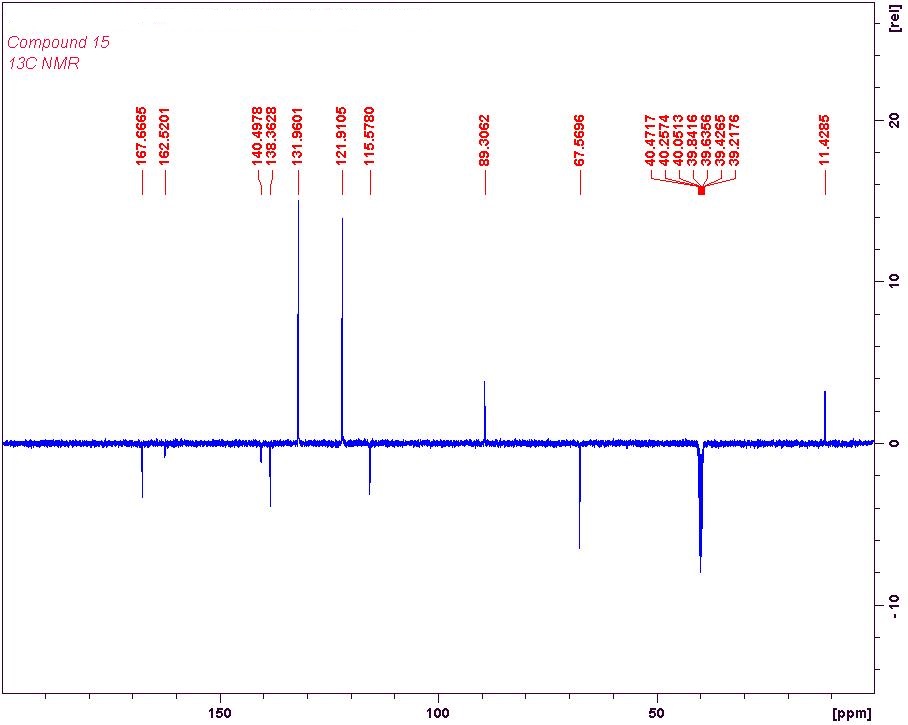


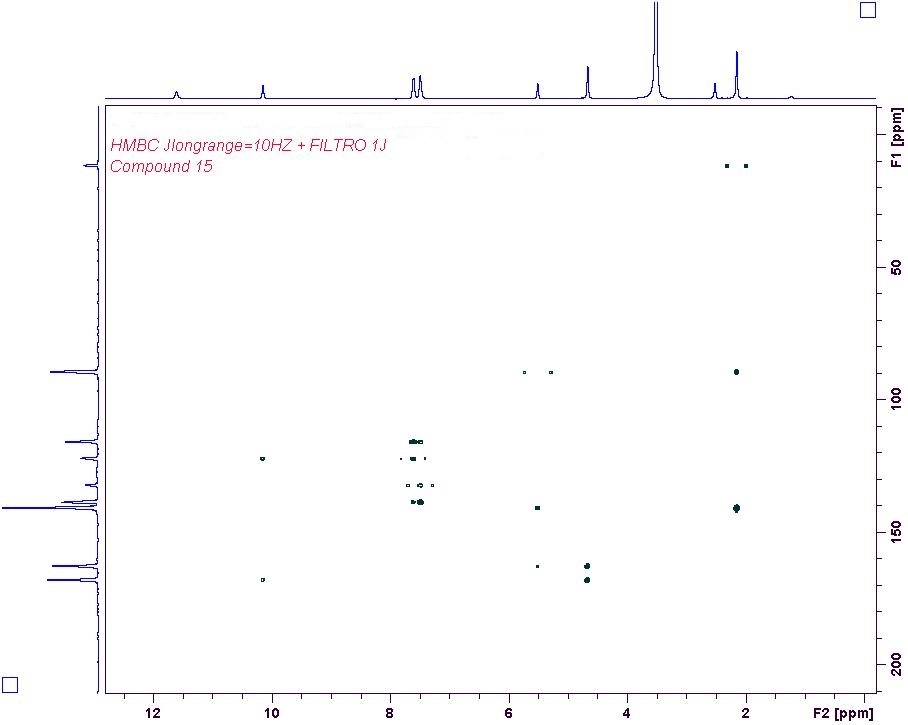


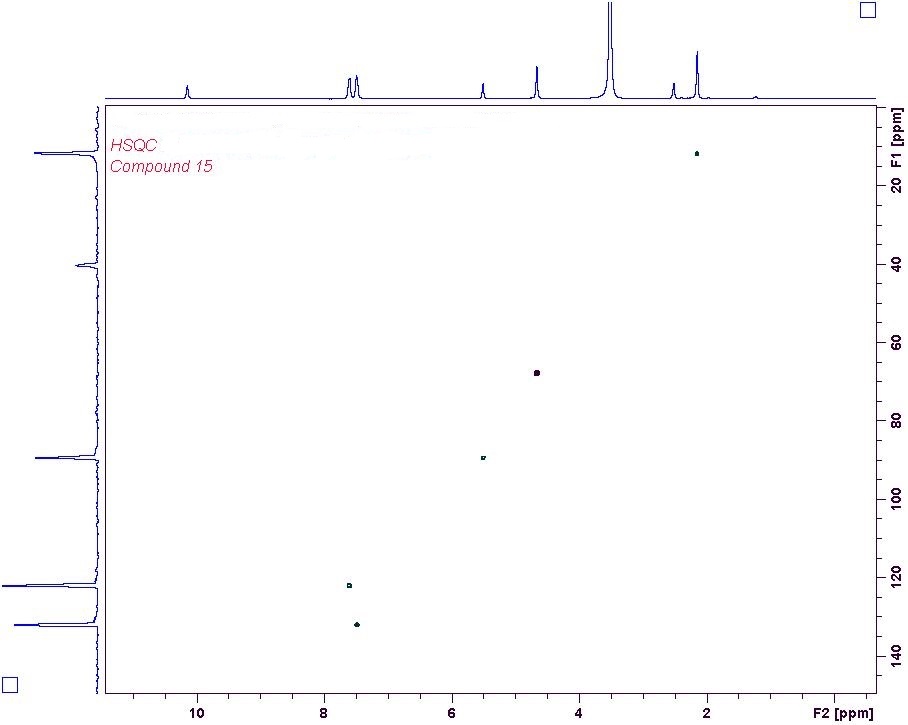


**Figure S10**. Selected view of the crystal packing showing molecules of **15** interacting *via* hydrogen bonds.


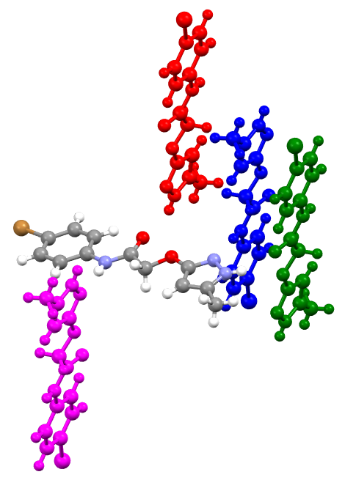


**Table S1**. Selected H-bonds in **15**.

| D-H^…^A | D^…^A (Å) | H^…^A (Å) | D-H^…^A (°) |
| --- | --- | --- | --- |
| N1-H1N^…^O2^1^ | 2.832(5) | 2.05(6) | 159(5) |
| N3-H3N^…^N2^2^ | 2.936(4) | 2.16(5) | 178(5) |

^1^ = -x+1,+y-1/2,-z+3/2; ^2^ = x,-y+1/2,+z-1/2

**Table S2.** Crystallographic data and refinement parameters for compound **15**.

|  | **15** |
| --- | --- |
| Formula | C_12_H_12_N_3_O_2_Br |
| M | 310.16 |
| T (K) | 100 |
| λ (Å) | 1.54184 |
| Crystal system, space group | Monoclinic, P2_1_/c |
| Unit cell dimensions (Å, °) | a = 12.318(1)  b = 8.348(1); β = 100.908(5)  c = 12.172(1) |
| V (Å^3^) | 1229.0(2) |
| Z, ρ (mg/cm3) | 4, 1.1676 |
| μ (mm^-1^) | 4.561 |
| F(000) | 324 |
| 2θ range (°) | 7.3-133.1 |
| Reflns collected / unique (R_int_) | 14021 / 2168 (0.0816) |
| Data / parameters | 2168 / 199 |
| Final R indices [I > 2σ] | R1 = 0.0479, wR2 = 0.1231 |
| R indices (all data) | R1 = 0.0544, wR2 = 0.1287 |
| GoF | 1.117 |

**Figure S11.** Docking pose of **EC10** in FPR1.


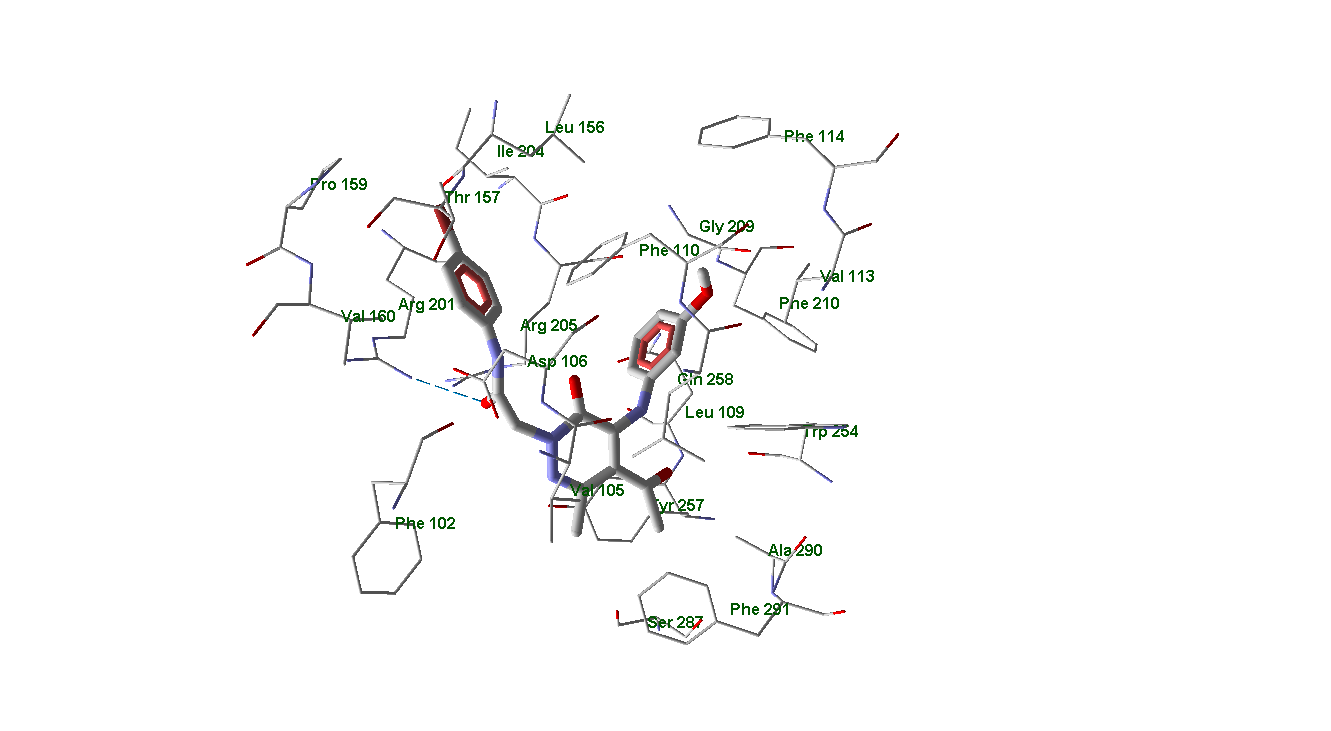


**Figure S12.** Docking pose of **2a** in FPR1.


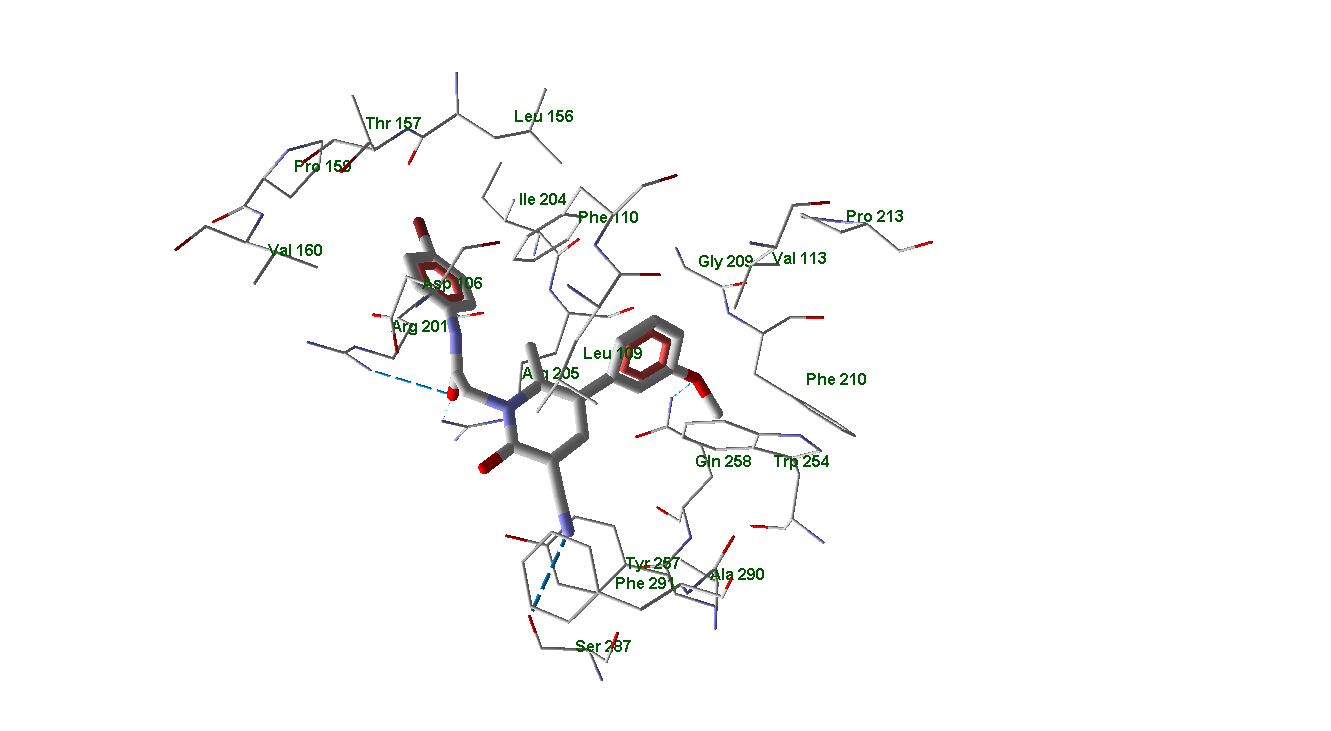


**Figure S13.** Docking pose of **7a** in FPR1.


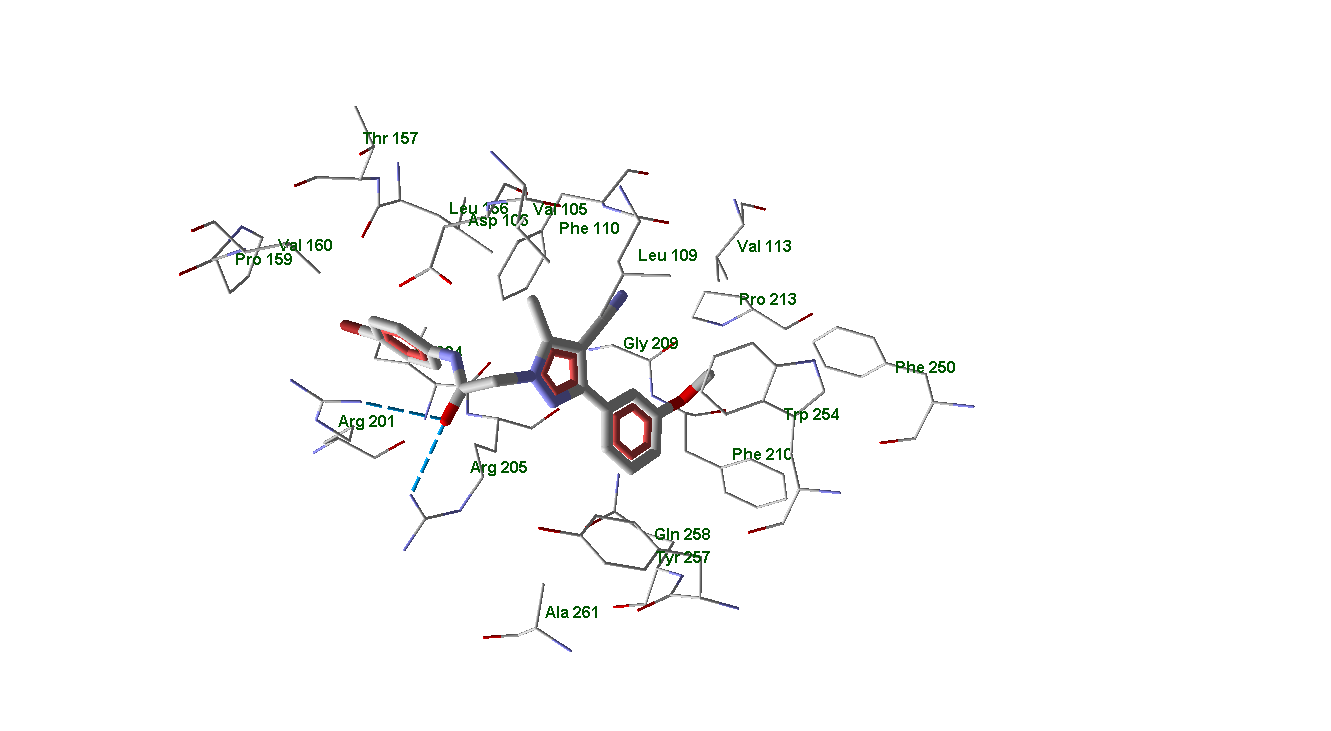


**Figure S14.** Docking pose of **10** in FPR1: a weak H-bond with Arg201 is formed.


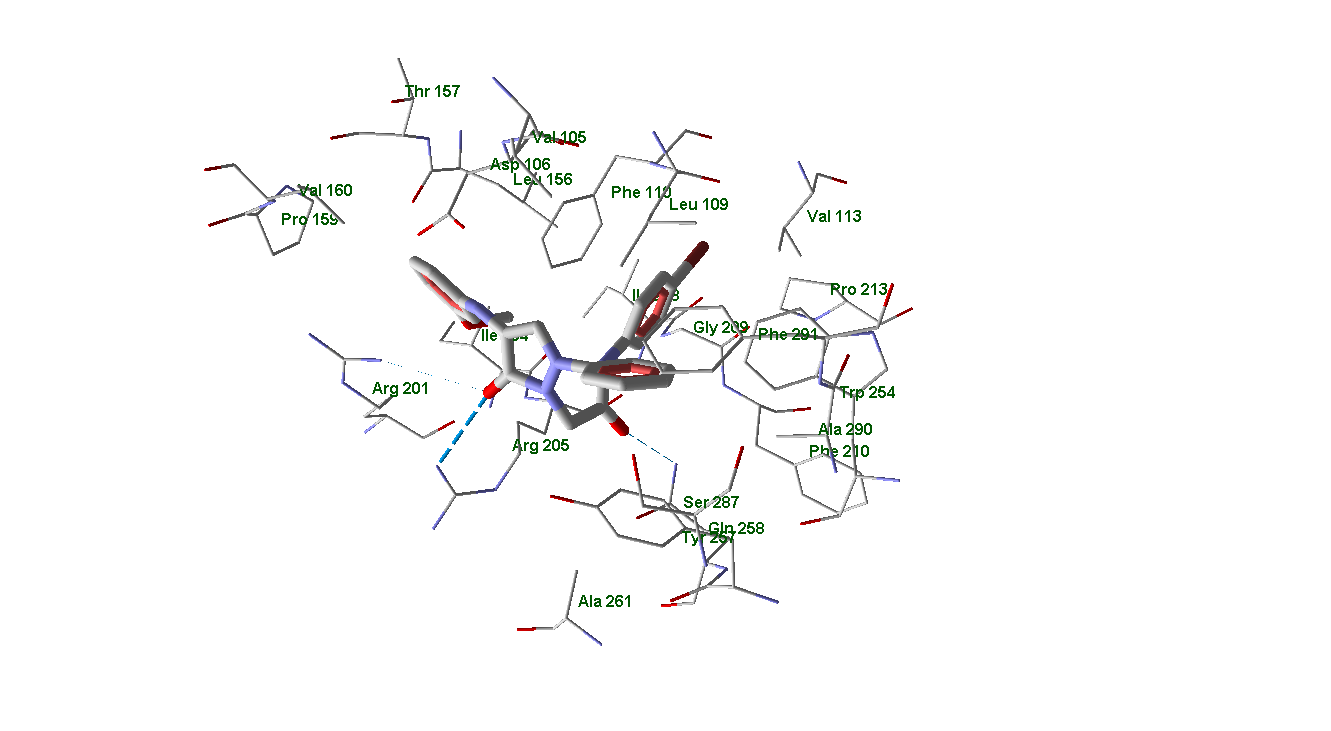


**Figure S15.** Docking pose of **15** in FPR1: a weak H-bond with Arg201 is formed.


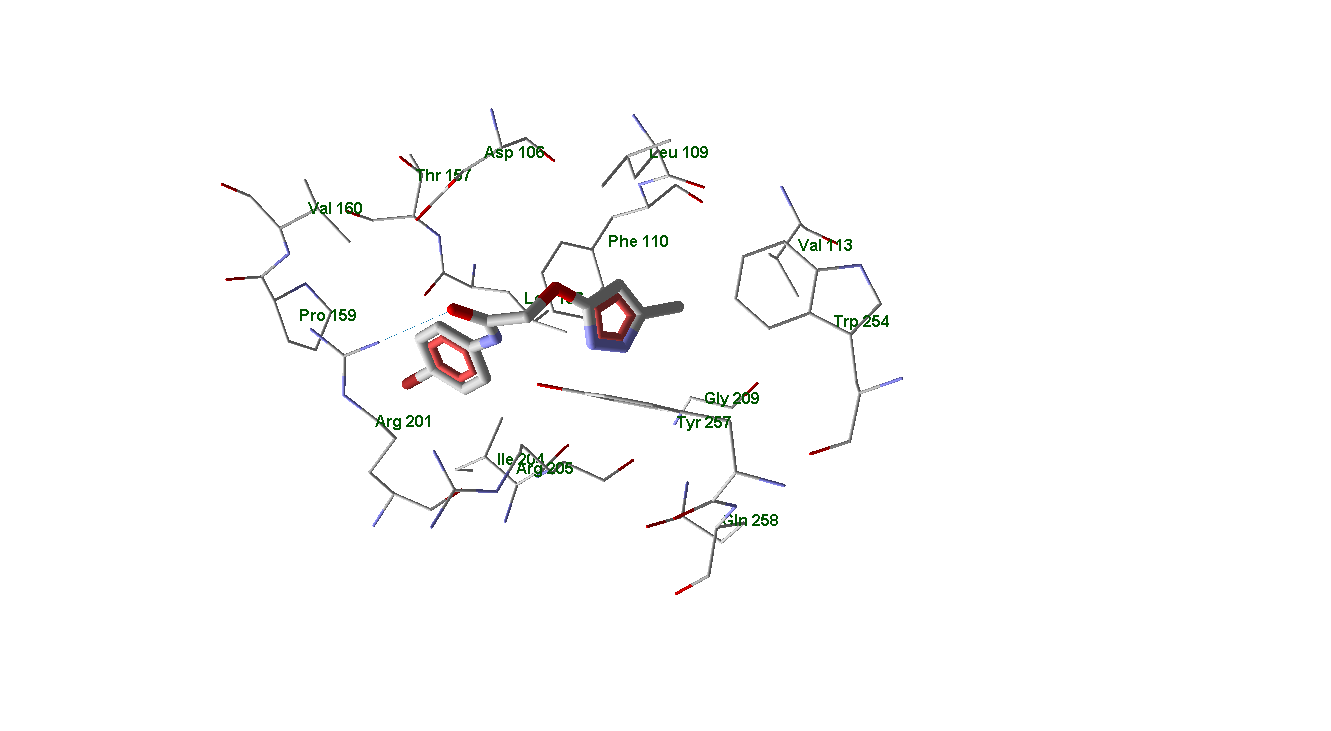


**Figure S16.** Superimposed docking poses of compounds **EC3** (violet), **EC10** (dark-blue), **2a** (light-blue), **4e** (light-yellow), **7a** (dark-yellow), and **15** (orange) in FPR1 binding site. Residues within 3 Å from **EC3** pose are visible. The docked fMLF peptide is shown in thin black sticks.


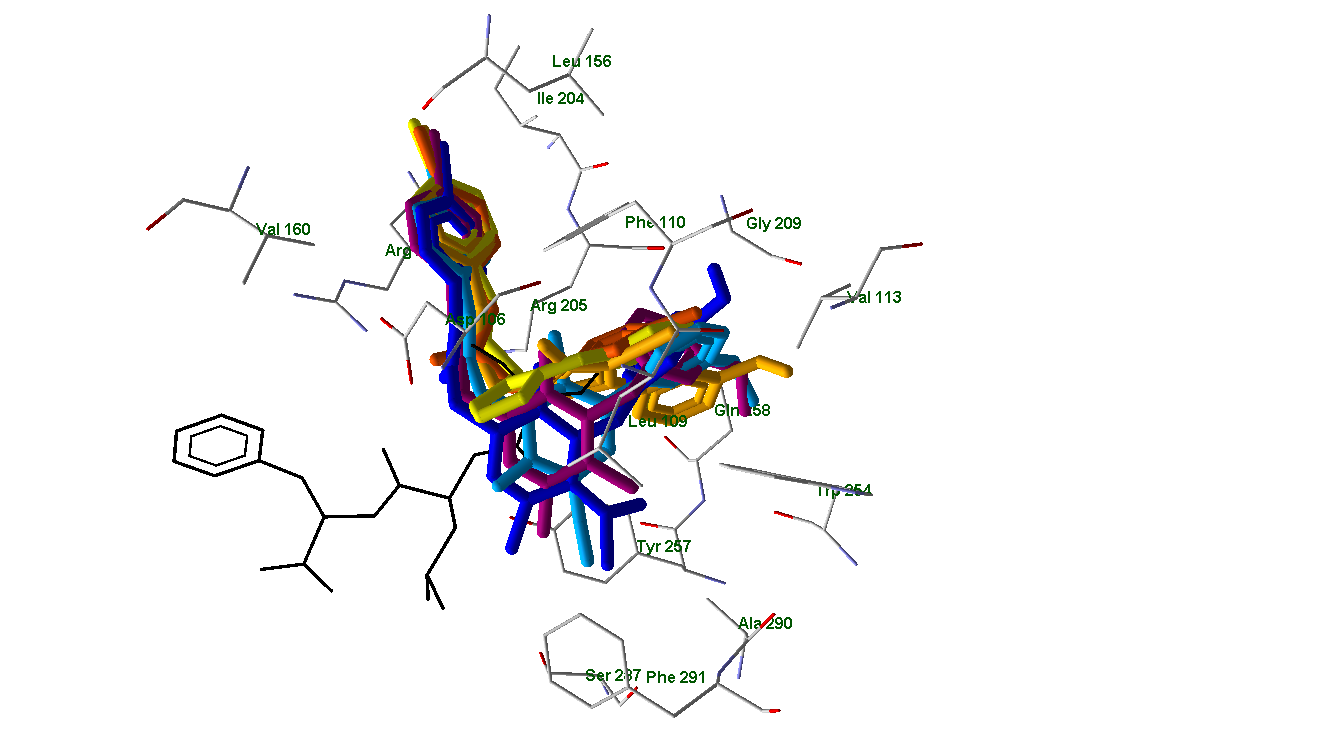


**Figure S17.** Docking poses of **EC10** in FPR2: weak H-bonds are formed with Arg201 and Arg205, slightly visible. Pyridazinone oxygen is involved in both H-bonds.


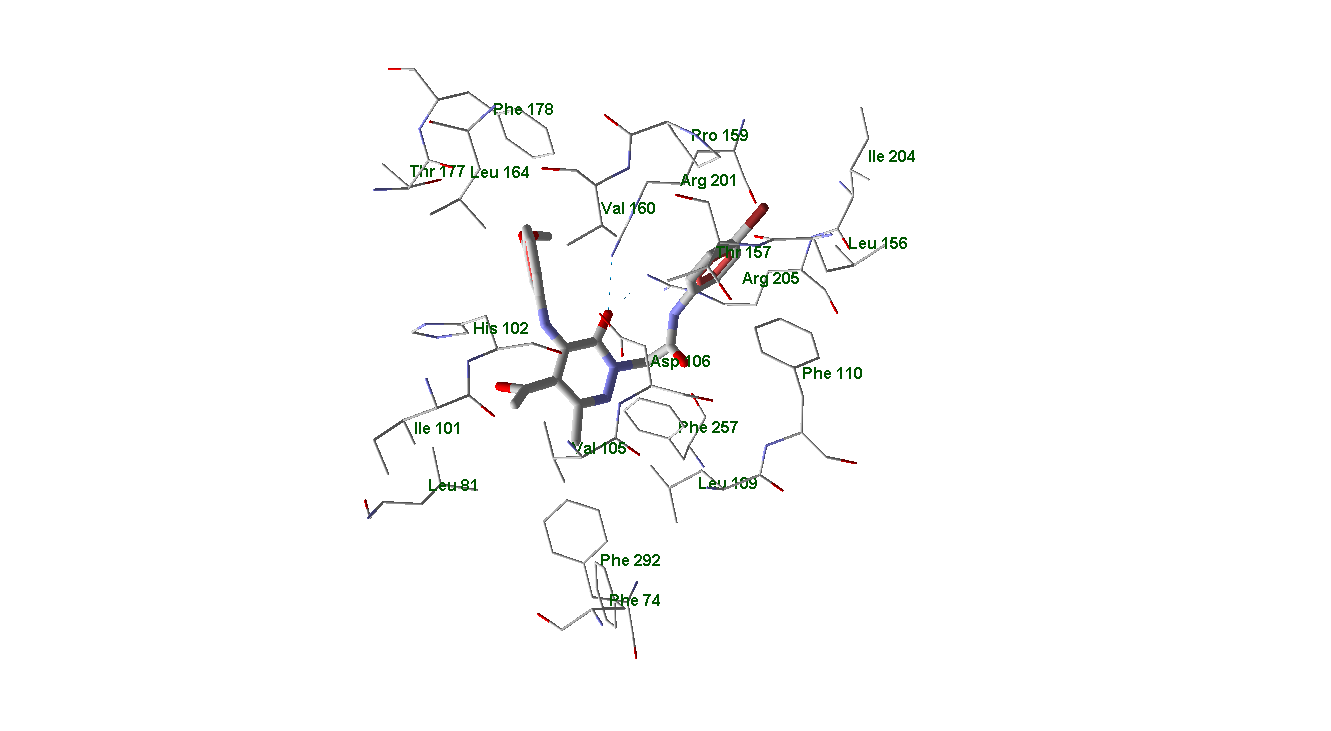


**Figure S18.** Docking pose of **2a** in FPR2: a weak H-bond is formed with Asp106.


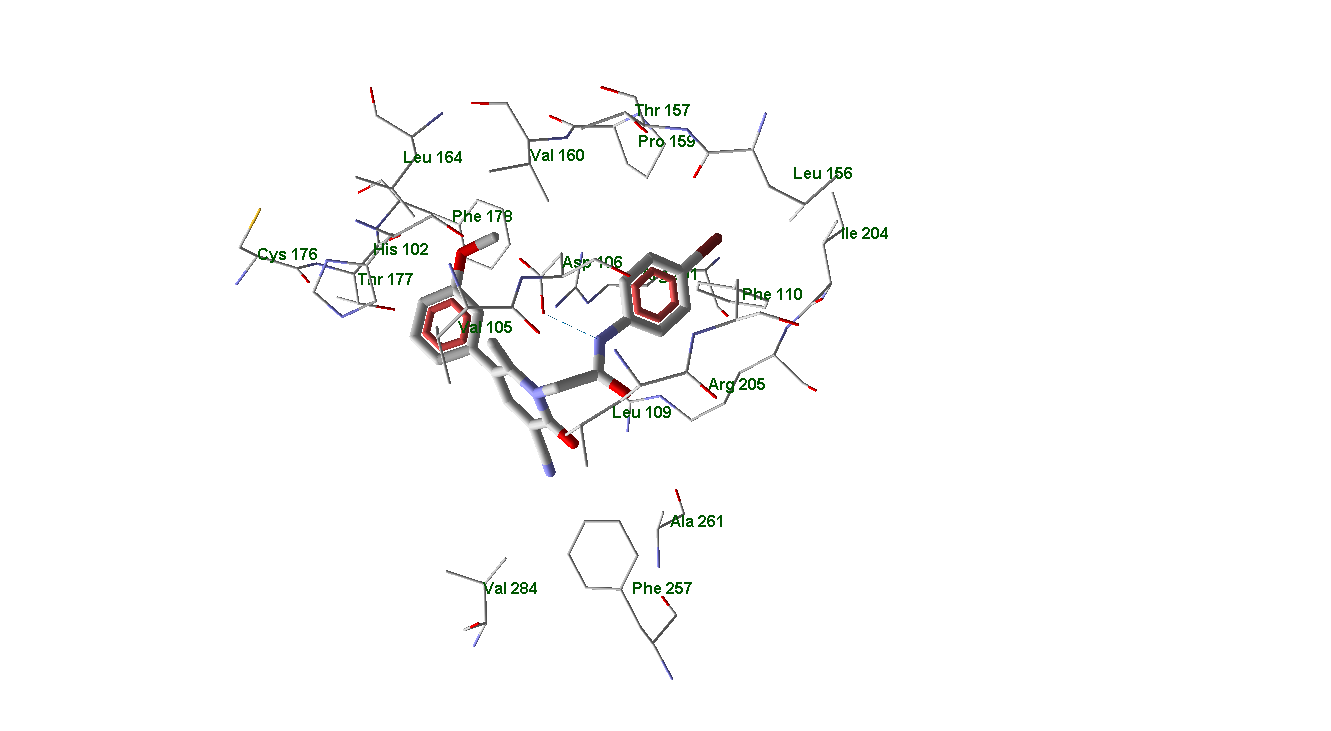


**Figure S19.** Docking poses of **7a** in FPR2: a weak H-bond is formed with Arg205 – slightly visible. Pyrazolone nitrogen is involved in both H-bonds.


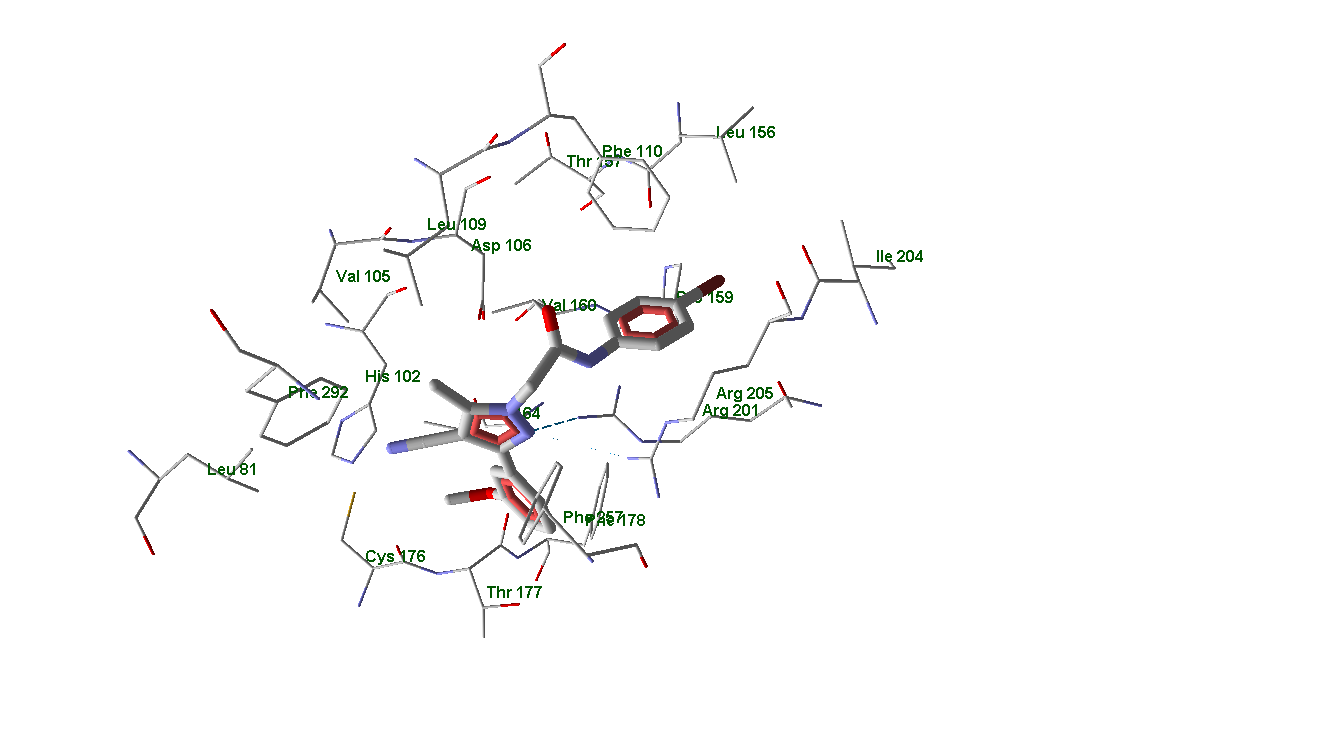


**Figure S20.** Docking pose of **10** in FPR2.


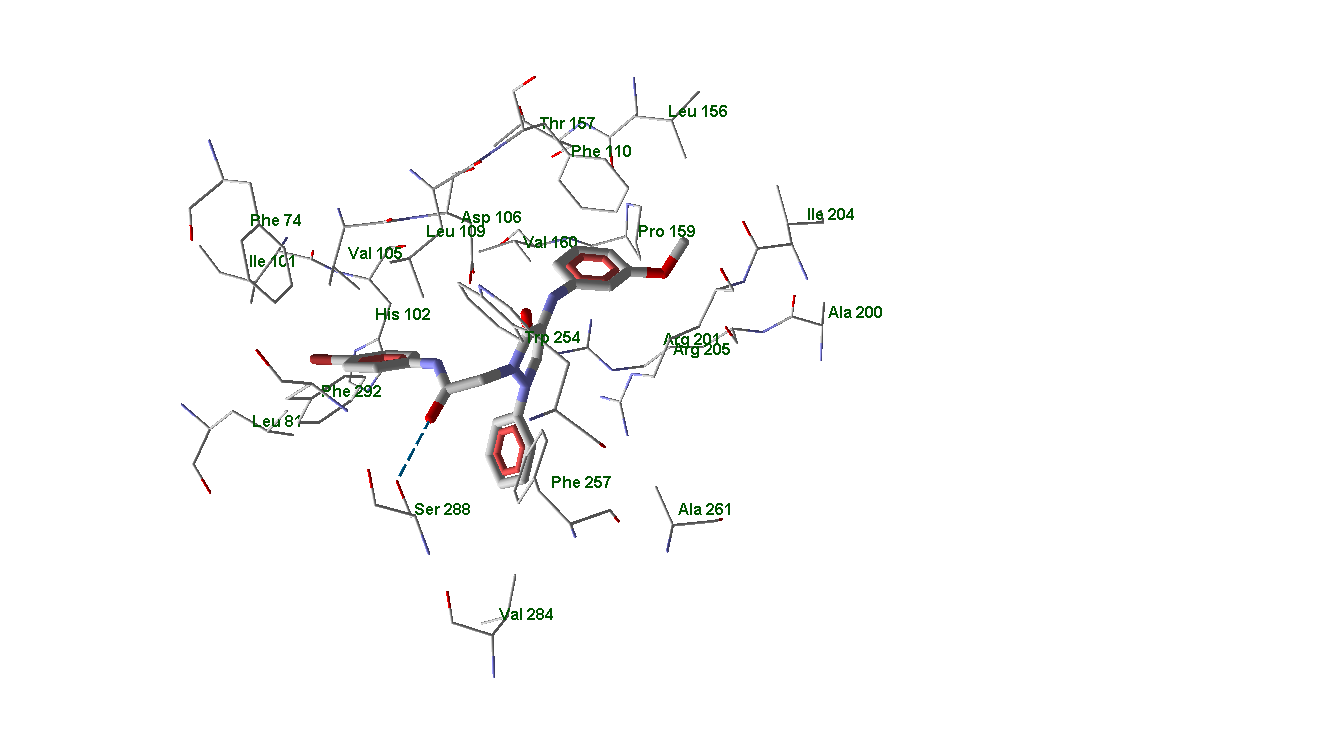


**Figure S21.** Docking pose of **15** in FPR2: a weak H-bond is formed with Arg205.


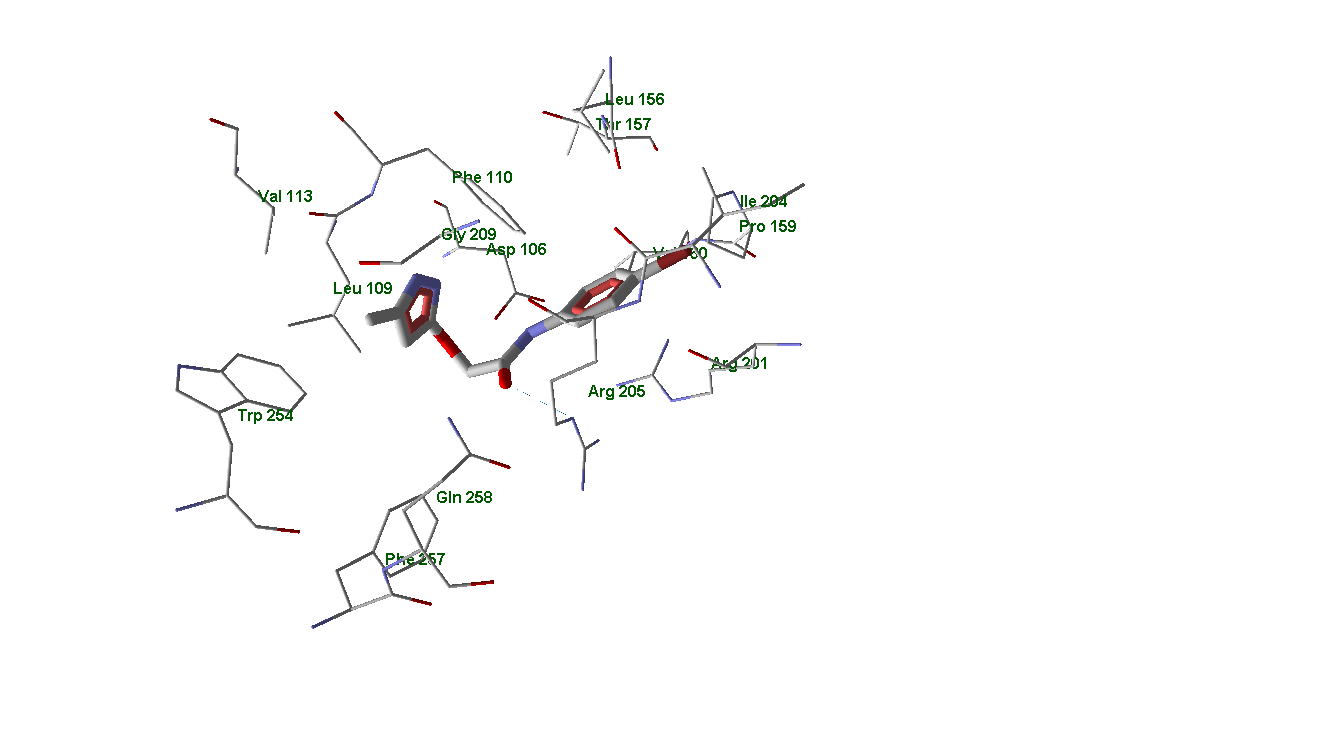


**Figure S22.** Superimposed docking poses of compounds **EC3** (violet), **EC10** (dark-blue), **2a** (light-blue), **4e** (light-yellow), **7a** (dark-yellow), and **15** (orange) in FPR2 binding site (PDB code 6OMM). Residues within 3 Å from **EC3** pose are visible.


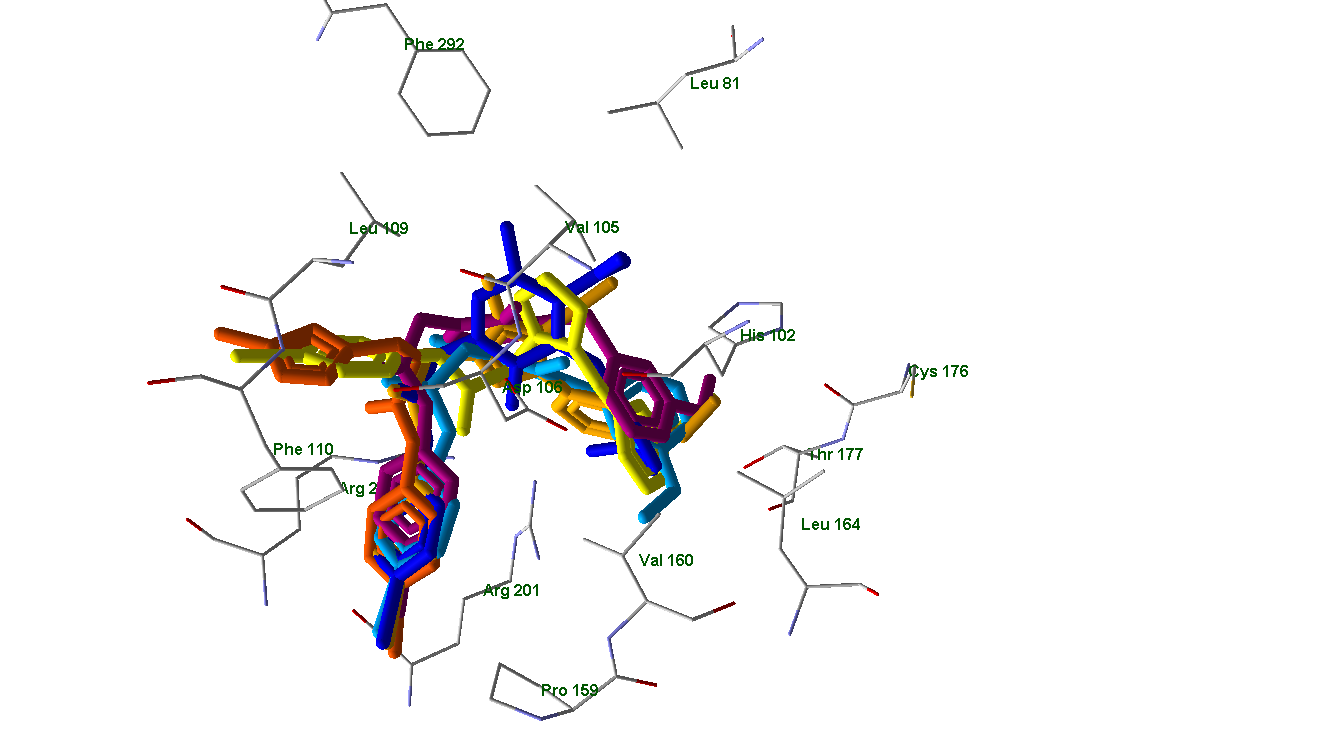


**Table S3.** Analysis of partial docking scores (PDS) for fMLF peptide bound to FPR1; calculated using MolDock scoring functions.

| Residue | ID | MolDock score (PDS) |
| --- | --- | --- |
| Phe | 102 | -27.2499 |
| Arg | 84 | -12.7092 |
| Phe | 110** | -10.3799 |
| Arg | 201** | -9.13452 |
| Phe | 81 | -9.09121 |
| Arg | 205** | -8.76696 |
| Tyr | 257** | -7.9836 |
| Asp | 106* | -7.58535 |
| Leu | 109** | -7.41618 |
| Thr | 177 | -4.7854 |
| Phe | 291* | -4.42991 |
| Val | 164 | -3.45666 |
| Cys | 176 | -3.20467 |
| Trp | 254** | -3.15298 |
| Phe | 178 | -3.0049 |
| Val | 105 | -2.36037 |
| Gly | 209* | -1.78086 |
| Gln | 258** | -1.71924 |
| Ser | 287* | -1.43696 |
| Val | 160 | -1.23861 |

The residues which are met among Top10 residues for poses of potent FPR1 agonists **EC3**, **EC10**, and **2a** are highlighted in green. The residues present among Top10 highly scored residues for poses of at least one moderately active agonist (**4e**, **7a**, or **15**) are marked by blue asterisks. The residues present among Top10 amino acids for the pose of inactive agonist **10** are marked by grey asterisks.

**Table S4.** The top FPR1 residues (with absolute PDS value greater than 1.0) interacting with molecules **EC3**, **EC10**, **2a**, **4e**, **7a**, **15**, and **10** are given in the tables below. The Top10 residues present also in the table for fMLF peptide (see Table S3) are highlighted.

**EC3**

| Residue | ID | MolDock score (PDS) |
| --- | --- | --- |
| Arg | 201 | -22.4472 |
| Phe | 110 | -20.2641 |
| Arg | 205 | -17.2343 |
| Asp | 106 | -14.0472 |
| Tyr | 257 | -11.0719 |
| Leu | 109 | -10.4828 |
| Trp | 254 | -8.60554 |
| Gly | 209 | -7.98607 |
| Phe | 210 | -7.63802 |
| Gln | 258 | -6.46263 |
| Ile | 204 | -6.22004 |
| Leu | 156 | -5.58348 |
| Val | 160 | -2.5493 |
| Pro | 159 | -2.44423 |
| Phe | 291 | -2.17556 |
| Thr | 157 | -1.64901 |
| Val | 113 | -1.4284 |
| Ser | 287 | -1.2002 |

**EC10**

| Residue | ID | MolDock score (PDS) |
| --- | --- | --- |
| Phe | 110 | -26.0508 |
| Arg | 201 | -22.8293 |
| Tyr | 257 | -18.0021 |
| Leu | 109 | -15.9046 |
| Arg | 205 | -15.7324 |
| Trp | 254 | -11.8447 |
| Asp | 106 | -9.92674 |
| Phe | 291 | -6.7635 |
| Leu | 156 | -5.32323 |
| Gly | 209 | -5.11983 |
| Ile | 204 | -4.30546 |
| Ser | 287 | -4.28525 |
| Val | 113 | -4.1237 |
| Gln | 258 | -2.77946 |
| Ala | 290 | -2.58269 |
| Val | 160 | -2.25385 |
| Phe | 210 | -2.18412 |
| Thr | 157 | -1.78104 |
| Pro | 159 | -1.69499 |

**2a**

| Residue | ID | MolDock score (PDS) |
| --- | --- | --- |
| Arg | 201 | -24.362 |
| Phe | 110 | -19.3565 |
| Trp | 254 | -18.9042 |
| Tyr | 257 | -15.2232 |
| Leu | 109 | -15.1923 |
| Arg | 205 | -14.4169 |
| Asp | 106 | -10.2392 |
| Gly | 209 | -6.84904 |
| Phe | 210 | -6.71724 |
| Ile | 204 | -6.33033 |
| Gln | 258 | -5.92576 |
| Ser | 287 | -5.71021 |
| Leu | 156 | -4.92109 |
| Phe | 291 | -4.30793 |
| Val | 113 | -4.13087 |
| Val | 160 | -2.33057 |
| Pro | 159 | -2.28351 |
| Thr | 157 | -1.24906 |
| Ala | 290 | -1.1336 |

**4e**

| Residue | ID | MolDock score (PDS) |
| --- | --- | --- |
| Phe | 110 | -30.0222 |
| Arg | 201 | -27.594 |
| Arg | 205 | -24.0824 |
| Asp | 106 | -9.77703 |
| Trp | 254 | -8.08414 |
| Ile | 204 | -7.59014 |
| Leu | 109 | -7.03071 |
| Gln | 258 | -6.3297 |
| Leu | 156 | -6.30567 |
| Gly | 209 | -5.11219 |
| Val | 105 | -3.30342 |
| Tyr | 257 | -3.16339 |
| Pro | 159 | -3.03889 |
| Val | 113 | -2.54175 |
| Phe | 210 | -1.76692 |
| Val | 160 | -1.14958 |

**7a**

| Residue | ID | MolDock score (PDS) |
| --- | --- | --- |
| Arg | 201 | -26.1878 |
| Phe | 110 | -22.4965 |
| Trp | 254 | -21.2267 |
| Arg | 205 | -21.2133 |
| Asp | 106 | -11.3685 |
| Leu | 109 | -8.97453 |
| Phe | 210 | -8.48512 |
| Ile | 204 | -8.05584 |
| Tyr | 257 | -7.99082 |
| Leu | 156 | -6.06582 |
| Gly | 209 | -4.32514 |
| Val | 113 | -4.04652 |
| Gln | 258 | -3.92967 |
| Pro | 159 | -2.41205 |
| Val | 160 | -1.28362 |
| Thr | 157 | -1.06607 |

**15**

| Residue | ID | MolDock score (PDS) |
| --- | --- | --- |
| Phe | 110 | -27.7272 |
| Arg | 201 | -23.8411 |
| Arg | 205 | -16.8852 |
| Leu | 109 | -8.22948 |
| Ile | 204 | -7.90666 |
| Asp | 106 | -7.73314 |
| Leu | 156 | -5.87435 |
| Trp | 254 | -3.85206 |
| Gly | 209 | -3.22435 |
| Pro | 159 | -2.31356 |
| Val | 113 | -1.75566 |
| Val | 160 | -1.34798 |

**10**

| Residue | ID | MolDock score (PDS) |
| --- | --- | --- |
| Phe | 110 | -32.2104 |
| Arg | 205 | -22.0479 |
| Arg | 201 | -21.0483 |
| Tyr | 257 | -19.5434 |
| Leu | 109 | -14.7112 |
| Trp | 254 | -7.63266 |
| Leu | 156 | -7.07192 |
| Phe | 291 | -6.68751 |
| Ser | 287 | -4.31374 |
| Gln | 258 | -3.94823 |
| Asp | 106 | -3.5482 |
| Thr | 157 | -3.08762 |
| Val | 113 | -2.72186 |
| Phe | 114 | -2.45496 |
| Ala | 290 | -2.21118 |
| Gly | 209 | -1.73692 |
| Ile | 208 | -1.66933 |
| Phe | 210 | -1.66104 |

**Table S5.** Analysis of partial docking scores (PDS) for the experimental conformation of WKYMVm peptide bound to FPR2 (PDB 6OMM). The conformation was treated as a docking pose using MolDock scoring functions.

| Residue | ID | MolDock score (PDS) |
| --- | --- | --- |
| Arg | 205 | -26.7136 |
| Asp | 281 | -17.1388 |
| Glu | 89 | -15.1611 |
| Arg | 201 | -15.0712 |
| Phe | 110 | -14.6308 |
| Thr | 177 | -11.2787 |
| Asp | 106 | -10.3621 |
| Phe | 178 | -8.95635 |
| Leu | 109 | -6.65489 |
| Val | 284 | -6.3689 |
| His | 102 | -6.01137 |
| Phe | 257 | -4.91711 |
| Leu | 198 | -4.45395 |
| Ala | 261 | -4.01877 |
| Met | 271 | -3.66083 |
| Leu | 164 | -3.01316 |
| Asn | 285 | -2.71587 |
| Trp | 254 | -2.41759 |
| Leu | 268 | -2.12056 |
| Leu | 272 | -2.08018 |
| Val | 160 | -1.72268 |
| Phe | 292 | -1.70043 |
| Gly | 264 | -1.39536 |
| Gly | 209 | -1.24511 |
| Val | 105 | -1.15545 |
| Cys | 176 | -1.07871 |
| Val | 113 | -1.04986 |

**Table S6.** Similarly to the FPR1 docking poses, the tables with PDS values for the compounds docked to FPR2 receptor are given below.

**EC3** (in spite of the absence of H-bonds, the compound interacts significantly with some residues)

| Residue | ID | MolDock score (PDS) |
| --- | --- | --- |
| Arg | 201 | -24.042 |
| Arg | 205 | -18.6973 |
| His | 102 | -15.8486 |
| Asp | 106 | -14.9886 |
| Phe | 110 | -13.0425 |
| Thr | 177 | -5.81897 |
| Phe | 257 | -5.38808 |
| Leu | 109 | -4.16015 |
| Val | 160 | -3.72553 |
| Val | 105 | -3.29962 |
| Leu | 156 | -3.03299 |
| Pro | 159 | -2.77691 |
| Phe | 178 | -2.52731 |
| Cys | 176 | -2.11703 |
| Leu | 164 | -1.47415 |
| Ile | 204 | -1.30343 |
| Phe | 292 | -1.13841 |

**EC10**

| Residue | ID | MolDock score (PDS) |
| --- | --- | --- |
| Arg | 201 | -33.1388 |
| Asp | 106 | -19.0656 |
| Arg | 205 | -16.7527 |
| His | 102 | -16.4427 |
| Phe | 110 | -11.9079 |
| Phe | 178 | -7.96179 |
| Leu | 156 | -5.34505 |
| Pro | 159 | -4.33708 |
| Thr | 177 | -4.26915 |
| Leu | 81 | -4.05002 |
| Val | 160 | -3.92958 |
| Leu | 109 | -3.8407 |
| Ile | 204 | -3.47793 |
| Phe | 292 | -2.42797 |
| Phe | 257 | -1.79035 |
| Val | 105 | -1.69577 |

**2a**

| Residue | ID | MolDock score (PDS) |
| --- | --- | --- |
| Arg | 201 | -27.2307 |
| Arg | 205 | -24.262 |
| Phe | 110 | -11.0207 |
| Asp | 106 | -8.98554 |
| Phe | 178 | -8.47006 |
| His | 102 | -7.48127 |
| Thr | 177 | -6.75446 |
| Val | 160 | -5.28718 |
| Leu | 156 | -5.28175 |
| Phe | 257 | -4.78997 |
| Pro | 159 | -4.05141 |
| Ile | 204 | -2.59551 |
| Leu | 109 | -1.53299 |
| Cys | 176 | -1.40101 |

**4e**

| Residue | ID | MolDock score (PDS) |
| --- | --- | --- |
| Asp | 106 | -21.6669 |
| His | 102 | -15.7983 |
| Phe | 110 | -14.5952 |
| Leu | 109 | -14.5027 |
| Arg | 201 | -9.67552 |
| Arg | 205 | -9.28088 |
| Phe | 178 | -8.37942 |
| Thr | 177 | -7.0378 |
| Val | 160 | -5.02105 |
| Trp | 254 | -4.66308 |
| Val | 105 | -4.26847 |
| Phe | 257 | -3.43607 |
| Leu | 164 | -1.84557 |
| Cys | 176 | -1.53906 |
| Val | 113 | -1.27336 |

**7a**

| Residue | ID | MolDock score (PDS) |
| --- | --- | --- |
| Arg | 201 | -29.6114 |
| Arg | 205 | -19.9605 |
| Asp | 106 | -16.1689 |
| Phe | 110 | -12.1271 |
| His | 102 | -7.62206 |
| Thr | 177 | -5.79442 |
| Phe | 178 | -5.70303 |
| Leu | 156 | -5.50821 |
| Pro | 159 | -3.74655 |
| Ile | 204 | -3.43034 |
| Leu | 109 | -3.11828 |
| Val | 105 | -2.9153 |
| Leu | 164 | -2.72989 |
| Phe | 257 | -2.12893 |
| Cys | 176 | -1.28032 |
| Val | 160 | -1.08923 |
| Phe | 292 | -1.059 |

**15**

| Residue | ID | MolDock score (PDS) |
| --- | --- | --- |
| Phe | 110 | -22.3269 |
| Arg | 201 | -20.8231 |
| Arg | 205 | -16.9429 |
| Leu | 109 | -13.4836 |
| Asp | 106 | -11.1065 |
| Trp | 254 | -6.47321 |
| Leu | 156 | -4.86328 |
| Ile | 204 | -3.97706 |
| Pro | 159 | -3.02479 |
| Val | 113 | -2.28802 |
| Phe | 257 | -2.11389 |
| Gly | 209 | -1.50116 |
| Val | 160 | -1.05468 |

**10**

| Residue | ID | MolDock score (PDS) |
| --- | --- | --- |
| Arg | 205 | -24.6285 |
| Arg | 201 | -21.6353 |
| Asp | 106 | -16.8376 |
| Phe | 257 | -11.5688 |
| Phe | 110 | -11.3327 |
| His | 102 | -9.95499 |
| Phe | 292 | -7.29546 |
| Leu | 109 | -6.52427 |
| Leu | 156 | -5.97323 |
| Ser | 288 | -4.62746 |
| Pro | 159 | -4.37665 |
| Ile | 204 | -3.73073 |
| Leu | 81 | -3.10557 |
| Val | 160 | -2.72205 |
| Val | 105 | -2.15546 |
| Ile | 101 | -2.08828 |
| Val | 284 | -2.03096 |
| Thr | 157 | -1.08459 |
